# Supplementary material for: Analysis of animal-to-human translation shows that only 5% of animal-tested therapeutic interventions obtain regulatory approval for human applications
Source: PLoS Biol. 2024 Jun 13;22(6):e3002667. doi: 10.1371/journal.pbio.3002667 (PMC11175415; doi:10.1371/journal.pbio.3002667)
Supplement: S1 Data — (PDF) [file pbio.3002667.s001.pdf]

Documentation of search strategies
University Library search consultation group

Date: August 1 2023

Topic/research question: Bench-to-bedside translation in animal research

Name of researcher(s): Benjamin Ineichen

Librarian(s): Emma-Lotta Säätelä

Databases:

1. Medline (Ovid)
2. Embase (embase.com)
3. Web of Science Core Collection (Clarivate)

Total number of hits

Broader search (incl narrower):

- Before deduplication: 51,541
- After deduplication: 33,169

Narrower search only:

- Before deduplication: 5,115
- After deduplication: 3,230

Comments:

Deduplication based on the method described in:
Bramer, W. M., Giustini, D., de Jonge, G. B., Holland, L., & Bekhuis, T. (2016). De-duplication of database search results for systematic reviews in EndNote. *Journal of the Medical Library Association: JMLA*, 104(3), 240–243. doi:10.3163/1536-5050.104.3.014

One final, extra step was added to compare DOIs.

1. Medline

Interface: Ovid MEDLINE(R) and Epub Ahead of Print, In-Process & Other Non-Indexed Citations and Daily

Date of Search: 17 June 2022

Number of hits:

- Broad: 19078
- Narrow: 1795

Comment: In Ovid, two or more words are automatically searched as phrases; i.e. no quotation marks are needed

Field labels

- exp/ = exploded MeSH term
- / = non exploded MeSH term
- .ti,ab,kf. = title, abstract and author keywords
- adjx = within x words, regardless of order
- * = truncation of word for alternate endings

Database(s): **Ovid MEDLINE(R) and Epub Ahead of Print, In-Process, In-Data-Review & Other Non-Indexed Citations and Daily** 1946 to June 16, 2022
Search Strategy:

#	Searches	Results
1	exp Animal Experimentation/ or exp Models, animal/ or exp Invertebrates/ or Animals/ or exp Animal Population Groups/ or Chordata/ or exp Chordata, nonvertebrate/ or Vertebrates/ or exp Amphibians/ or exp Birds/ or exp Fishes/ or exp Reptiles/ or Mammals/ or Primates/ or exp Artiodactyla/ or exp Carnivora/ or exp Cetacea/ or exp Chiroptera/ or exp Elephants/ or exp Hyraxes/ or exp Insectivora/ or exp Lagomorpha/ or exp Marsupialia/ or exp Monotremata/ or exp Perissodactyla/ or exp Rodentia/ or exp Scandentia/ or exp Sirenia/ or exp Xenarthra/ or Haplorhini/ or exp Strepsirhini/ or exp Platyrrhini/ or exp Tarsii/ or Catarrhini/ or exp Cercopithecidae/ or exp Hylobatidae/ or Hominidae/ or exp Gorilla Gorilla/ or exp Pan Paniscus/ or exp Pan Troglodytes/ or exp Pongo pygmaeus/	7181215
2	(animals or animal or mice or mus or mouse or murine or woodmouse or rats or rat or murinae or muridae or cottonrat or cottonrats or hamster or hamsters or cricetinae or rodentia or rodent or rodents or pigs or pig or swine or swines or piglets or piglet or boar or boars or sus scrofa or ferrets or ferret or polecat or polecats or mustela putorius or guinea pigs or guinea pig or cavia or callithrix or marmoset or marmosets or cebuella or hapale or octodon or chinchilla or chinchillas or gerbillinae or gerbil or gerbils or jird or jirds or merione or meriones or rabbits or rabbit or hares or hare or diptera or flies or fly or dipteral or drosophila or drosophilidae or cats or cat or carus or felis or nematoda or nematode or nematodes or sipunculida or dogs or dog or canine or canines or canis or sheep or sheeps or mouflon or mouflons or ovis or goats or goat or capra or capras or rupicapra or rupicapras or chamois or haplorhini or monkey or monkeys or anthropoidea or anthropoids or saguinus or tamarin or tamarins or leontopithecus or hominidae or ape or apes or panpaniscus or bonobo or bonobos or pan troglodytes or gibbon or gibbons or siamang or siamangs or nomascus or symphalangus or	5469654

	chimpanzee or chimpanzees or prosimian or prosimians or bush baby or bush babies or galagos or galago or pongidae or gorilla or gorillas or pongo pygmaeus or orangutan or orangutans or lemur or lemurs or lemuridae or horse or horses or equus or cow or calf or bull or chicken or chickens or gallus or quail or bird or birds or quails or poultry or poultries or fowl or fowls or reptile or reptilia or reptiles or snakes or snake or lizard or lizards or alligator or alligators or crocodile or crocodiles or turtle or turtles or amphibian or amphibians or amphibia or frog or frogs or bombina or salientia or toad or toads or epidalea calamita or salamander or salamanders or eel or eels or fish or fishes or pisces or catfish or catfishes or siluriformes or arius or heteropneustes or sheatfish or perch or perches or percidae or perca or trout or trouts or char or chars or salvelinus or minnow or cyprinidae or carps or carp or zebrafish or zebrafishes or goldfish or goldfishes or guppy or guppies or chub or chubs or tinca or barbels or barbus or pimephales or promelas or poecilia reticulata or mullet or mullets or eel or eels or seahorse or seahorses or mugil curema or atlantic cod or shark or sharks or catshark or anguilla or salmonid or salmonids or whitefish or whitefishes or salmon or salmons or sole or solea or lamprey or lampreys or pumpkinseed or sunfish or sunfishes or tilapia or tilapias or turbot or turbots or flatfish or flatfishes or sciuridae or squirrel or squirrels or chipmunk or chipmunks or suslik or susliks or vole or voles or lemming or lemmings or muskrat or muskrats or lemmus or otter or otters or marten or martens or martes or weasel or badger or badgers or ermine or mink or minks or sable or sables or gulo or gulos or wolverine or wolverines or mustela or llama or llamas or alpaca or alpacas or camelid or camelids or guanaco or guanacos or chiroptera or chiropteras or bat or bats or fox or foxes or iguana or iguanas or xenopus laevis or parakeet or parakeets or parrot or parrots or donkey or donkeys or mule or mules or zebra or zebras or shrew or shrews or bison or bisons or buffalo or buffaloes or deer or deers or bear or bears or panda or pandas or wild hog or wild boar or fitchew or fitch or beaver or beavers or jerboa or jerboas or capybara or capybaras or canine or bovine or porcine or hog or hogs).ti,ab,kf.	
3	1 or 2	8057458
4	systematic review.pt.	198997
5	meta analysis.pt.	162272
6	((systematic or scoping) and review).ti.	202301
7	(meta analy* or metaanaly*).ti.	156919
8	or/4-7	348602
9	3 and 8	19078
10	Translational Research, Biomedical/	12661
11	Translational Science, Biomedical/	155
12	(exploit* or translat*).ti,ab,kf.	521595

13	((preclinical* or pre-clinical*) and clinical*).ti,ab,kf.	90733
14	or/10-13	603930
15	9 and 14	1795

Karolinska Institutet

2. Embase

Interface: embase.com<br><br>Date of Search: 17 June 2022<br><br>Number of hits:<br>- Broad: 18359<br>- Narrow: 2077<br><br>Comment: Emtree is the controlled vocabulary in Embase	Field labels<br><br>• /exp = exploded Emtree term<br>• /de = non exploded Emtree term<br>• ti,ab,kw = title, abstract and author keywords<br>• NEAR/x = within x words, regardless of order<br>• * = truncation of word for alternate endings

No.	Query	Results
#15	#10 AND #14	2,077
#14	#11 OR #12 OR #13	629,715
#13	(**preclinical***:ti,ab,kw OR **'pre clinical*'**:ti,ab,kw) AND **clinical****:ti,ab,kw	139,900
#12	**exploit**:ti,ab,kw OR **translat***:ti,ab,kw	499,843
#11	**'translational research'**/exp OR **'translational science'**/exp	20,612
#10	#3 AND #9	18,359
#9	#4 OR #5 OR #6 OR #7 OR #8	526,317
#8	**meta**:ti AND **analy***:ti OR **metaanaly***:ti	192,784
#7	(**systematic**:ti OR **scoping**:ti) AND **review**:ti	235,164
#6	(**systematic**:ti OR **scoping**:ti) AND **review**:ti	235,164

Karolinska Institutet

	**246,160**
**#5**	
**'meta analysis'**/de	
	**347,491**
**#4**	
**'systematic review'**/de	
	**7,926,770**
**#3**	
#**1** OR #**2**	
	**7,603,997**
**#2**	
**'animal experiment'**/exp OR **'animal model'**/exp OR **'invertebrate'**/exp OR **'animal'**/de OR **'experimental animal'**/exp OR **'transgenic animal'**/exp OR **'male animal'**/exp OR **'female animal'**/exp OR **'juvenile animal'**/exp OR **'chordata'**/de OR **'vertebrate'**/de OR **'tetrapod'**/de OR **'fish'**/exp OR **'amniote'**/de OR **'amphibia'**/exp OR **'mammal'**/de OR **'reptile'**/exp OR **'sauropsid'**/exp OR **'therian'**/de OR **'monotremate'**/exp OR **'placental mammals'**/de OR **'marsupial'**/exp OR **'euarchontoglires'**/de OR **'afrotheria'**/exp OR **'boreoeutheria'**/exp OR **'laurasiatheria'**/exp OR **'xenarthra'**/exp OR **'primate'**/de OR **'dermoptera'**/exp OR **'glires'**/exp OR **'scandentia'**/exp OR **'haplorhini'**/de OR **'prosimian'**/exp OR **'simian'**/de OR **'tarsiiform'**/exp OR **'catarrhini'**/de OR **'platyrrhini'**/exp OR **'ape'**/de OR **'cercopithecidae'**/exp OR **'hominid'**/de OR **'hylobatidae'**/exp OR **'chimpanzee'**/exp OR **'gorilla'**/exp OR **'orangutan'**/exp	
	**1,026,894**
**#1**	
(**animals**:ti,ab,kw OR **animal**:ti,ab,kw OR **mice**:ti,ab,kw OR **mus**:ti,ab,kw OR **mouse**:ti,ab,kw OR **murine**:ti,ab,kw OR **woodmouse**:ti,ab,kw OR **rats**:ti,ab,kw OR **rat**:ti,ab,kw OR **murinae**:ti,ab,kw OR **muridae**:ti,ab,kw OR **cottonrat**:ti,ab,kw OR **cottonrats**:ti,ab,kw OR **hamster**:ti,ab,kw OR **hamsters**:ti,ab,kw OR **cricetinae**:ti,ab,kw OR **rodentia**:ti,ab,kw OR **rodent**:ti,ab,kw OR **rodents**:ti,ab,kw OR **pigs**:ti,ab,kw OR **pig**:ti,ab,kw OR **swine**:ti,ab,kw OR **swines**:ti,ab,kw OR **piglets**:ti,ab,kw OR **piglet**:ti,ab,kw OR **boar**:ti,ab,kw OR **boars**:ti,ab,kw OR **'sus scrofa'**:ti,ab,kw OR **ferrets**:ti,ab,kw OR **ferret**:ti,ab,kw OR **polecat**:ti,ab,kw OR **polecats**:ti,ab,kw OR **'mustela putorius'**:ti,ab,kw OR **'guinea pigs'**:ti,ab,kw OR **'guinea pig'**:ti,ab,kw OR **cavia**:ti,ab,kw OR **callithrix**:ti,ab,kw OR **marmoset**:ti,ab,kw OR **marmosets**:ti,ab,kw OR **cebuella**:ti,ab,kw OR **hapale**:ti,ab,kw OR **octodon**:ti,ab,kw OR **chinchilla**:ti,ab,kw OR **chinchillas**:ti,ab,kw OR **gerbillinae**:ti,ab,kw OR **gerbil**:ti,ab,kw OR **gerbils**:ti,ab,kw OR **jird**:ti,ab,kw OR **jirds**:ti,ab,kw OR **merione**:ti,ab,kw OR **meriones**:ti,ab,kw OR **rabbits**:ti,ab,kw OR **rabbit**:ti,ab,kw OR **hares**:ti,ab,kw OR **hare**:ti,ab,kw OR **diptera**:ti,ab,kw OR **flies**:ti,ab,kw OR **fly**:ti,ab,kw OR **dipteral**:ti,ab,kw OR **drosophila**:ti,ab,kw OR **drosophilidae**:ti,ab,kw OR **cats**:ti,ab,kw OR **cat**:ti,ab,kw OR **carus**:ti,ab,kw OR **felis**:ti,ab,kw OR **nematoda**:ti,ab,kw OR **nematode**:ti,ab,kw OR **nematodes**:ti,ab,kw OR **sipunculida**:ti,ab,kw OR **dogs**:ti,ab,kw OR **dog**:ti,ab,kw OR **canine**:ti,ab,kw OR **canines**:ti,ab,kw OR **canis**:ti,ab,kw OR **sheep**:ti,ab,kw OR **sheeps**:ti,ab,kw OR **mouflon**:ti,ab,kw OR **mouflons**:ti,ab,kw OR **ovis**:ti,ab,kw OR **goats**:ti,ab,kw OR **goat**:ti,ab,kw OR **capra**:ti,ab,kw OR **capras**:ti,ab,kw OR **rupicapra**:ti,ab,kw OR **rupicapras**:ti,ab,kw OR **chamois**:ti,ab,kw OR **haplorhini**:ti,ab,kw OR **monkey**:ti,ab,kw OR **monkeys**:ti,ab,kw OR **anthropoidea**:ti,ab,kw OR **anthropoids**:ti,ab,kw OR **saguinus**:ti,ab,kw OR **tamarin**:ti,ab,kw OR **tamarins**:ti,ab,kw OR **leontopithecus**:ti,ab,kw OR **hominidae**:ti,ab,kw OR **ape**:ti,ab,kw OR **apes**:ti,ab,kw OR **panpaniscus**:ti,ab,kw OR **bonobo**:ti,ab,kw OR **bonobos**:ti,ab,kw OR **'pan troglodytes'**:ti,ab,kw OR **gibbon**:ti,ab,kw OR **gibbons**:ti,ab,kw OR **siamang**:ti,ab,kw OR **siamangs**:ti,ab,kw OR **nomascus**:ti,ab,kw OR **symphalangus**:ti,ab,kw OR **chimpanzee**:ti,ab,kw OR **chimpanzees**:ti,ab,kw OR **prosimian**:ti,ab,kw OR **prosimians**:ti,ab,kw OR **'bush baby'**:ti,ab,kw OR **'bush babies'**:ti,ab,kw OR **galagos**:ti,ab,kw OR **galago**:ti,ab,kw OR **pongidae**:ti,ab,kw OR **gorilla**:ti,ab,kw OR **gorillas**:ti,ab,kw OR **'pongo pygmaeus'**:ti,ab,kw OR **orangutan**:ti,ab,kw OR **orangutans**:ti,ab,kw	

OR **lemur**:ti,ab,kw OR **lemurs**:ti,ab,kw OR **lemuridae**:ti,ab,kw OR **horse**:ti,ab,kw OR **horses**:ti,ab,kw OR **equus**:ti,ab,kw OR **cow**:ti,ab,kw OR **calf**:ti,ab,kw OR **bull**:ti,ab,kw OR **chicken**:ti,ab,kw OR **chickens**:ti,ab,kw OR **gallus**:ti,ab,kw OR **quail**:ti,ab,kw OR **bird**:ti,ab,kw OR **birds**:ti,ab,kw OR **quails**:ti,ab,kw OR **poultry**:ti,ab,kw OR **poultries**:ti,ab,kw OR **fowl**:ti,ab,kw OR **fowls**:ti,ab,kw OR **reptile**:ti,ab,kw OR **reptilia**:ti,ab,kw OR **reptiles**:ti,ab,kw OR **snakes**:ti,ab,kw OR **snake**:ti,ab,kw OR **lizard**:ti,ab,kw OR **lizards**:ti,ab,kw OR **alligator**:ti,ab,kw OR **alligators**:ti,ab,kw OR **crocodile**:ti,ab,kw OR **crocodiles**:ti,ab,kw OR **turtle**:ti,ab,kw OR **turtles**:ti,ab,kw OR **amphibian**:ti,ab,kw OR **amphibians**:ti,ab,kw OR **amphibia**:ti,ab,kw OR **frog**:ti,ab,kw OR **frogs**:ti,ab,kw OR **bombina**:ti,ab,kw OR **salientia**:ti,ab,kw OR **toad**:ti,ab,kw OR **toads**:ti,ab,kw OR **epidalea**:ti,ab,kw) AND **calamita**:ti,ab,kw OR **salamander**:ti,ab,kw OR **salamanders**:ti,ab,kw OR **fish**:ti,ab,kw OR **fishes**:ti,ab,kw OR **pisces**:ti,ab,kw OR **catfish**:ti,ab,kw OR **catfishes**:ti,ab,kw OR **siluriformes**:ti,ab,kw OR **arius**:ti,ab,kw OR **heteropneustes**:ti,ab,kw OR **sheatfish**:ti,ab,kw OR **perch**:ti,ab,kw OR **perches**:ti,ab,kw OR **percidae**:ti,ab,kw OR **perca**:ti,ab,kw OR **trout**:ti,ab,kw OR **trouts**:ti,ab,kw OR **char**:ti,ab,kw OR **chars**:ti,ab,kw OR **salvelinus**:ti,ab,kw OR **minnow**:ti,ab,kw OR **cyprinidae**:ti,ab,kw OR **carps**:ti,ab,kw OR **carp**:ti,ab,kw OR **zebrafish**:ti,ab,kw OR **zebrafishes**:ti,ab,kw OR **goldfish**:ti,ab,kw OR **goldfishes**:ti,ab,kw OR **guppy**:ti,ab,kw OR **guppies**:ti,ab,kw OR **chub**:ti,ab,kw OR **chubs**:ti,ab,kw OR **tinca**:ti,ab,kw OR **barbels**:ti,ab,kw OR **barbus**:ti,ab,kw OR **pimephales**:ti,ab,kw OR **promelas**:ti,ab,kw OR **'poecilia reticulata'**:ti,ab,kw OR **mullet**:ti,ab,kw OR **mullets**:ti,ab,kw OR **eel**:ti,ab,kw OR **eels**:ti,ab,kw OR **seahorse**:ti,ab,kw OR **seahorses**:ti,ab,kw OR **'mugil curema'**:ti,ab,kw OR **'atlantic cod'**:ti,ab,kw OR **shark**:ti,ab,kw OR **sharks**:ti,ab,kw OR **catshark**:ti,ab,kw OR **anguilla**:ti,ab,kw OR **salmonid**:ti,ab,kw OR **salmonids**:ti,ab,kw OR **whitefish**:ti,ab,kw OR **whitefishes**:ti,ab,kw OR **salmon**:ti,ab,kw OR **salmons**:ti,ab,kw OR **sole**:ti,ab,kw OR **solea**:ti,ab,kw OR **lamprey**:ti,ab,kw OR **lampreys**:ti,ab,kw OR **pumpkinseed**:ti,ab,kw OR **sunfish**:ti,ab,kw OR **sunfishes**:ti,ab,kw OR **tilapia**:ti,ab,kw OR **tilapias**:ti,ab,kw OR **turbot**:ti,ab,kw OR **turbots**:ti,ab,kw OR **flatfish**:ti,ab,kw OR **flatfishes**:ti,ab,kw OR **sciuridae**:ti,ab,kw OR **squirrel**:ti,ab,kw OR **squirrels**:ti,ab,kw OR **chipmunk**:ti,ab,kw OR **chipmunks**:ti,ab,kw OR **suslik**:ti,ab,kw OR **susliks**:ti,ab,kw OR **vole**:ti,ab,kw OR **voles**:ti,ab,kw OR **lemming**:ti,ab,kw OR **lemmings**:ti,ab,kw OR **muskrat**:ti,ab,kw OR **muskrats**:ti,ab,kw OR **lemmus**:ti,ab,kw OR **otter**:ti,ab,kw OR **otters**:ti,ab,kw OR **marten**:ti,ab,kw OR **martens**:ti,ab,kw OR **martes**:ti,ab,kw OR **weasel**:ti,ab,kw OR **badger**:ti,ab,kw OR **badgers**:ti,ab,kw OR **ermine**:ti,ab,kw OR **mink**:ti,ab,kw OR **minks**:ti,ab,kw OR **sable**:ti,ab,kw OR **sables**:ti,ab,kw OR **gulo**:ti,ab,kw OR **gulos**:ti,ab,kw OR **wolverine**:ti,ab,kw OR **wolverines**:ti,ab,kw OR **mustela**:ti,ab,kw OR **llama**:ti,ab,kw OR **llamas**:ti,ab,kw OR **alpaca**:ti,ab,kw OR **alpacas**:ti,ab,kw OR **camelid**:ti,ab,kw OR **camelids**:ti,ab,kw OR **guanaco**:ti,ab,kw OR **guanacos**:ti,ab,kw OR **chiroptera**:ti,ab,kw OR **chiropteras**:ti,ab,kw OR **bat**:ti,ab,kw OR **bats**:ti,ab,kw OR **fox**:ti,ab,kw OR **foxes**:ti,ab,kw OR **iguana**:ti,ab,kw OR **iguanas**:ti,ab,kw OR **'xenopus laevis'**:ti,ab,kw OR **parakeet**:ti,ab,kw OR **parakeets**:ti,ab,kw OR **parrot**:ti,ab,kw OR **parrots**:ti,ab,kw OR **donkey**:ti,ab,kw OR **donkeys**:ti,ab,kw OR **mule**:ti,ab,kw OR **mules**:ti,ab,kw OR **zebra**:ti,ab,kw OR **zebras**:ti,ab,kw OR **shrew**:ti,ab,kw OR **shrews**:ti,ab,kw OR **bison**:ti,ab,kw OR **bisons**:ti,ab,kw OR **buffalo**:ti,ab,kw OR **buffaloes**:ti,ab,kw OR **deer**:ti,ab,kw OR **deers**:ti,ab,kw OR **bear**:ti,ab,kw OR **bears**:ti,ab,kw OR **panda**:ti,ab,kw OR **pandas**:ti,ab,kw OR **'wild hog'**:ti,ab,kw OR **'wild boar'**:ti,ab,kw OR **fitchew**:ti,ab,kw OR **fitch**:ti,ab,kw OR **beaver**:ti,ab,kw OR **beavers**:ti,ab,kw OR **jerboa**:ti,ab,kw OR **jerboas**:ti,ab,kw OR **capybara**:ti,ab,kw OR **capybaras**:ti,ab,kw OR **canine**:ti,ab,kw OR **bovine**:ti,ab,kw OR **porcine**:ti,ab,kw OR **hog**:ti,ab,kw OR **hogs**:ti,ab,kw

w

3. Web of Science Core Collection

Interface: Clarivate Analytics<br><br>Editions = A&HCI , ESCI , SCI-EXPANDED , SSCI<br><br>Date of Search: 17 June 2022<br><br>Number of hits:<br><br>- Broad: 14104<br>- Narrow: 1243	Field labels<br><br>• TS/Topic = title, abstract, author keywords and Keywords Plus<br>• NEAR/x = within x words, regardless of order<br>• * = truncation of word for alternate endings<br><br>Note: the *Exact search*-function was used for all the searches

9
#8 AND #5
1,243

8
#7 OR #6
1,079,345

7
TS=((preclinical* or "pre-clinical*") and clinical*)
88,112

6
TS=(exploit* or translat*)
1,002,840

5
#4 AND #1
14,104

4
#3 OR #2
335,765

3
TI=("meta analy*" or metaanaly*)
190,277

2
TI=((systematic or scoping) and review)
232,253

1

TS=("animals" or "animal" or "mice" or "mus" or "mouse" or "murine" or "woodmouse" or "rats" or "rat" or "murinae" or "muridae" or "cottonrat" or "cottonrats" or "hamster" or "hamsters" or "cricetinae" or "rodentia" or "rodent" or "rodents" or "pigs" or "pig" or "swine" or "swines" or "piglets" or "piglet" or "boar" or "boars" or "sus scrofa" or "ferrets" or "ferret" or "polecat" or "polecats" or "mustela putorius" or "guinea pigs" or "guinea pig" or "cavia" or "callithrix" or "marmoset" or "marmosets" or "cebuella" or "hapale" or "octodon" or "chinchilla" or "chinchillas" or "gerbillinae" or "gerbil" or "gerbils" or "jird" or "jirds" or "merione" or "meriones" or "rabbits" or "rabbit" or "hares" or "hare" or "diptera" or "flies" or "fly" or "dipteral" or "drosophila" or "drosophilidae" or "cats" or "cat" or "carus" or "felis" or "nematoda" or "nematode" or "nematodes" or "sipunculida" or "dogs" or "dog" or "canine" or "canines" or "canis" or "sheep" or "sheeps" or "mouflon" or "mouflons" or "ovis" or "goats" or "goat" or "capra" or "capras" or "rupicapra" or "rupicapras" or "chamois" or "haplorhini" or "monkey" or "monkeys" or "anthropoidea" or "anthropoids" or "saguinus" or "tamarin" or "tamarins" or "leontopithecus" or "hominidae" or "ape" or "apes" or "panpaniscus" or "bonobo" or "bonobos" or "pan troglodytes" or "gibbon" or "gibbons" or "siamang" or "siamangs" or "nomascus" or "symphalangus" or "chimpanzee" or "chimpanzees" or "prosimian" or "prosimians" or "bush baby" or "bush babies" or "galagos" or "galago" or "pongidae" or "gorilla" or "gorillas" or "pongo pygmaeus" or "orangutan" or "orangutans" or "lemur" or "lemurs" or "lemuridae" or "horse" or "horses" or "equus" or "cow" or "calf" or "bull" or "chicken" or "chickens" or "gallus" or "quail" or "bird" or "birds" or "quails" or "poultry" or "poultries" or "fowl" or "fowls" or "reptile" or "reptilia" or "reptiles" or "snakes" or "snake" or "lizard" or "lizards" or "alligator" or "alligators" or "crocodile" or "crocodiles" or "turtle" or "turtles" or "amphibian" or "amphibians" or "amphibia" or "frog" or "frogs" or "bombina" or "salientia" or "toad" or "toads" or "epidalea calamita" or "salamander" or "salamanders" or "eel" or "eels" or "fish" or "fishes" or "pisces" or "catfish" or "catfishes" or "siluriformes" or "arius" or "heteropneustes" or "sheatfish" or "perch" or "perches" or "percidae" or "perca" or "trout" or "trouts" or "char" or "chars" or "salvelinus" or "minnow" or "cyprinidae" or "carps" or "carp" or "zebrafish" or "zebrafishes" or "goldfish" or "goldfishes" or "guppy" or "guppies" or "chub" or "chubs" or "tinca" or "barbels" or "barbus" or "pimephales" or "promelas" or "poecilia reticulata" or "mullet" or "mullets" or "eel" or "eels" or "seahorse" or "seahorses" or "mugil curema" or "atlantic cod" or "shark" or "sharks" or "catshark" or "anguilla" or "salmonid" or "salmonids" or "whitefish" or "whitefishes" or "salmon" or "salmons" or "sole" or "solea" or "lamprey" or "lampreys" or "pumpkinseed" or "sunfish" or "sunfishes" or "tilapia" or "tilapias" or "turbot" or "turbots" or "flatfish" or "flatfishes" or "sciuridae" or "squirrel" or "squirrels" or "chipmunk" or "chipmunks" or "suslik" or "susliks" or "vole" or "voles" or "lemming" or "lemmings" or "muskrat" or "muskrats" or "lemmus" or "otter" or "otters" or "marten" or "martens" or "martes" or "weasel" or "badger" or "badgers" or "ermine" or "mink" or "minks" or "sable" or "sables" or "gulo" or "gulos" or "wolverine" or "wolverines" or "mustela" or "llama" or "llamas" or "alpaca" or "alpacas" or "camelid" or "camelids" or "guanaco" or "guanacos" or "chiroptera" or "chiropteras" or "bat" or "bats" or "fox" or "foxes" or "iguana" or "iguanas" or "xenopus laevis" or "parakeet" or "parakeets" or "parrot" or "parrots" or "donkey" or "donkeys" or "mule" or "mules" or "zebra" or "zebras" or "shrew" or "shrews" or "bison" or "bisons" or "buffalo" or "buffaloes" or "deer" or "deers" or "bear" or "bears" or "panda" or "pandas" or "wild hog" or "wild boar" or "fitchew" or "fitch" or "beaver" or "beavers" or "jerboa" or "jerboas" or "capybara" or "capybaras"or "canine" or "bovine" or "porcine" or "hog" or "hogs")

Karolinska Institutet

7,444,252
