# Supplementary material for: Analysis of animal-to-human translation shows that only 5% of animal-tested therapeutic interventions obtain regulatory approval for human applications
Source: PLoS Biol. 2024 Jun 13;22(6):e3002667. doi: 10.1371/journal.pbio.3002667 (PMC11175415; doi:10.1371/journal.pbio.3002667)
Supplement: S2 Data — (DOCX) [file pbio.3002667.s002.docx]

**Critical appraisal of included systematic reviews**

This tool is based on a checklist proposed by Sena and colleagues [1], and extended with additional critical appraisal items. Each item is scored with a 0 = no (high risk of bias), a 1 = yes (low risk of bias), or a 2 = not reported (unclear risk of bias).

| **Item** | **Explanation** |
| --- | --- |
| 1) Was an a priori study protocol defined? | Checks whether the review mentions a predefined study protocol, typically found in the methods section. A simple mention is sufficient, without needing a direct link. |
| 2) Was a flowchart for study selection provided? | Checks for a flowchart detailing the number of studies identified, screened, and reasons for exclusions, aligning with PRISMA guidelines.This could be found in the main paper or any supplementary data. |
| 3) Was a conflict-of-interest statement provided? | Checks if a conflict of interest statement is present, focusing on the declaration rather than the existence of conflicts, usually at the manuscript's end. |
| 4) Was screening and/or extraction conducted by two or more reviewers? | Checks mention of two or more reviewers for data screening and extraction for either one or both steps, typically in the methods section. |
| 5) Was a clear research question defined? | Checks if the review clearly states its research question, aim, or objective, often in the abstract or introduction. |
| 6) Were in- and exclusion criteria reported? | Checks if the review outlines specific inclusion and exclusion criteria (e.g., that studies related to a certain topic are included or that studies in other languages than English are excluded), usually detailed in the methods section. |
| 7) Were two or more literature databases searched? | Checks whether at least two databases were searched for studies (e.g., PubMed, Scopus, or Embase), mostly described in the methods section. |
| 8) Was a search date provided? | Checks the provision of the search date, with a month and year being sufficient, typically in the methods section. |
| 9) Was a search string provided? | Checks if a search string is provided for the literature search (key words are sufficient), usually found in the methods section. |
| 10) Was a critical appraisal conducted? | Checks for critical appraisal of included studies using tools like CAMARADES or SYRCLE (or any other risk of bias assessment tool, including custom-made risk of bias assessment tools), typically mentioned in the methods section. |
| 11) Did the study mention alignment with relevant guidelines, e.g., SYRCLE, CAMARADES, or PRISMA? | Checks if the review mentions alignment with guidelines such as SYRCLE, CAMARADES, or PRISMA, usually in the methods section. |

**References**

1. Sena ES, Currie GL, McCann SK, Macleod MR, Howells DW. Systematic reviews and meta-analysis of preclinical studies: why perform them and how to appraise them critically. Journal of Cerebral Blood Flow & Metabolism. 2014;34(5):737-42.
