# Supplementary material for: Analysis of animal-to-human translation shows that only 5% of animal-tested therapeutic interventions obtain regulatory approval for human applications
Source: PLoS Biol. 2024 Jun 13;22(6):e3002667. doi: 10.1371/journal.pbio.3002667 (PMC11175415; doi:10.1371/journal.pbio.3002667)
Supplement: S1 Fig — (DOCX) [file pbio.3002667.s003.docx]

**Supplementary Figure 1**: Translational proportions for all therapies (neglecting development time).


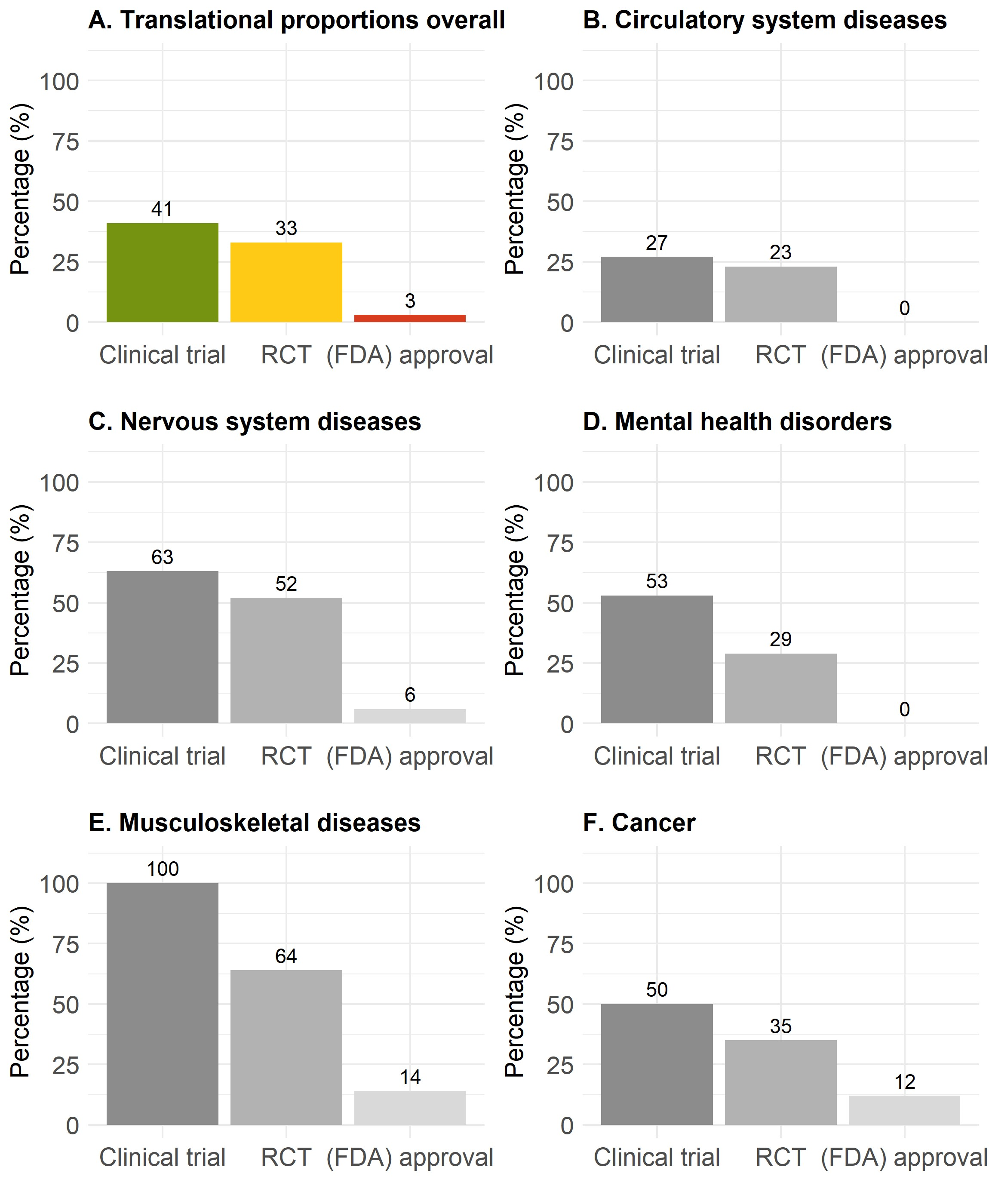


Proportions of translation from animals to any clinical study (green), to an RCT (yellow), or to (FDA) approval (red) overall (A), for circulatory system diseases (B), for neurological diseases (C), for mental health disorders (D), for musculoskeletal diseases (E), and for cancer (F).

The data underlying this figure can be found on <https://osf.io/frjm4> (Sheet: *Translation*). The code underlying this figure can be found on <https://osf.io/9fgru>.

*Abbreviations: RCT, randomized controlled trial.*
