# Supplementary material for: Analysis of animal-to-human translation shows that only 5% of animal-tested therapeutic interventions obtain regulatory approval for human applications
Source: PLoS Biol. 2024 Jun 13;22(6):e3002667. doi: 10.1371/journal.pbio.3002667 (PMC11175415; doi:10.1371/journal.pbio.3002667)
Supplement: S2 Fig — (DOCX) [file pbio.3002667.s004.docx]

**Supplementary Figure 2**: Lag times for clinical therapy development from first animal study only considering therapies which transitioned to a clinical trial.


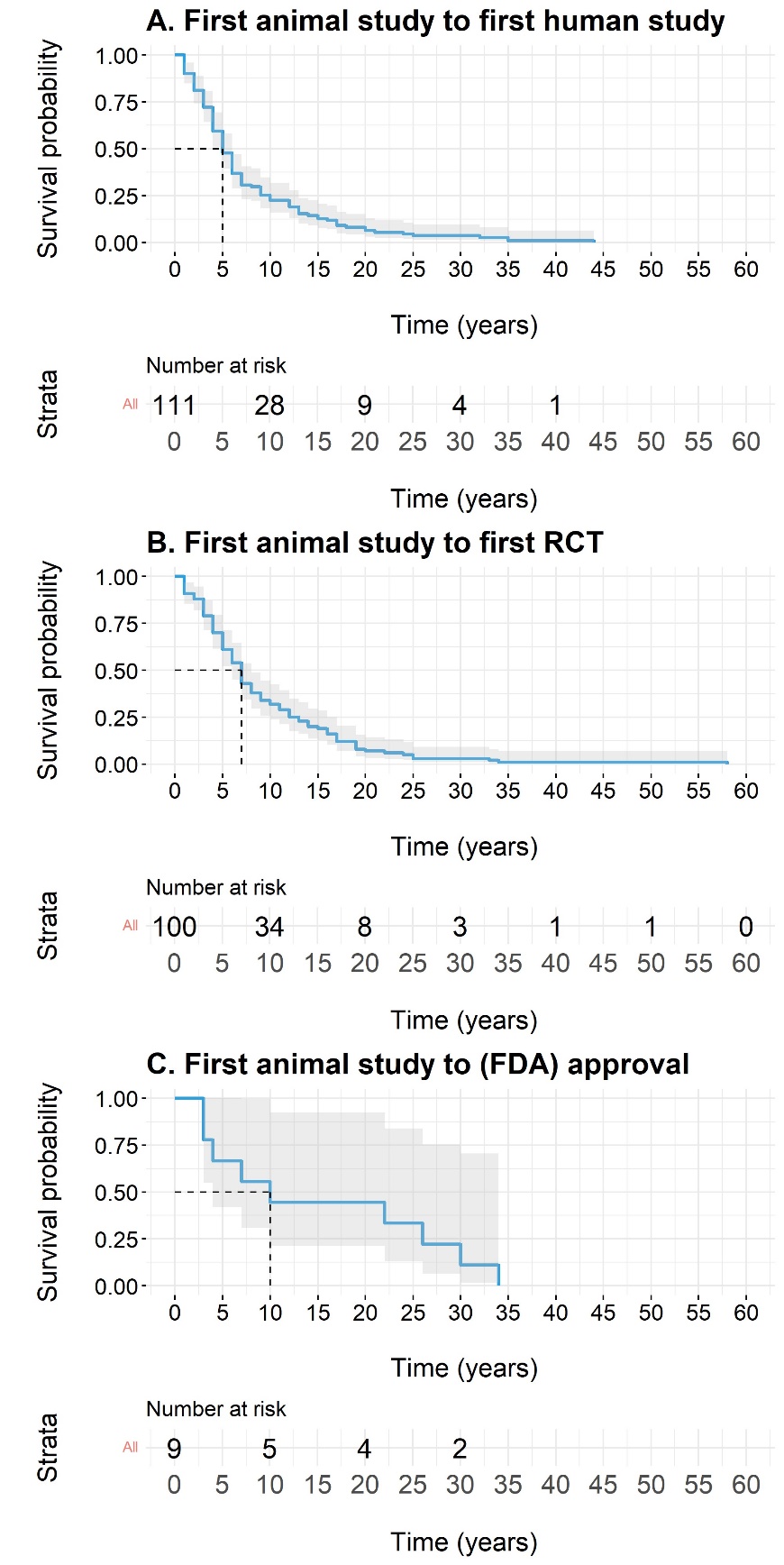


Lag times for therapies from first animal study to any clinical study (A), to a randomized controlled trial (RCT, B), or to (FDA) approval (C).

The data underlying this figure can be found on <https://osf.io/frjm4> (Sheet: *Translation*). The code underlying this figure can be found on <https://osf.io/9fgru>.

*Abbreviations: RCT, randomized controlled trial.*
