# Supplementary material for: Analysis of animal-to-human translation shows that only 5% of animal-tested therapeutic interventions obtain regulatory approval for human applications
Source: PLoS Biol. 2024 Jun 13;22(6):e3002667. doi: 10.1371/journal.pbio.3002667 (PMC11175415; doi:10.1371/journal.pbio.3002667)
Supplement: S6 Fig — (DOCX) [file pbio.3002667.s008.docx]

**Supplementary Figure 6**: Meta-analysis on concordance rate for musculoskeletal diseases (relative risk).


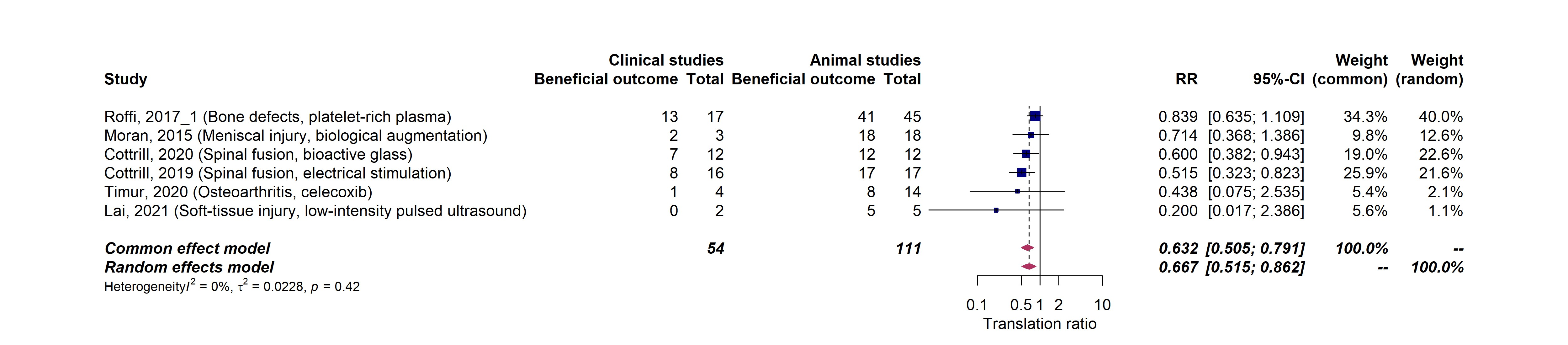


The data underlying this figure can be found on <https://osf.io/frjm4> (Sheet: *Mastersheet_including_RoB*). The code underlying this figure can be found on <https://osf.io/9fgru>.
