# Supplementary material for: Analysis of animal-to-human translation shows that only 5% of animal-tested therapeutic interventions obtain regulatory approval for human applications
Source: PLoS Biol. 2024 Jun 13;22(6):e3002667. doi: 10.1371/journal.pbio.3002667 (PMC11175415; doi:10.1371/journal.pbio.3002667)
Supplement: S1 Table — (DOCX) [file pbio.3002667.s011.docx]

**Supplementary Table 1**: Studies, interventions, and development status of included systematic reviews/therapeutic interventions.

| **Study, Title** | **Disease** | **Therapy** | **First animal study** | **Any human study** | **Year** | **Outcome** | **RCT** | **Year** | **Outcome** | **(FDA) approved** | **Year approval** |
| --- | --- | --- | --- | --- | --- | --- | --- | --- | --- | --- | --- |
| Abdulazeez, 2021: A systematic review with meta-analysis on the antihypertensive efficacy of Nigerian medicinal plants | Hypertension | M. cecropioides/stem bark | 2006 | No |  |  | No |  |  | No |  |
|  | Hypertension | B. coccineus/leaf | 2014 | No |  |  | No |  |  | No |  |
|  | Hypertension | Z. officinale.& C. longa/rhizome | 2016 | No |  |  | No |  |  | No |  |
|  | Hypertension | P. amarus/leaf | 2007 | No |  |  | No |  |  | No |  |
|  | Hypertension | P. americana/seed | 2009 | Yes | 2014 | Positive | No |  |  | No |  |
|  | Hypertension | H. sabdariffa/leaf | 2019 | No |  |  | No |  |  | No |  |
|  | Hypertension | V. album/leaf | 2004 | No |  |  | No |  |  | No |  |
|  | Hypertension | E. guineensis/oil | 2019 | No |  |  | No |  |  | No |  |
|  | Hypertension | P. Americana/seed | 2009 | No |  |  | No |  |  | No |  |
|  | Hypertension | M. flagellipes/seed | 2017 | No |  |  | No |  |  | No |  |
|  | Hypertension | V. doniana/stem bark | 1996 | No |  |  | No |  |  | No |  |
|  | Hypertension | H. sabdariffa/calyx | 2012 | No |  |  | No |  |  | No |  |
|  | Hypertension | E. camaldulensis/stem bark | 2018 | No |  |  | No |  |  | No |  |
|  | Hypertension | A. sativum/bulb | 2011 | No |  |  | No |  |  | No |  |
|  | Hypertension | N. latifolia/root | 2008 | No |  |  | No |  |  | No |  |
|  | Hypertension | L. bengwensis/leaf | 1996 | No |  |  | No |  |  | No |  |
|  | Hypertension | V. doniana/seed | 2001 | No |  |  | No |  |  | No |  |
|  | Hypertension | P. curatellifolia/bark | 2011 | No |  |  | No |  |  | No |  |
|  | Hypertension | V. amygdalina/leaf | 2018 | No |  |  | No |  |  | No |  |
|  | Hypertension | H. sabdariffa/calyx | 1999 | Yes | 2014 | Positive | No |  |  | No |  |
|  | Hypertension | A. sativum & Z. officinale/rizhome & bulb | 2015 | No |  |  | No |  |  | No |  |
| Alexander, 2018: Chemical burns: Diphoterine untangled | Chemical burns | Diphoterine (rinsing solution for burns) | 2002 | Yes | 2005 | Positive | Yes | 2002 | Positive | No |  |
| Alves, 2020: Prebiotic effect of dietary polyphenols: A systematic review | Nutrition | Dietary polyphenols (prebiotic): Flavonoids | 2015 | Yes | 2011 | Positive | Yes | 2011 | Unclear | No |  |
|  | Nutrition | Dietary polyphenols (prebiotic): Lignans - syringaresinol | 2016 | No |  |  | No |  |  | No |  |
|  | Nutrition | Dietary polyphenols (prebiotic): Phenolic acids - dicaffeoylquinic acids | 2019 | No |  |  | No |  |  | No |  |
|  | Nutrition | Dietary polyphenols (prebiotic): Stilbenes - pterostilbene | 2017 | No |  |  | No |  |  | No |  |
|  | Nutrition | Dietary polyphenols (prebiotic): Stilbenes - resveratrol | 2014 | No |  |  | No |  |  | No |  |
|  | Nutrition | Dietary polyphenols (prebiotic): Other polyphenols - vanillin | 2018 | No |  |  | No |  |  | No |  |
| Arunogiri, 2021: A systematic review of the effect of ovarian sex hormones on stimulant use in females | Addiction | Progesterone | 2003 | Yes | 2004 | Positive | Yes | 2014 | Positive | No |  |
|  | Addiction | Estrogen | 2001 | No |  |  | No |  |  | No |  |
| Austin, 2014: Aerobic exercise effects on neuroprotection and brain repair following stroke: a systematic review and perspective | Stroke | Aerobic exercise | 2002 | No |  |  | No |  |  | No |  |
| Baker, 2021: Therapeutic hypothermia for intracerebral hemorrhage: Systematic review and meta-analysis of the experimental and clinical literature | Intracerebral hemorrhage | Therapeutic hypothermia | 2001 | Yes | 2011 | Positive | No |  |  | No |  |
| Bhatti, 2017: Systematic Review of Human and Animal Studies Examining the Efficacy and Safety of N-Acetylcysteine (NAC) and N-Acetylcysteine Amide (NACA) in Traumatic Brain Injury: Impact on Neurofunctional Outcome and Biomarkers of Oxidative Stress and Inflammation | Traumatic brain injury | N-acetylcysteine | 1991 | Yes | 2011 | Unclear | Yes | 2013 | Positive | No |  |
|  | Traumatic brain injury | N-acetylcysteine amide | 2014 | No |  |  | No |  |  | No |  |
| Blais, 2021: Probiotic and commensal gut microbial therapies in multiple sclerosis and its animal models: a comprehensive review | Multiple sclerosis | Probiotics | 2006 | Yes | 2018 | Positive | Yes | 2017 | Positive | No |  |
| Brito, 2015: Enhanced analgesic activity by cyclodextrins - a systematic review and meta-analysis | Pain | Cyclodextrin complexes | 2009 | Yes | 2002 | Positive | Yes | 2002 | Positive | Yes | 2016 |
| Cadotte, 2011: Spinal cord injury: a systematic review of current treatment options | Spinal cord injury | Riluzole | 1996 | Yes | 2000 | Positive | Yes | 2021 | Negative | No |  |
|  | Spinal cord injury | Cethrin | 2002 | Yes | 2006 | Positive | Yes | 2021 | Negative | No |  |
|  | Spinal cord injury | Early surgical decompression | NA | Yes | 1995 | Positive | Yes | 2018 | Unclear | No |  |
| Carvalho, 2022: Citrus Extract as a Perspective for the Control of Dyslipidemia: A Systematic Review With Meta-Analysis From Animal Models to Human Studies | Dyslipidemia | Citrus extract | 1998 | Yes | 2007 | Positive | Yes | 2017 | Positive | No |  |
| Cavallo, 2021: Bone marrow concentrate injections for the treatment of osteoarthritis: evidence from preclinical findings to the clinical application | Osteoarthritis | Bone marrow concentrate injections | 2014 | Yes | 2014 | Positive | Yes | 2018 | Positive | No |  |
| Cecoro, 2022: Effects of Magnetic Stimulation on Dental Implant Osseointegration: A Scoping Review | Dental Implant Osseointegration | Magnetic stimulation | 1996 | Yes | 2016 | Positive | Yes | 2012 | Positive | No |  |
| Cossu, 2015: The Role of Mifepristone in Meningiomas Management: A Systematic Review of the Literature | Meningioma | Mifepristone | 1987 | Yes | 1991 | Positive | Yes | 2001 | Negative | No |  |
| Cottrill, 2019: The effect of electrical stimulation therapies on spinal fusion: a cross-disciplinary systematic review and meta-analysis of the preclinical and clinical data | Spinal fusion surgery | Electrical stimulation | 1986 | Yes | 1984 | Positive | Yes | 1988 | Positive | Yes | 2020 |
| Cottrill, 2020: The effect of bioactive glasses on spinal fusion: A cross-disciplinary systematic review and meta-analysis of the preclinical and clinical data | Spinal fusion surgery | Bioactive glass | 2000 | Yes | 1992 | Positive | Yes | 2003 | Positive | Yes | 2022 |
| Creta, 2021: Inhibition of androgen signalling improves the outcomes of therapies for bladder cancer: Results from a systematic review of preclinical and clinical evidence and meta-analysis of clinical studies | Bladder Cancer | Flutamide | 2012 | No |  |  | No |  |  | No |  |
|  | Bladder Cancer | ASC-J9 | 2015 | No |  |  | No |  |  | No |  |
|  | Bladder Cancer | Bicalutamide | 2012 | No |  |  | No |  |  | No |  |
|  | Bladder Cancer | Enzalutamide | 2017 | No |  |  | No |  |  | No |  |
|  | Bladder Cancer | Synthetic androgen R1881 | 2016 | No |  |  | No |  |  | No |  |
| Daldegan, 2021_1: Co-exposure of cannabinoids with amphetamines and biological, behavioural and health outcomes: a scoping review of animal and human studies | Addiction | Cannabinoids/Cannabis | 2001 | Yes | 2004 | Positive | No |  |  | No |  |
| Daldegan, 2021_2: Co-exposure of cocaine and cannabinoids and its association with select biological, behavioural and health outcomes: A systematic scoping review of multi-disciplinary studies | Addiction | Cannabinoids/Cannabis | 2001 | Yes | 2002 | Positive | Yes | 2020 | Neutral | No |  |
| DePhillipo, 2018: Efficacy of Vitamin C Supplementation on Collagen Synthesis and Oxidative Stress After Musculoskeletal Injuries: A Systematic Review | Musculoskeletal Injuries | Vitamin C | 2001 | Yes | 2014 | Neutral | Yes | 2009 | Positive | No |  |
| Durg, 2020: Withania somnifera (Indian ginseng) in diabetes mellitus: A systematic review and meta-analysis of scientific evidence from experimental research to clinical application | Diabetes | Indian ginseng | 2004 | Yes | 2000 | Positive | Yes | 2013 | Positive | No |  |
| Franzetti, 2022: Stereotactic Radiotherapy Ablation and Atrial Fibrillation: Technical Issues and Clinical Expectations Derived From a Systematic Review | Atrial Fibrillation | Stereotactic radiotherapy ablation | 2010 | Yes | 2016 | Neutral | No |  |  | No |  |
| Gho, 2013: Cell therapy, a novel remedy for dilated cardiomyopathy? A systematic review | Cardiomyopathy | Cell therapy | 2001 | Yes | 2007 | Positive | Yes | Yes | Positive | No |  |
| Glass, 2017: Does negative-pressure wound therapy influence subjacent bacterial growth? A systematic review | Wound therapy | Negative-pressure therapy | 1997 | Yes | 2003 | Positive | Yes | 2004 | Positive | No |  |
| Ghanbari, 2021: Effect of Microalgae Arthrospira on Biomarkers of Glycemic Control and Glucose Metabolism: A Systematic Review and Meta-analysis | Glucose Metabolism | Spirulina | 2001 | Yes | 1982 | Positive | Yes | 1982 | Positive | No |  |
| Goldberg, 2017: The use of mesenchymal stem cells for cartilage repair and regeneration: a systematic review | Cartilage defects | Mesenchymal stem cells | 1995 | Yes | 2007 | Positive | Yes | 2003 | Positive | No |  |
| Gruenbaum, 2019: Branched-Chain Amino Acids and Seizures: A Systematic Review of the Literature | Epilepsy | Branched-chain amino acids | 1974 | Yes | 2009 | Unclear | No |  |  | No |  |
| Gundestrup, 2020: Mesenchymal Stem Cell Therapy for Osteoradionecrosis of the Mandible: a Systematic Review of Preclinical and Human Studies | Head and neck cancer, Radiotherapy | Mesenchymal stem cells | 2012 | Yes | 2010 | Positive | No |  |  | No |  |
| Guo, 2021: Shexiang Baoxin Pill for Acute Myocardial Infarction: Clinical Evidence and Molecular Mechanism of Antioxidative Stress | Acute myocaridal infarction | Shexiang Baoxin Pill | 1999 | Yes | 2015 | Positive | Yes | 2015 | Positive | No |  |
| Hansen, 2021: Translational challenges of remote ischemic conditioning in ischemic stroke - a systematic review | Stroke | Remote ischemic conditioning | 2011 | Yes | 2014 | Positive | Yes | 2014 | Positive | No |  |
| He, 2019: Bioartificial liver support systems for acute liver failure: A systematic review and meta-analysis of the clinical and preclinical literature | Liver failure | Bioartificial liver | 2011 | Yes | 1994 | Positive | Yes | 2004 | Neutral | No |  |
| Heard, 2018: Repurposing Proteostasis-Modifying Drugs to Prevent or Treat Age-Related Dementia: A Systematic Review | Dementia | Lithium | 2007 | Yes | 1983 | Negative | Yes | 2009 | Positive | No |  |
|  | Dementia | Rapamycin | 2010 | Yes | 2017 | Neutral | No |  |  | No |  |
|  | Dementia | Rifampicin | 2016 | Yes | 2013 | Neutral | No |  |  | No |  |
|  | Dementia | Minocycline | 2007 | Yes | 2013 | Neutral | No |  |  | No |  |
|  | Dementia | Bosutinib | 2013 | No |  |  | No |  |  | No |  |
|  | Dementia | M-CSF | 2009 | No |  |  | No |  |  | No |  |
|  | Dementia | GM-CSF | 2010 | No |  |  | No |  |  | No |  |
|  | Dementia | Methylene blue | 2008 | No |  |  | No |  |  | No |  |
|  | Dementia | Geranylgeranylacetone | 2013 | No |  |  | No |  |  | No |  |
|  | Dementia | Dantrolene | 2012 | No |  |  | No |  |  | No |  |
|  | Dementia | Phenylbutyric acid | 2011 | No |  |  | No |  |  | No |  |
| Heinzel, 2021: A systematic review and meta-analysis of studies comparing muscle-in-vein conduits with autologous nerve grafts for nerve reconstruction | segmental nerve defects | Muscle-in-vein graft | 1993 | Yes | 2014 | Neutral | No |  |  | No |  |
| Henriksen, 2020: Systematic Review of Stem-Cell-Based Therapy of Burn Wounds: Lessons Learned from Animal and Clinical Studies | Burn wounds | Stem cells | 2012 | Yes | 2012 | Positive | No |  |  | No |  |
| Hexter, 2018: Biological augmentation of graft healing in anterior cruciate ligament reconstruction: a systematic review | Injury to the anterior cruciate ligament | Biological augmentatio | 2001 | Yes | 2003 | Positive | Yes | 2004 | Positive | No |  |
| Hooijmans, 2019: Remyelination promoting therapies in multiple sclerosis animal models: a systematic review and meta-analysis | Multiple sclerosis | 4-hydroxyquinazolin | 2010 | No |  |  | No |  |  | No |  |
|  | Multiple sclerosis | AH6809 (EP2 antagonist) | 2013 | No |  |  | No |  |  | No |  |
|  | Multiple sclerosis | AL-8810 (Prostaglandin F2 alpha analog) | 2014 | No |  |  | No |  |  | No |  |
|  | Multiple sclerosis | Amphotericin B | 2015 | No |  |  | No |  |  | No |  |
|  | Multiple sclerosis | Anti-Lingo-1 | 2009 | Yes | 2013 | Neutral | Yes | 2013 | Neutral | No |  |
|  | Multiple sclerosis | Apotransferrin | 2006 | No |  |  | No |  |  | No |  |
|  | Multiple sclerosis | Areca catechu nut extract | 2015 | No |  |  | No |  |  | No |  |
|  | Multiple sclerosis | Benztropine | 2015 | No |  |  | No |  |  | No |  |
|  | Multiple sclerosis | Bucladesine | 2015 | No |  |  | No |  |  | No |  |
|  | Multiple sclerosis | CCX771 (CXCR7 antagonist) | 2014 | No |  |  | No |  |  | No |  |
|  | Multiple sclerosis | Celecoxib | 2013 | No |  |  | No |  |  | No |  |
|  | Multiple sclerosis | 17Beta-estradiol | 2012 | Yes | 2021 | Neutral | Yes | 2021 | Neutral | No |  |
|  | Multiple sclerosis | Cyclicphosphatidic acid | 2014 | No |  |  | No |  |  | No |  |
|  | Multiple sclerosis | Cyclosporin | 2001 | Yes | 1990 | Positive | Yes | 1990 | Positive | No |  |
|  | Multiple sclerosis | CDP choline | 2015 | No |  |  | No |  |  | No |  |
|  | Multiple sclerosis | Dizocilipine | 2013 | No |  |  | No |  |  | No |  |
|  | Multiple sclerosis | Dl-3-n-butylphthalide | 2015 | No |  |  | No |  |  | No |  |
|  | Multiple sclerosis | Ebselen | 2009 | Yes | 2004 | Unclear | Yes | 2004 | Unclear | No |  |
|  | Multiple sclerosis | Electromagnetic field stimulation (EMFs) | 2010 | Yes | 2003 | Positive | Yes | 2003 | Positive | No |  |
|  | Multiple sclerosis | Epidermal growth factor-like | 2009 | No |  |  | No |  |  | No |  |
|  | Multiple sclerosis | Epimedium flavonoids | 2015 | No |  |  | No |  |  | No |  |
|  | Multiple sclerosis | Estrogen receptor agonist G1 | 2013 | No |  |  | No |  |  | No |  |
|  | Multiple sclerosis | Fingolimod | 2011 | Yes | 2006 | Positive | Yes | 2006 | Positive | Yes | 2010 |
|  | Multiple sclerosis | Dimethyl Fumaric acid ester | 2010 | Yes | 2008 | Positive | Yes | 2008 | Positive | Yes | 2013 |
|  | Multiple sclerosis | Geissoschizine methyl ether | 2013 | No |  |  | No |  |  | No |  |
|  | Multiple sclerosis | Glatiramer acetate (GA) | 2009 | Yes | 1995 | Positive | Yes | 1995 | Positive | Yes | 1996 |
|  | Multiple sclerosis | Iloprost | 2013 | No |  |  | No |  |  | No |  |
|  | Multiple sclerosis | Indazol chloride | 2013 | No |  |  | No |  |  | No |  |
|  | Multiple sclerosis | Lactacystin | 2009 | No |  |  | No |  |  | No |  |
|  | Multiple sclerosis | Laquinimod | 2011 | Yes | 2014 | Neutral | Yes | 2014 | Neutral | No |  |
|  | Multiple sclerosis | Latanoprost | 2014 | No |  |  | No |  |  | No |  |
|  | Multiple sclerosis | Leukemia inhibiting factor | 2008 | No |  |  | No |  |  | No |  |
|  | Multiple sclerosis | Macrophage colony-stimulating factor | 2015 | No |  |  | No |  |  | No |  |
|  | Multiple sclerosis | Melatonin | 2014 | Yes | 2013 | Positive | Yes | 2017 | Neutral | No |  |
|  | Multiple sclerosis | Methotrexat | 2013 | Yes | 1992 | Positive | Yes | 1992 | Positive | Yes | 1999 |
|  | Multiple sclerosis | Methylprednisolone | 1998 | Yes | 1983 | Positive | Yes | 1983 | Positive | Yes | 1957 |
|  | Multiple sclerosis | Mexiletine | 2010 | Yes | 1991 | Positive | No |  |  | No |  |
|  | Multiple sclerosis | Minocycline | 2005 | Yes | 2009 | Neutral | Yes | 2009 | Neutral | No |  |
|  | Multiple sclerosis | MK886 | 2012 | No |  |  | No |  |  | No |  |
|  | Multiple sclerosis | Monocloncal anti-SCH94.03 IgM-kappa | 1998 | No |  |  | No |  |  | No |  |
|  | Multiple sclerosis | N6-cyclohexyladenosine | 2013 | No |  |  | No |  |  | No |  |
|  | Multiple sclerosis | Neurotrophin 3 | 2003 | No |  |  | No |  |  | No |  |
|  | Multiple sclerosis | Ninjin’yoeito | 2007 | No |  |  | No |  |  | No |  |
|  | Multiple sclerosis | Noggin | 2011 | No |  |  | No |  |  | No |  |
|  | Multiple sclerosis | Olesoxime | 2012 | Yes | 2016 | Unclear | No |  |  | No |  |
|  | Multiple sclerosis | Omega-3 poly unsatturated fatty acid (PUFA) diet | 2009 | Yes | 2005 | Positive | Yes | 2005 | Positive | No |  |
|  | Multiple sclerosis | Plateled derived growth factor | 2001 | No |  |  | No |  |  | No |  |
|  | Multiple sclerosis | Polyclonal IgG | 1998 | No |  |  | No |  |  | No |  |
|  | Multiple sclerosis | Progesterone | 2004 | Yes | 2021 | Neutral | Yes | 2021 | Neutral | No |  |
|  | Multiple sclerosis | Quetiapine | 2008 | Yes | 2014 | Unclear | Yes | 2014 | Unclear | No |  |
|  | Multiple sclerosis | Recombinant human Gas6 (rhGas6) | 2010 | Yes | 2002 | Unclear | Yes | 2002 | Unclear | No |  |
|  | Multiple sclerosis | RhGGF-2 | 2003 | No |  |  | No |  |  | No |  |
|  | Multiple sclerosis | Scutellarin | 2015 | No |  |  | No |  |  | No |  |
|  | Multiple sclerosis | Serum derived human polyclonal IgM (sHIgM) | 2002 | No |  |  | No |  |  | No |  |
|  | Multiple sclerosis | Serum-derived human monoclonal IgM14 (sHIgM14) | 2002 | No |  |  | No |  |  | No |  |
|  | Multiple sclerosis | Serum-derived human monoclonal IgM22 (sHIgM22) | 2002 | Yes | 2015 | Unclear | Yes | 2015 | Unclear | No |  |
|  | Multiple sclerosis | Serum-derived human polyclonal IgG (sHIgG) | 2002 | No |  |  | No |  |  | No |  |
|  | Multiple sclerosis | Sildenafil | 2012 | Yes | 2009 | Neutral | Yes | 2009 | Neutral | No |  |
|  | Multiple sclerosis | Simvastatin | 2008 | Yes | 2010 | Positive | Yes | 2010 | Positive | No |  |
|  | Multiple sclerosis | Thymosin beta4 | 2016 | No |  |  | No |  |  | No |  |
|  | Multiple sclerosis | Trapidil | 1997 | No |  |  | No |  |  | No |  |
|  | Multiple sclerosis | Vitamin D (Cholecalciferol) | 2009 | Yes | 2007 | Positive | Yes | 2007 | Positive | No |  |
|  | Multiple sclerosis | Vitamin E | 2009 | Yes | 2014 | Positive | Yes | 2021 | Positive | No |  |
|  | Multiple sclerosis | Vitamin E derivate (Tocopherol derivate TFA-12) | 2013 | No |  |  | No |  |  | No |  |
|  | Multiple sclerosis | Monomethyl Fumaric acid ester | 2010 | Yes | 2017 | Positive | Yes | 2017 | Positive | Yes | 2020 |
|  | Multiple sclerosis | Cloprostenol | 2014 | No |  |  | No |  |  | No |  |
|  | Multiple sclerosis | Travoprost | 2014 | No |  |  | No |  |  | No |  |
|  | Multiple sclerosis | Androstendiol | 2015 | No |  |  | No |  |  | No |  |
|  | Multiple sclerosis | Electro acupuncture | 2011 | Yes | 2012 | Positive | Yes | 2012 | Positive | No |  |
|  | Multiple sclerosis | Erythropoietin | 2012 | Yes | 2017 | Neutral | Yes | 2017 | Neutral | No |  |
|  | Multiple sclerosis | Valproic acid | 2008 | Yes | 2003 | Positive | Yes | 2014 | Unclear | No |  |
|  | Multiple sclerosis | Quetiapine | 2008 | No |  |  | No |  |  | No |  |
|  | Multiple sclerosis | Triiodothyronine | 2012 | Yes | 2016 | Positive | No |  |  | No |  |
| Hwang, 2021: Toxic Animal-Based Medicinal Materials Can Be Effective in Treating Endometriosis: A Scoping Review | Endometriosis | Toxic animal-based medicinal materials | 2005 | Yes | 1993 | Positive | Yes | 2009 | Positive | No |  |
| Jensen, 2014: 4-Aminopyridine for symptomatic treatment of multiple sclerosis: a systematic review | Multiple sclerosis | 4-Aminopyridine | 1980 | Yes | 1983 | Positive | Yes | 1983 | Positive | Yes | 2010 |
| Khorshidi, 2021: Resveratrol: A "miracle" drug in neuropsychiatry or a cognitive enhancer for mice only? A systematic review and meta-analysis | Cognitive decline | Resveratrol | 2002 | Yes | 2012 | Positive | Yes | 2012 | Positive | No |  |
| Lalu, 2019: Mapping the preclinical to clinical evidence and development trajectory of the oncolytic virus talimogene laherparepvec (T-VEC): a systematic review | Cancer, oncolytic therapy | Oncolytic virus talimogene laherparepvec | 2003 | Yes | 2006 | Positive | Yes | 2010 | Positive | No |  |
| Lai, 2021: Low-Intensity Pulsed Ultrasound Augments Tendon, Ligament, and Bone-Soft Tissue Healing in Preclinical Animal Models: A Systematic Review | Soft-tissue injury | Low-intensity pulsed ultrasound | 1982 | Yes | 2006 | Neutral | Yes | 2006 | Neutral | No |  |
| Lalu, 2020: From the Lab to Patients: a Systematic Review and Meta-Analysis of Mesenchymal Stem Cell Therapy for Stroke | Stroke | Mesenchymal stem cells | 2000 | Yes | 2009 | Neutral | Yes | 2009 | Neutral | No |  |
| Lambert, 2020: Improving Newborn Respiratory Outcomes With a Sustained Inflation: A Systematic Narrative Review of Factors Regulating Outcome in Animal and Clinical Studies | Respiratory outcomes in neonates | Sustained inflation | 2009 | Yes | 2005 | Positive | Yes | 2005 | Positive | No |  |
| Leta, 2021: Neurogenic and anti-inflammatory effects of probiotics in Parkinson's disease: A systematic review of preclinical and clinical evidence | Parkinsons disease | Probiotics | 2019 | Yes | 2011 | Positive | Yes | 2016 | Positive | No |  |
| Li, 2018: The non-reconstructive treatment of complete ACL tear with biological enhancement in clinical and preclinical studies: A systematic review | ACL tear | Biological enhancement | 2007 | Yes | 2002 | Neutral | No |  |  | No |  |
| Lilamand, 2020: Are ketogenic diets promising for Alzheimer's disease? A translational review | Alzheimer's disease | Ketogenic diet | 2005 | Yes | 2004 | Positive | Yes | 2004 | Positive | No |  |
| Linde, 2021: Efficacy of topical cannabinoids in the management of pain: a systematic review and meta-analysis of animal studies | Pain, cannabis | Cannabinoids | 1996 | Yes | 2003 | Positive | Yes | 2003 | Positive | No |  |
| Liu, 2021: Sphingosine-1-Phosphate and Its Signal Modulators Alleviate Psoriasis-Like Dermatitis: Preclinical and Clinical Evidence and Possible Mechanisms | Psoriasis | Fingolimod | 2013 | No |  |  | No |  |  | No |  |
|  | Psoriasis | SYL930 | 2018 | No |  |  | No |  |  | No |  |
|  | Psoriasis | IMMH002 | 2020 | No |  |  | No |  |  | No |  |
|  | Psoriasis | HWG-35D | NA | No |  |  | No |  |  | No |  |
| Liu, 2020: Investigation of S-Nitrosoglutathione in stroke: A systematic review and meta-analysis of literature in pre-clinical and clinical research | Stroke | S-Nitrosoglutathione | 2005 | Yes | 1998 | Positive | No |  |  | No |  |
| Lind, 2021: Translation from animal studies of novel pharmacological therapies to clinical trials in cardiac arrest: A systematic review | Cardiac arrest | Argon | 2013 | No |  |  | No |  |  | No |  |
|  | Cardiac arrest | Carbon monoxide | 2002 | No |  |  | No |  |  | No |  |
|  | Cardiac arrest | Dexmedetomidine | 2006 | No |  |  | No |  |  | No |  |
|  | Cardiac arrest | Helium | 2016 | No |  |  | No |  |  | No |  |
|  | Cardiac arrest | Hydrogen | 2000 | Yes | 2017 | Unclear | Yes | 2016 | Unclear | No |  |
|  | Cardiac arrest | Hydrogen sulfide | 2007 | No |  |  | No |  |  | No |  |
|  | Cardiac arrest | Isoflurane | 2000 | No |  |  | No |  |  | No |  |
|  | Cardiac arrest | Pentazocine | 2003 | No |  |  | No |  |  | No |  |
|  | Cardiac arrest | Propofol | 2004 | No |  |  | No |  |  | No |  |
|  | Cardiac arrest | Sevoflurane | 2000 | No |  |  | No |  |  | No |  |
|  | Cardiac arrest | Sodium hydrogen sulfide (NaHS) | 2009 | No |  |  | No |  |  | No |  |
|  | Cardiac arrest | Sodium sulfide (Na2S) | 2012 | No |  |  | No |  |  | No |  |
|  | Cardiac arrest | Thiopental | 2000 | Yes | 1986 | Neutral | Yes | 1986 | Neutral | No |  |
|  | Cardiac arrest | Xenon | 2008 | Yes | 2013 | Neutral | Yes | 2016 | Positive | No |  |
|  | Cardiac arrest | ATP sensitive potassium channel inhibition HMR 1883 | 2000 | No |  |  | No |  |  | No |  |
|  | Cardiac arrest | Bretylium | 2000 | Yes | 1981 | Neutral | Yes | 1981 | Neutral | No |  |
|  | Cardiac arrest | Calcium | 2002 | Yes | 1984 | Neutral | No |  |  | No |  |
|  | Cardiac arrest | Dantrolene | 2014 | No |  |  | No |  |  | No |  |
|  | Cardiac arrest | Dronedarone | 2007 | No |  |  | No |  |  | No |  |
|  | Cardiac arrest | Ivabradine | 2008 | No |  |  | No |  |  | No |  |
|  | Cardiac arrest | Nifekalant | 2005 | Yes | 2006 | Positive | No |  |  | No |  |
|  | Cardiac arrest | Potassium chloride | 2001 | No |  |  | No |  |  | No |  |
|  | Cardiac arrest | Nitric oxide | 2000 | Yes | 1984 | Neutral | No |  |  | No |  |
|  | Cardiac arrest | Anti-TNF Etanercept or Infliximab | 2013 | No |  |  | No |  |  | No |  |
|  | Cardiac arrest | HMGB1 antibody | 2016 | No |  |  | No |  |  | No |  |
|  | Cardiac arrest | HMGB1 binding heptamer peptide | 2017 | No |  |  | No |  |  | No |  |
|  | Cardiac arrest | Melanocortinanalog NDP-α-MSH | 2001 | No |  |  | No |  |  | No |  |
|  | Cardiac arrest | Minocycline (tetracyclin) | 2010 | No |  |  | No |  |  | No |  |
|  | Cardiac arrest | Pravastatin or Pravastatin+Simvastatin | 2003 | No |  |  | No |  |  | No |  |
|  | Cardiac arrest | Protease activated receptor 2 inhibitor FSLLRY-NH2 | 2020 | No |  |  | No |  |  | No |  |
|  | Cardiac arrest | Rapamycin | 2010 | No |  |  | No |  |  | No |  |
|  | Cardiac arrest | Resolvin D1 | 2014 | No |  |  | No |  |  | No |  |
|  | Cardiac arrest | Selective serotonin reuptake inhibitor Fluoxetine | 2012 | No |  |  | No |  |  | No |  |
|  | Cardiac arrest | TLR2-inhibiting antibody | 2013 | No |  |  | No |  |  | No |  |
|  | Cardiac arrest | TLR4 antagonist Naltrexone | 2003 | No |  |  | No |  |  | No |  |
|  | Cardiac arrest | Ulinastatin trypsin inhibitor | 2004 | No |  |  | No |  |  | No |  |
|  | Cardiac arrest | Valproic acid | 2013 | No |  |  | No |  |  | No |  |
|  | Cardiac arrest | Edaravone | 2009 | No |  |  | No |  |  | No |  |
|  | Cardiac arrest | Sodium 2-sulfophenyl-N-tert-butyl nitrone | 2003 | No |  |  | No |  |  | No |  |
|  | Cardiac arrest | Tempol | 2002 | No |  |  | No |  |  | No |  |
|  | Cardiac arrest | U-74389G Lazaroid agent | 2001 | No |  |  | No |  |  | No |  |
|  | Cardiac arrest | Sodium bicarbonate | 2000 | Yes | 2006 | Positive | Yes | 2017 | Neutral | No |  |
|  | Cardiac arrest | Tris buffer mixture | 2002 | No |  |  | No |  |  | No |  |
|  | Cardiac arrest | Abciximab, GPIIb/IIIa inhibitor | 2010 | No |  |  | No |  |  | No |  |
|  | Cardiac arrest | Antithrombin | 2003 | No |  |  | No |  |  | No |  |
|  | Cardiac arrest | Eptifibatide GPIIb/IIIa inhibitor | 2005 | No |  |  | No |  |  | No |  |
|  | Cardiac arrest | Recombinant human activated protein C (rhAPC) | 2008 | No |  |  | No |  |  | No |  |
|  | Cardiac arrest | Streptokinase | 2002 | No |  |  | No |  |  | No |  |
|  | Cardiac arrest | Urokinase | 2000 | No |  |  | No |  |  | No |  |
|  | Cardiac arrest | Adenosine + lidocaine | 2004 | No |  |  | No |  |  | No |  |
|  | Cardiac arrest | NHE-1 inhibitor Zoniporide and CO2 consuming buffer | 2007 | No |  |  | No |  |  | No |  |
|  | Cardiac arrest | 4-phenylchalcone oxide (4-PCO) (epoxide hydrolase inhibitor) | 2013 | No |  |  | No |  |  | No |  |
|  | Cardiac arrest | Soluble epoxide hydrolase inhibitor AS2586144-CL | 2008 | No |  |  | No |  |  | No |  |
|  | Cardiac arrest | Anisodamine | 2011 | No |  |  | No |  |  | No |  |
|  | Cardiac arrest | Dl-3-n-Butylphthalide | 2016 | No |  |  | No |  |  | No |  |
|  | Cardiac arrest | Embelin | 2015 | No |  |  | No |  |  | No |  |
|  | Cardiac arrest | Epigallocatechin gallate | 2019 | No |  |  | No |  |  | No |  |
|  | Cardiac arrest | Gynostemma pentaphyllum | 2017 | No |  |  | No |  |  | No |  |
|  | Cardiac arrest | Oxymatrine | 2018 | No |  |  | No |  |  | No |  |
|  | Cardiac arrest | Resveratrol | 2000 | No |  |  | No |  |  | No |  |
|  | Cardiac arrest | Shen Fu | 2016 | Yes | 2017 | Positive | Yes | 2017 | Positive | No |  |
|  | Cardiac arrest | β-sodium aescinate | 2013 | No |  |  | No |  |  | No |  |
|  | Cardiac arrest | Erythropoeitin | 2013 | Yes | 2016 | Neutral | Yes | 2016 | Neutral | No |  |
|  | Cardiac arrest | Estradiol | 2000 | No |  |  | No |  |  | No |  |
|  | Cardiac arrest | Ghrelin | 2015 | No |  |  | No |  |  | No |  |
|  | Cardiac arrest | Glucagon | 2010 | No |  |  | No |  |  | No |  |
|  | Cardiac arrest | Glucagon-like-peptide-1 | 2010 | Yes | 2016 | Neutral | Yes | 2016 | Neutral | No |  |
|  | Cardiac arrest | Granulocyte colony-stimulating factor (G-CSF) | 2006 | No |  |  | No |  |  | No |  |
|  | Cardiac arrest | Insulin-like growth factor 1 | 2002 | No |  |  | No |  |  | No |  |
|  | Cardiac arrest | Melatonin | 2000 | No |  |  | No |  |  | No |  |
|  | Cardiac arrest | Fructose-1,6-bisphosphate | 2001 | No |  |  | No |  |  | No |  |
|  | Cardiac arrest | Glibenclamide | 2012 | No |  |  | No |  |  | No |  |
|  | Cardiac arrest | Phosphocreatine | 2000 | No |  |  | No |  |  | No |  |
|  | Cardiac arrest | Pyruvate | 2001 | No |  |  | No |  |  | No |  |
|  | Cardiac arrest | Ringer’s ethyl pyruvate solution | 2009 | No |  |  | No |  |  | No |  |
|  | Cardiac arrest | AP39 / H2S donor | 2013 | No |  |  | No |  |  | No |  |
|  | Cardiac arrest | Cyclosporine | 2013 | Yes | 2016 | Neutral | Yes | 2016 | Neutral | No |  |
|  | Cardiac arrest | Dynamin-related protein 1 inhibitor Mdivi-1 | 2014 | No |  |  | No |  |  | No |  |
|  | Cardiac arrest | NIM811 (cyclophilin D inhibitor) | 2011 | No |  |  | No |  |  | No |  |
|  | Cardiac arrest | N-methyl-4-isoleucine-cyclosporine / mPTP inhibitor | 2019 | No |  |  | No |  |  | No |  |
|  | Cardiac arrest | SS-31 | 2019 | No |  |  | No |  |  | No |  |
|  | Cardiac arrest | ATP sensitive potassium channel opener Nicorandil | 2000 | No |  |  | No |  |  | No |  |
|  | Cardiac arrest | Mitochondrial ATP-sensitive potassium opener Diazoxide | 2001 | No |  |  | No |  |  | No |  |
|  | Cardiac arrest | 2,3-Butanedione monoxime, myosin inhibitor | 2000 | No |  |  | No |  |  | No |  |
|  | Cardiac arrest | 3-methyladenine, autophagy inhibitor | 2016 | No |  |  | No |  |  | No |  |
|  | Cardiac arrest | Aquaporin-4 inhibitor AER-271 | 2019 | No |  |  | No |  |  | No |  |
|  | Cardiac arrest | Brain-derived neurotrophic factor | 2002 | No |  |  | No |  |  | No |  |
|  | Cardiac arrest | Calcium-permeable ion channel transient receptor potential-M2 inhibitor tatM2NX | 2020 | No |  |  | No |  |  | No |  |
|  | Cardiac arrest | Calicum/calmodulin-dependent protein kinase inhibition IItatCN21/tatCN19o | 2017 | No |  |  | No |  |  | No |  |
|  | Cardiac arrest | Dimethyl malonate, succinate dehydrogenase (complex II) inhibitor | 2018 | No |  |  | No |  |  | No |  |
|  | Cardiac arrest | Dimethyloxalylglycine / HIF-alfa stabilizer | 2016 | No |  |  | No |  |  | No |  |
|  | Cardiac arrest | ERK-inhibitor U0126 | 2004 | No |  |  | No |  |  | No |  |
|  | Cardiac arrest | Fingolimod, sphingosine 1-phosphate receptor agonist | 2019 | No |  |  | No |  |  | No |  |
|  | Cardiac arrest | Hemin, neuroglobinactivator | 2011 | No |  |  | No |  |  | No |  |
|  | Cardiac arrest | HET0016, eicosanoid inhibitor | 2015 | No |  |  | No |  |  | No |  |
|  | Cardiac arrest | Hydroxylamine / cystathione-beta-synthase inhibitor | 2003 | No |  |  | No |  |  | No |  |
|  | Cardiac arrest | MK-801, NMDA-antagonist | 2001 | No |  |  | No |  |  | No |  |
|  | Cardiac arrest | ML228 / HIF-alfa activator | 2016 | No |  |  | No |  |  | No |  |
|  | Cardiac arrest | Nogo-A antibody | 2020 | No |  |  | No |  |  | No |  |
|  | Cardiac arrest | Opioid receptor agonists BW373U86 or D-Ala2-D-Leu5 enkephalin | 2014 | No |  |  | No |  |  | No |  |
|  | Cardiac arrest | PD98059 (MEK-inhibitor) | 2016 | No |  |  | No |  |  | No |  |
|  | Cardiac arrest | pifithrin-μ (p53-inhibitor) | 2016 | No |  |  | No |  |  | No |  |
|  | Cardiac arrest | Polyethylene glycol-20k | 2018 | No |  |  | No |  |  | No |  |
|  | Cardiac arrest | PPBP (4-phenyl-1-(4-phenylethyl)-piperidine) | 2010 | No |  |  | No |  |  | No |  |
|  | Cardiac arrest | Protease-activated receptor 1 antagonist SCH79797 | 2016 | No |  |  | No |  |  | No |  |
|  | Cardiac arrest | Pyruvate dehydrogenase kinase inhibitor Dichloroacetate | 2001 | No |  |  | No |  |  | No |  |
|  | Cardiac arrest | SB-3CT(MMP9 inhibition) | 2009 | No |  |  | No |  |  | No |  |
|  | Cardiac arrest | Sigma-1 receptor Cutamesine | 2019 | No |  |  | No |  |  | No |  |
|  | Cardiac arrest | Transient receptor potential M2 clotrimazole | 2006 | No |  |  | No |  |  | No |  |
|  | Cardiac arrest | L-NAME | 2002 | No |  |  | No |  |  | No |  |
|  | Cardiac arrest | Nitroglycerine | 2003 | No |  |  | No |  |  | No |  |
|  | Cardiac arrest | Sodium nitroprusside | 2002 | No |  |  | No |  |  | No |  |
|  | Cardiac arrest | Acetyl-l-carnitine | 2003 | No |  |  | No |  |  | No |  |
|  | Cardiac arrest | Annexin A1 | 2019 | No |  |  | No |  |  | No |  |
|  | Cardiac arrest | Cytidine diphosphate choline | 2008 | No |  |  | No |  |  | No |  |
|  | Cardiac arrest | FX06 (Fibrin derived peptide Beta 15-42) | 2016 | No |  |  | No |  |  | No |  |
|  | Cardiac arrest | Glutamine | 2000 | No |  |  | No |  |  | No |  |
|  | Cardiac arrest | Nerve growth factor mimetic GK-2 | 2015 | No |  |  | No |  |  | No |  |
|  | Cardiac arrest | Orexin-A | 2006 | No |  |  | No |  |  | No |  |
|  | Cardiac arrest | Urocortin | 2004 | No |  |  | No |  |  | No |  |
|  | Cardiac arrest | zDEVD FMK (caspase 3 inhibitor) | 2008 | No |  |  | No |  |  | No |  |
|  | Cardiac arrest | Adenosine monophosphate | 2000 | No |  |  | No |  |  | No |  |
|  | Cardiac arrest | Cannabinoid receptor agonist WIN55, 212-2 | 2010 | No |  |  | No |  |  | No |  |
|  | Cardiac arrest | Cholecystokinin | 2011 | No |  |  | No |  |  | No |  |
|  | Cardiac arrest | Dihydrocapsaicin transient receptor potential vanilloid type 1 | 2010 | No |  |  | No |  |  | No |  |
|  | Cardiac arrest | HBN-1 (ethanol, vasopressin and lidocaine) | 2012 | No |  |  | No |  |  | No |  |
|  | Cardiac arrest | Quinpirole, dopamine receptor 2 agonist | 2012 | No |  |  | No |  |  | No |  |
|  | Cardiac arrest | Captopril | 2000 | No |  |  | No |  |  | No |  |
|  | Cardiac arrest | Centhaquin | 2017 | No |  |  | No |  |  | No |  |
|  | Cardiac arrest | Dobutamine | 2001 | No |  |  | No |  |  | No |  |
|  | Cardiac arrest | Endothelin A receptor antagonist BQ123 | 2000 | No |  |  | No |  |  | No |  |
|  | Cardiac arrest | Levosimendan | 2000 | No |  |  | No |  |  | No |  |
|  | Cardiac arrest | Milrinone | 2001 | No |  |  | No |  |  | No |  |
|  | Cardiac arrest | Palmitic acid methyl ester | 2014 | No |  |  | No |  |  | No |  |
|  | Cardiac arrest | Prostaglandin E1 | 2017 | No |  |  | No |  |  | No |  |
|  | Cardiac arrest | Alpha-methylnoradrenaline / alfa-2 adrenoceptor agonist | 2001 | No |  |  | No |  |  | No |  |
|  | Cardiac arrest | Angiotensin II | 2001 | No |  |  | No |  |  | No |  |
|  | Cardiac arrest | Conivaptan | 2016 | No |  |  | No |  |  | No |  |
|  | Cardiac arrest | Diaspirin cross-linked hemoglobin (DCLHb) | 2001 | No |  |  | No |  |  | No |  |
|  | Cardiac arrest | Dopamine | 2005 | No |  |  | No |  |  | No |  |
|  | Cardiac arrest | Endothelin-1 | 2000 | No |  |  | No |  |  | No |  |
|  | Cardiac arrest | Methylene blue | 2006 | No |  |  | No |  |  | No |  |
|  | Cardiac arrest | Naloxone | 2006 | No |  |  | No |  |  | No |  |
|  | Cardiac arrest | Pralidoxime, cholinesterase reactivator | 2019 | No |  |  | No |  |  | No |  |
|  | Cardiac arrest | Terlipressin | 2010 | No |  |  | No |  |  | No |  |
|  | Cardiac arrest | Vasopressin | 2000 | Yes | 2001 | Neutral | Yes | 2001 | Neutral | No |  |
|  | Cardiac arrest | Niacin | 2013 | No |  |  | No |  |  | No |  |
|  | Cardiac arrest | Thiamine | 2016 | No |  |  | No |  |  | No |  |
|  | Cardiac arrest | Vitamin C | 2002 | No |  |  | No |  |  | No |  |
|  | Cardiac arrest | Rolipram, PDE4-inhibitor | 2003 | No |  |  | No |  |  | No |  |
|  | Cardiac arrest | Topiramate | 2001 | No |  |  | No |  |  | No |  |
| Macedo, 2022: Potential therapeutic effects of green tea on obese lipid profile - a systematic review | Obesity, green tea, hyperlipidemia | Green tea | 2009 | Yes | 2010 | Positive | Yes | 2010 | Positive | No |  |
| Maskery, 2021: Glucagon-like peptide-1 receptor agonists as neuroprotective agents for ischemic stroke: a systematic scoping review | Stroke | Glucagon-like peptide-1 receptor agonists | 2011 | Yes | 2015 | Positive | Yes | 2015 | Positive | No |  |
| Metzler, 2020: Ibrutinib in Gynecological Malignancies and Breast Cancer: A Systematic Review | Gynecological cancer | Ibrutinib | 2015 | Yes | 2019 | Positive | Yes | 2019 | Positive | No |  |
| Miller, 2014: Inhaled anticoagulation regimens for the treatment of smoke inhalation-associated acute lung injury: a systematic review | Inhalation trauma | Inhaled anticoagulants | 1986 | Yes | 1998 | Positive | No |  |  | No |  |
| Miranda, 2012: Recombinant human activated protein C as a disease modifier in severe acute pancreatitis: systematic review of current evidence | Pancreatitis | Activated protein C | 2005 | Yes | 2010 | Neutral | Yes | 2010 | Neutral | No |  |
| Montroy, 2020: The effects of resistant starches on inflammatory bowel disease in preclinical and clinical settings: a systematic review and meta-analysis | Inflammatory bowel disease | Resistant starches | 2003 | Yes | 1996 | Positive | Yes | 1996 | Positive | No |  |
| Moran, 2015: Biological augmentation and tissue engineering approaches in meniscus surgery | Meniscus surgery | Biological augmentation | 2009 | Yes | 2011 | Neutral | Yes | 2014 | Positive | No |  |
| Naaktgeboren, 2021: Efficacy of Physical Exercise to Offset Anthracycline-Induced Cardiotoxicity: A Systematic Review and Meta-Analysis of Clinical and Preclinical Studies | Cardiotoxicity, exercise | Physical exercise | 1985 | Yes | 2017 | Positive | Yes | 2018 | Neutral | No |  |
| Murray, 2014: The effects of exercise on cognition in Parkinson's disease: a systematic review | Parkinson's disease, exercise | Physical exercise | 2004 | Yes | 2009 | Positive | Yes | 2011 | Positive | No |  |
| Noorlag, 2019: Treatment of malignant gliomas with ketogenic or caloric restricted diets: A systematic review of preclinical and early clinical studies | Glioma, diet | Ketogenic diet | 2007 | Yes | 2012 | Positive | No |  |  | No |  |
|  | Glioma, diet | Calorie-restricted diet | 2012 | Yes | 1995 | Positive | No |  |  | No |  |
|  | Glioma, diet | Ketone supplementation | 2010 | No |  |  | No |  |  | No |  |
|  | Glioma, diet | Short-term starvation | 2008 | Yes | 2010 | Positive | No |  |  | No |  |
| Oehme, 2015: Cell-Based Therapies Used to Treat Lumbar Degenerative Disc Disease: A Systematic Review of Animal Studies and Human Clinical Trials | Cell-based therapy, degenerative disc | Condrocytes to repair lumbar intervertebral disc | 1998 | Yes | 2007 | Positive | No |  |  | No |  |
|  | Cell-based therapy, degenerative disc | Stem cells/progenitor cells to regenerate lumbar intervertebral disc | 2004 | Yes | 2006 | Positive | No |  |  | No |  |
| Pani, 2020: Inositol and Non-Alcoholic Fatty Liver Disease: A Systematic Review on Deficiencies and Supplementation | Fatty liver disease, inositol | Inositol | 1994 | Yes | 2019 | Neutral | Yes | 2019 | Neutral | No |  |
| Peisl, 2021: Therapeutic targeting of STAT3 pathways in pancreatic adenocarcinoma: A systematic review of clinical and preclinical literature | Cancer, chemotherapy | GSI IX | 2013 | No |  |  | No |  |  | No |  |
|  | Cancer, chemotherapy | Paclitaxel | 1987 | Yes | 2004 | Positive | Yes | 2004 | Positive | No |  |
|  | Cancer, chemotherapy | Gemcitabine | 1993 | Yes | 1997 | Positive | Yes | 1997 | Positive | Yes | 1996 |
|  | Cancer, chemotherapy | Embelin | 2014 | No |  |  | No |  |  | No |  |
|  | Cancer, chemotherapy | FLLL 32 | 2016 | No |  |  | No |  |  | No |  |
|  | Cancer, chemotherapy | 5-Fluorouracil | 1983 | Yes | 1965 | Positive | Yes | 1965 | Positive | Yes | 1960 |
|  | Cancer, chemotherapy | IL-9 antibody | 2019 | No |  |  | No |  |  | No |  |
|  | Cancer, chemotherapy | LTP-1 | 2016 | No |  |  | No |  |  | No |  |
|  | Cancer, chemotherapy | Abraxane | 1987 | Yes | 1994 | Positive | Yes | 1994 | Positive | Yes | 2013 |
|  | Cancer, chemotherapy | Cobemetinib | 2017 | No |  |  | No |  |  | No |  |
|  | Cancer, chemotherapy | Ruxolitinib | 2015 | Yes | 2015 | Neutral | Yes | 2015 | Neutral | No |  |
|  | Cancer, chemotherapy | SZC015 | 2018 | No |  |  | No |  |  | No |  |
| Plemel, 2015: Over-the-counter anti-oxidant therapies for use in multiple sclerosis: A systematic review | multiple sclerosis, antioxidant | Luteolin | 2004 | No |  |  | No |  |  | No |  |
|  | multiple sclerosis, antioxidant | Quercetin | 2004 | No |  |  | No |  |  | No |  |
|  | multiple sclerosis, antioxidant | Curcumin | 2005 | No |  |  | No |  |  | No |  |
|  | multiple sclerosis, antioxidant | Resveratrol | 2007 | No |  |  | No |  |  | No |  |
|  | multiple sclerosis, antioxidant | Vitamin A | 1968 | Yes | 2012 | Positive | No |  |  | No |  |
|  | multiple sclerosis, antioxidant | Vitamin E | 2009 | Yes | 2013 | Neutral | Yes | 2013 | Neutral | No |  |
|  | multiple sclerosis, antioxidant | Alpha lipoic acid | 2002 | Yes | 2005 | Positive | Yes | 2014 | Positive | No |  |
| Porflitt, 2022: Effects of aerobic exercise on fibroblast growth factor 21 in overweight and obesity. A systematic review | Exercise, obesity | Aerobic exercise | 2012 | Yes | 2015 | Positive | Yes | 2019 | Neutral | No |  |
| Ramos, 2020: Melatonin's efficacy in stroke patients a matter of dose? A systematic review | Stroke, melatonin | Melatonin | 1996 | Yes | 2001 | Positive | Yes | 2001 | Positive | No |  |
| Ramos, 2022: Mesenchymal stem cell therapy for focal epilepsy: A systematic review of preclinical models and clinical studies | Stem cells, epilepsy | Mesenchymals stem cells | 2007 | Yes | 2017 | Positive | Yes | 2017 | Positive | No |  |
| Reis, 2018: The anxiolytic effect of probiotics: A systematic review and meta-analysis of the clinical and preclinical literature | Anxiety, probiotics | Probiotics | 2010 | Yes | 2014 | Positive | Yes | 2017 | Neutral | No |  |
| Rendon, 2022: Statins and Gliomas: A Systematic Review of the Preclinical Studies and Meta-Analysis of the Clinical Literature | Glioma/glioblastoma, statins | Atorvastatin | 1997 | Yes | 2012 | Positive | Yes | 2014 | Positive | No |  |
| Rinonapoli, 2021: Stem cells application in meniscal tears: a systematic review of pre-clinical and clinical evidence | Stem cells, meniscal tears | Stem cells | 2009 | Yes | 2008 | Positive | Yes | 2014 | Positive | No |  |
| Roffi, 2017_1: Platelet-rich plasma for the treatment of bone defects: from pre-clinical rational to evidence in the clinical practice. A systematic review | Platelet-rich plasma, bone regeneration | Platelet-rich plasma | 2005 | Yes | 2006 | Positive | Yes | 2006 | Positive | No |  |
| Roffi, 2017_2: The Role of Three-Dimensional Scaffolds in Treating Long Bone Defects: Evidence from Preclinical and Clinical Literature-A Systematic Review | 3D scaffolds, bone regeneration | 3D scaffolds | 1995 | Yes | 2001 | Positive | Yes | 2001 | Positive | No |  |
| Salem, 2016: Moving from the Dish to the Clinical Practice: A Decade of Lessons and Perspectives from the Pre-Clinical and Clinical Stem Cell Studies for Alzheimer's Disease | Alzheimer's disease, stem cells | Stem cells | 2006 | Yes | 2015 | Positive | No |  |  | No |  |
| Schneider, 2021: Performance of image guided navigation in laparoscopic liver surgery â€“ A systematic review | Laparoscopic liver surgery, robotic surgery | Image-guided surgery | 2006 | Yes | 2011 | Positive | No |  |  | No |  |
| Schmidt, 2020: Why Most Acute Stroke Studies Are Positive in Animals but Not in Patients: A Systematic Comparison of Preclinical, Early Phase, and Phase 3 Clinical Trials of Neuroprotective Agents | Stroke | Albumin | 1989 | Yes | 1985 | Positive | Yes | 1985 | Positive | No |  |
|  | Stroke | Aptiganel | 1993 | Yes | 1999 | Positive | Yes | 1999 | Positive | No |  |
|  | Stroke | BMS-204352 | 2001 | Yes | 2005 | Positive | Yes | 2015 | Unclear | No |  |
|  | Stroke | Candesartan | 1996 | Yes | 2003 | Positive | Yes | 2003 | Positive | No |  |
|  | Stroke | Chlomethiazole | 2000 | Yes | 1999 | Neutral | Yes | 1999 | Neutral | No |  |
|  | Stroke | Citicoline | 1996 | Yes | 1997 | Positive | Yes | 1997 | Positive | No |  |
|  | Stroke | Diazepam | 2000 | Yes | 2005 | Positive | Yes | 2019 | Positive | No |  |
|  | Stroke | Ebselen | 2000 | Yes | 1998 | Positive | Yes | 1998 | Positive | No |  |
|  | Stroke | Edaravone | 1989 | Yes | 2003 | Positive | Yes | 2003 | Positive | No |  |
|  | Stroke | Eliprodil | 1988 | Yes | 2000 | Neutral | Yes | 2000 | Neutral | No |  |
|  | Stroke | Enlimomab | 2003 | Yes | 2001 | Negative | Yes | 2001 | Negative | No |  |
|  | Stroke | Epoetin alfa | 2013 | Yes | 2014 | Positive | Yes | 2014 | Positive | No |  |
|  | Stroke | Flunarizine | 1984 | Yes | 1990 | Positive | Yes | 1996 | Neutral | No |  |
|  | Stroke | Gavestinel | 1997 | Yes | 1999 | Neutral | Yes | 2006 | Neutral | No |  |
|  | Stroke | GM1 ganglioside | 1986 | Yes | 1988 | Neutral | Yes | 1989 | Neutral | No |  |
|  | Stroke | Isradipine | 1986 | Yes | 1988 | Positive | Yes | 2001 | Unclear | No |  |
|  | Stroke | Lubeluzole | 1996 | Yes | 1996 | Positive | Yes | 1996 | Positive | No |  |
|  | Stroke | Naftidrofuryl | 1983 | Yes | 1984 | Positive | Yes | 1990 | Neutral | No |  |
|  | Stroke | Nimodipine | 1982 | Yes | 1984 | Positive | Yes | 2016 | Neutral | No |  |
|  | Stroke | NXY-059 | 2001 | Yes | 2006 | Positive | Yes | 2006 | Positive | No |  |
|  | Stroke | ONO-2506 | 2002 | Yes | 2006 | Positive | Yes | 2006 | Positive | No |  |
|  | Stroke | Piracetam | 1990 | Yes | 1988 | Unclear | Yes | 1998 | Unclear | No |  |
|  | Stroke | Repinotan | 1998 | Yes | 2005 | Positive | Yes | 2009 | Neutral | No |  |
|  | Stroke | Selfotel | 1990 | Yes | 1995 | Positive | Yes | 1995 | Positive | No |  |
|  | Stroke | Tirilazad | 1988 | Yes | 1996 | Neutral | Yes | 1996 | Neutral | No |  |
| Serpa, 2014: Ventilation with lower tidal volumes for critically ill patients without the acute respiratory distress syndrome: a systematic translational review and meta-analysis | ARDS, mechanical ventilation | Ventilation with lower tidal volumes | 1990 | Yes | 1990 | Positive | Yes | 2010 | Positive | No |  |
| Stevens, 2022: Photobiomodulation in acute traumatic brain injury: a systematic review and meta-analysis | Traumatic brain injury | Photobiomodulation | 2007 | Yes | 2020 | Positive | Yes | 2020 | Positive | No |  |
| Timur, 2020: Chondroprotective Actions of Selective COX-2 Inhibitors In Vivo: A Systematic Review | Osteoarthritis | Celecoxib | 2006 | Yes | 2002 | Neutral | No |  |  | No |  |
| Verboven, 2019: Effect of Exercise Intervention on Cardiac Function in Type 2 Diabetes Mellitus: A Systematic Review | Diabetes mellitus, cardiac function | Physical exercise | 2009 | Yes | 2007 | Positive | Yes | 2010 | Positive | No |  |
| Veronesi, 2021: Biosynthetic scaffolds for partial meniscal loss: A systematic review from animal models to clinical practice | Osteoarthritis, meniscus | Biosynthetic scaffolds | 2001 | Yes | 2007 | Positive | No |  |  | No |  |
| Versteegden, 2017: Tissue Engineering of the Urethra: A Systematic Review and Meta-analysis of Preclinical and Clinical Studies | Urethra, reconstructive surgery | Tissue-engineering | 1971 | Yes | 1989 | Positive | No |  |  | No |  |
| Voulgaropoulou, 2019: The effect of curcumin on cognition in Alzheimer's disease and healthy aging: A systematic review of pre-clinical and clinical studies | Alzheimer's disease | Curcumin | 2001 | Yes | 2008 | Positive | Yes | 2008 | Positive | No |  |
| Zhang, 2020: The pharmacological activity of epigallocatechin-3-gallate (EGCG) on Alzheimer's disease animal model: A systematic review | Alzheimer's Disease | Epigallocatechin gallate | 2005 | Yes | 2018 | Positive | No |  |  | No |  |
| Zoerle, 2012: Pharmacologic reduction of angiographic vasospasm in experimental subarachnoid hemorrhage: systematic review and meta-analysis | Vasospasms, subarachnoid hemorrhage | Tirilazad | 1989 | Yes | 1995 | Positive | Yes | 1995 | Positive | No |  |
|  | Vasospasms, subarachnoid hemorrhage | Erythropoietin | 2002 | Yes | 2007 | Unclear | Yes | 2007 | Unclear | No |  |
|  | Vasospasms, subarachnoid hemorrhage | Fasudil | 1992 | Yes | 1990 | Positive | Yes | 1992 | Positive | No |  |
|  | Vasospasms, subarachnoid hemorrhage | Tissue plasminogen activator | 1989 | Yes | 1991 | Positive | Yes | 1994 | Positive | No |  |
|  | Vasospasms, subarachnoid hemorrhage | Magnesium | 2004 | Yes | 2003 | Positive | Yes | 2005 | Unclear | No |  |
|  | Vasospasms, subarachnoid hemorrhage | Nimodipine | 1984 | Yes | 1983 | Positive | Yes | 1983 | Positive | Yes | 1988 |

The data underlying this table can be found on <https://osf.io/frjm4> (Sheet: *Journey*).
