# Supplementary material for: Analysis of animal-to-human translation shows that only 5% of animal-tested therapeutic interventions obtain regulatory approval for human applications
Source: PLoS Biol. 2024 Jun 13;22(6):e3002667. doi: 10.1371/journal.pbio.3002667 (PMC11175415; doi:10.1371/journal.pbio.3002667)
Supplement: S2 Table — (DOCX) [file pbio.3002667.s012.docx]

**Supplementary Table 2**: Translational assessment of interventions for neurological diseases.

| **Disease/condition** | **Intervention** | **Study** | **Animal studies** | **Human studies** | **Summary** |
| --- | --- | --- | --- | --- | --- |
| Stroke | Aerobic exercise | Austin, 2014 [1] | 47 | 0 | Forced exercise was beneficial in animal models of stroke. No human studies identified, but discussion about timing and endurance of exercise therapy in stroke patients. |
| Stroke | Dose articulation studies | Dalton, 2021 [2] | 3 | 39 | Most studies reported an efficacy outcome; however, only 65% reported dose and 45% reported safety. Most studies were at high risk of bias, and the mean percentage reporting of intervention description and quality was 61% and 67%, respectively. |
| Stroke | Remote ischemic conditioning | Hansen, 2021 [3] | 22 | 8 | Non-clinically relevant methodological approaches in animals, e.g., ischemic conditioning prior to an ischemic insult which is not relevant for stroke. Poor reporting and high risk of bias, mostly for animal studies. Multiple other discrepancies in the design and performance of conditioning which could pose barriers for successful translation, e.g., animal hindlimbs versus human fore-limb conditioning; anesthetic use in animal experiments versus no anesthesia in humans; lack of relevant comorbidities in animas. |
| Stroke | Mesenchymal stem cells | Lalu, 2020 [4] | 76 | 11 | Citation analysis: Animal studies were rarely cited in human studies. Mostly positive animal studies. One randomized controlled phase 2 trials in humans negative. |
| Stroke | S-Nitrosoglutathione | Liu, 2020 [5] | 6 | 4 | S-Nitrosoglutathione in stroke with positive animal and human studies. |
| Stroke | Glucagon-like peptide-1 receptor agonists | Maskery, 2021 [6] | 35 | 22 | Glucagon-like peptide-1 receptor agonists for stroke with positive animal studies and favorable preliminary observations in humans. |
| Stroke | Melatonin | Ramos, 2020 [7] | 41 | 6 | Melatonin with positive impact on stroke in animals, in humans less clear. Doses very low in humans (compared to animals) |
| Stroke | A comprehensive list of drugs for acute ischemic stroke | Schmidt-Pogoda, 2020 [8] | 209 | 125 | Large scale systematic review and meta-analysis assessed preclinical-to-clinical development of 37 treatments for acute ischemic stroke, including 50 phase 3 clinical trials, 75 early clinical trials, and 209 animal studies. There was a stepwise decline in efficacy from animal studies to early clinical studies to phase 3 clinical studies, which was explained by lack of rigour of preclinical studies (i.e., lack of randomization and blinding), differences in study design such as using different outcomes, as well as low power of animal studies and early clinical trials. |
| Intracerebral hemorrhage | Hypothermia | Baker, 2021 [9] | 21 | 5 | Hypothermia showed higher effect sizes after bleeding in animals compared to humans. Also, humans undergo delayed and longer cooling periods compared to animals. |
| Subarachnoid hemorrhage | Comprehensive list of drugs for vasospasms | Zoerle, 2012 [10] | 70 | NA | Pharmacological strategies to treat vasospasms after subarachnoid hemorrhage. 70 papers comprising 74 comparisons. Some drugs were effective in some species and other drugs in other species. Considering animal studies individually, 55% were concordant with human trials. The only significant predictor of translational success was the day of vasospasm assessment, i.e., studies where vasospasms were assessed 3 days after subarachnoid hemorrhage were more concordant with human trials. This SR showed that drugs that are effective at reducing vasospasms in animal models of subarachnoid hemorrhage were in some cases also effective in humans. Publication bias might overestimate the true effect. Poor quality of many animal studies. |
| Multiple sclerosis | Neuroprotective/neuroregenerative strategies | Allanach, 2021 [11] | 250 | 19 | Drug therapies were more easily translated to human trials compared to other intervention types such as exercise or diet regimens, likely due to more complex trial designs required for such therapies. |
| Multiple sclerosis | Probiotics | Blais, 2021 [12] | 31 | 6 | Probiotics with higher effect sizes in animals compared to humas. Putative reasons: human trials underpowered and confounded (e.g., through lifestyle) and animal studies with broader outcome measures. |
| Multiple sclerosis | Remyelination promoting therapies | Hooijmans, 2019 [13] | 103 | 25 | 28% of preclinically tested therapies for remyelination reached a clinical trial for MS. |
| Multiple sclerosis | 4-aminopyridine | Jensen, 2014 [14] | 16 | 19 | 4-Aminopyridine for MS with consistent results between animal and human studies. |
| Multiple sclerosis | Over-the-counter anti-oxidants | Plemel, 2015 [15] | NA | NA | Antioxidant therapy: application different - humans oral, animal intraperitoneally/subcutaneously. Antioxidant therapy was tolerated in animals and humans. Some antioxidants successfully tested in animals. Other antioxidants tested in humans with mixed results. |
| Alzheimer's disease | Ketogenic diet | Lilamand, 2020 [16] | 11 | 11 | Ketogenic diet improved cognition in animals and humans. |
| Alzheimer's disease | Stem cells | Salem, 2016 [17] | 65 | 13 | Stem cells with positive effects in animals but questionable effect in humans. Stem cells for Alzheimers disease with different anatomy, pathophysiology, and microenvironments between animals and humans as reasons for translational failure. |
| Alzheimer's disease | Curcumin | Voulgaropoulou, 2019 [18] | 32 | 5 | Methodological discrepancies between animal and human studies making a direct comparison difficult. However, none of the studies have reached Phase III in clinical trials, suggesting that curcumin has not fully met expectations. |
| Alzheimer's disease | Epigallocatechin-3-gallate | Zhang, 2020 [19] | 17 | 105 | ECGC with mostly positive effects in animal studies, but only 1/3 studies in Down syndrome with positive effects in humans |
| Parkinson's disease | Probiotics | Leta, 2021 [20] | 12 | 5 | Probiotics with beneficial effects on glucose levels, inflammation, motor and non-motor function in animals and humans. Only anti-oxidative in animals. |
| Parkinson's disease | Exercise | Murray, 2014 [21] | 6 | 8 | Exercise with beneficial effect on cognition in animals and humans. |
| Neurodegeneration/brain development | Iron | Agrawal, 2017 [22] | 34 | 14 | Iron with adverse effects in animal studies. Human studies did not assess high iron intakes. Dosing was comparable between preclinical and clinical studies. |
| Cognitive decline | Resveratrol | Khorshidi, 2021 [23] | 31 | 12 | Resveratrol showed beneficial effects on cognition in animal studies but not in human trials. Excessive doses for animals compared to humans (i.e., up to 1g/kg body weight for animals would correspond to 75 g/kg body weight for humans). |
| Dementia | Proteostasis modulators | Heard, 2018 [24] | 38 | 9 | Proteostasis inhibitors show beneficial effects on cognition in both animals and humans. |
| Dementia | Metformin | Kioussis, 2021 [25] | 91 | 23 | Metformin with modest cognitive benefits in animal models of dementia, but no such effects in humans. |
| Epilepsy | Branched-chain amino acids | Gruenbaum, 2019 [26] | 10 | 1 | Branched-chain amino acids with anticonvulsant effect in animals and humans (only 1 study). |
| Epilepsy | Mesenchymal stem cells | Ramos, 2022 [27] | 11 | 4 | Stem cell therapy for seizure with beneficial effect in both animals and humans. |
| Epilepsy | Transcranial direct-current stimulation | Regner, 2018 [28] | 5 | 12 | Only few findings from animal studies have been translated to humans and several clinically tested paradigms have not been tested in the preclinical setting. Animal studies mostly positive. Different methodological approaches between animals and humans, e.g., electric currents being in the micro-Ampere range for animals and milli-Ampere range for humans. |
| Amyotrophc lateral sclerosis | Regulatory T cells | Rajabinejad, 2020 [29] | 3 | 1 | T-Regs for ALS with beneficial effect in animals, but only scarce data in humans (1 study without a control group). |
| Pain | Cyclodextrins | Brito, 2015 [30] | 15 | 7 | Cyclodextrins with analgesic effect in animals and humans. |
| Pain | Cannabinoids | Linde, 2021 [31] | 40 | 6 | Cannabinoids with analgesic effects in animals and humans. Yet different outcome measures used for these domains. Animals with different pain pathomechanisms. |
| Pain | Cannabinoids | Nielsen, 2017 [32] | 19 | 9 | Opioid-sparing effects of cannabinoids in animals but only very marginal evidence in one small case series for humans. |
| Traumatic brain injury | Nutritional interventions | McGeown, 2021 [33] | 43 | 0 | Amino acids/fatty acids with promising results in animal traumatic brain injury studies. No clinical studies identified. |
| Traumatic brain injury | N-Acetylcysteine | Bhatti, 2017 [34] | 20 | 3 | N-acetylcystein with beneficial effect after brain injury in animals and humans. |
| Traumatic brain injury | Photobiomodulation | Stevens, 2022 [35] | 17 | 1 | Photobiomodulation in animal studies seemed promising, the translational success is still unclear with at best few case series in humans being published. |
| Spinal cord injury | Riluzole, cethrin, early surgical decompression | Cadotte, 2011 [36] | 19 | 22 | Assessed the translation of three selected therapies with promising early clinical trials for spinal cord injury (riluzole, a sodium channel blocker with potential neuroprotective effects, Cethrin, a Rho-antagonist, and early surgical decompression animal to clinical trials). |
| Segmental nerve defects | Autologous nerve grafts | Heinzel, 2021[37] | 8 | 2 | Discrepancies regarding the methodological approaches between animals and humans. Grafts were effective in animals but only modestly effective in humans. |
| Peripheral nerve injury | Electrical stimulation | Ransom, 2020 [38] | NA | NA | Electrical stimulation promoted nerve regeneration in animals and humans. |
| Neurological diseases | Vitamin C | Kangisser, 2021 [39] | 22 | 11 | Vitamin C for neurological insult with positive animal studies, but no effect in human studies. Yet much higher relative doses of Vitamin C for animal studies. |
| Neuromodulation | Low intensity transcranial ultrasound stimulation | Kim, 2021 [40] | 24 | 2 | Low intensity transcranial ultrasound stimulation on neuromodulation with positive effects in animals but not in large animals or humans. |
| Brain radiation | Brain radiation | Perez, 2022 [41] | 47 | 108 | Radiation-induded brain injury: no animal model can mimic human phenotype (only partial resemblance). Neuroimaging studies not completely consistent between rodents and humans, e.g., no cerebral microbleeds in rodents. |

The data underlying this table can be found on <https://osf.io/frjm4> (Sheet: *Curated*).

**References**

1. Austin MW, Ploughman M, Glynn L, Corbett D. Aerobic exercise effects on neuroprotection and brain repair following stroke: a systematic review and perspective. Neuroscience Research. 2014;87:8-15. doi: 10.1016/j.neures.2014.06.007. PubMed PMID: 24997243.

2. Dalton EJ, Churilov L, Lannin NA, Corbett D, Campbell BCV, Hayward KS. Early-phase dose articulation trials are underutilized for post-stroke motor recovery: A systematic scoping review. Annals of Physical & Rehabilitation Medicine. 2022;65(1):101487. doi: 10.1016/j.rehab.2021.101487. PubMed PMID: 33429089.

3. Hansen LF, Nielsen NSK, Christoffersen LC, Kruuse C. Translational challenges of remote ischemic conditioning in ischemic stroke - a systematic review. Annals of Clinical & Translational Neurology. 2021;8(8):1720-9. doi: 10.1002/acn3.51405. PubMed PMID: 34133841.

4. Lalu MM, Montroy J, Dowlatshahi D, Hutton B, Juneau P, Wesch N, et al. From the Lab to Patients: a Systematic Review and Meta-Analysis of Mesenchymal Stem Cell Therapy for Stroke. Translational Stroke Research. 2020;11(3):345-64. doi: 10.1007/s12975-019-00736-5. PubMed PMID: 31654281.

5. Liu S, Lowe F, Yu W, Zheng H, Singh I, Feng W. Investigation of s-nitrosoglutathione in stroke: A systematic review of literature in pre-clinical and clinical research. Stroke. 2019;50. doi: 10.1161/str.50.suppl_1.TP105.

6. Maskery MP, Holscher C, Jones SP, Price CI, Strain WD, Watkins CL, et al. Glucagon-like peptide-1 receptor agonists as neuroprotective agents for ischemic stroke: a systematic scoping review. Journal of Cerebral Blood Flow & Metabolism. 2021;41(1):14-30. doi: 10.1177/0271678X20952011. PubMed PMID: 32954901.

7. Ramos E, Farre-Alins V, Egea J, Lopez-Munoz F, Reiter RJ, Romero A. Melatonin's efficacy in stroke patients; a matter of dose? A systematic review. Toxicology & Applied Pharmacology. 2020;392:114933. doi: 10.1016/j.taap.2020.114933. PubMed PMID: 32112789.

8. Schmidt-Pogoda A, Bonberg N, Koecke MHM, Strecker JK, Wellmann J, Bruckmann NM, et al. Why Most Acute Stroke Studies Are Positive in Animals but Not in Patients: A Systematic Comparison of Preclinical, Early Phase, and Phase 3 Clinical Trials of Neuroprotective Agents. Annals of Neurology. 2020;87(1):40-51. doi: 10.1002/ana.25643. PubMed PMID: 31714631.

9. Baker TS, Durbin J, Troiani Z, Ascanio-Cortez L, Baron R, Costa A, et al. Therapeutic hypothermia for intracerebral hemorrhage: Systematic review and meta-analysis of the experimental and clinical literature. International Journal of Stroke. 2022;17(5):506-16. doi: 10.1177/17474930211044870. PubMed PMID: WOS:000700424700001.

10. Zoerle T, Ilodigwe DC, Wan H, Lakovic K, Sabri M, Ai J, et al. Pharmacologic reduction of angiographic vasospasm in experimental subarachnoid hemorrhage: systematic review and meta-analysis. Journal of Cerebral Blood Flow & Metabolism. 2012;32(9):1645-58. doi: 10.1038/jcbfm.2012.57. PubMed PMID: 22534672.

11. Allanach JR, Farrell JW, 3rd, Mesidor M, Karimi-Abdolrezaee S. Current status of neuroprotective and neuroregenerative strategies in multiple sclerosis: A systematic review. Multiple Sclerosis. 2022;28(1):29-48. doi: 10.1177/13524585211008760. PubMed PMID: 33870797.

12. Blais LL, Montgomery TL, Amiel E, Deming PB, Krementsov DN. Probiotic and commensal gut microbial therapies in multiple sclerosis and its animal models: a comprehensive review. Gut Microbes. 2021;13(1):1943289. doi: 10.1080/19490976.2021.1943289. PubMed PMID: 34264791.

13. Hooijmans CR, Hlavica M, Schuler FAF, Good N, Good A, Baumgartner L, et al. Remyelination promoting therapies in multiple sclerosis animal models: a systematic review and meta-analysis. Scientific Reports. 2019;9(1):822. doi: 10.1038/s41598-018-35734-4. PubMed PMID: 30696832.

14. Jensen HB, Ravnborg M, Dalgas U, Stenager E. 4-Aminopyridine for symptomatic treatment of multiple sclerosis: a systematic review. Therapeutic Advances in Neurological Disorders. 2014;7(2):97-113. doi: 10.1177/1756285613512712. PubMed PMID: 24587826.

15. Plemel JR, Juzwik CA, Benson CA, Monks M, Harris C, Ploughman M. Over-the-counter anti-oxidant therapies for use in multiple sclerosis: A systematic review. Multiple Sclerosis. 2015;21(12):1485-95. doi: 10.1177/1352458515601513. PubMed PMID: 26286700.

16. Lilamand M, Porte B, Cognat E, Hugon J, Mouton-Liger F, Paquet C. Are ketogenic diets promising for Alzheimer's disease? A translational review. Alzheimer's Research & Therapy. 2020;12(1):42. doi: 10.1186/s13195-020-00615-4. PubMed PMID: 32290868.

17. Salem H, Rocha NP, Colpo GD, Teixeira AL. Moving from the Dish to the Clinical Practice: A Decade of Lessons and Perspectives from the Pre-Clinical and Clinical Stem Cell Studies for Alzheimer's Disease. Journal of Alzheimer's Disease. 2016;53(3):1209-30. doi: 10.3233/JAD-160250. PubMed PMID: 27392861.

18. Voulgaropoulou SD, van Amelsvoort T, Prickaerts J, Vingerhoets C. The effect of curcumin on cognition in Alzheimer's disease and healthy aging: A systematic review of pre-clinical and clinical studies. Brain Research. 2019;1725:146476. doi: 10.1016/j.brainres.2019.146476. PubMed PMID: 31560864.

19. Zhang S, Zhu Q, Chen JY, OuYang D, Lu JH. The pharmacological activity of epigallocatechin-3-gallate (EGCG) on Alzheimer's disease animal model: A systematic review. Phytomedicine. 2020;79:153316. doi: 10.1016/j.phymed.2020.153316. PubMed PMID: 32942205.

20. Leta V, Chaudhuri KR, Milner O, Chung-Faye G, Metta V, Pariante CM, et al. Neurogenic and anti-inflammatory effects of probiotics in Parkinson's disease: A systematic review of preclinical and clinical evidence. Brain Behav Immun. 2021;98:59-73. doi: 10.1016/j.bbi.2021.07.026. PubMed PMID: WOS:000706632500006.

21. Murray DK, Sacheli MA, Eng JJ, Stoessl AJ. The effects of exercise on cognition in Parkinson's disease: a systematic review. Translational Neurodegeneration. 2014;3(1):5. doi: 10.1186/2047-9158-3-5. PubMed PMID: 24559472.

22. Agrawal S, Berggren KL, Marks E, Fox JH. Impact of high iron intake on cognition and neurodegeneration in humans and in animal models: a systematic review. Nutrition Reviews. 2017;75(6):456-70. doi: 10.1093/nutrit/nux015. PubMed PMID: 28505363.

23. Khorshidi F, Poljak A, Liu Y, Lo JW, Crawford JD, Sachdev PS. Resveratrol: A "miracle" drug in neuropsychiatry or a cognitive enhancer for mice only? A systematic review and meta-analysis. Ageing Research Reviews. 2021;65:101199. doi: 10.1016/j.arr.2020.101199. PubMed PMID: 33303422.

24. Heard DS, Tuttle CSL, Lautenschlager NT, Maier AB. Repurposing Proteostasis-Modifying Drugs to Prevent or Treat Age-Related Dementia: A Systematic Review. Frontiers in Physiology. 2018;9:1520. doi: 10.3389/fphys.2018.01520. PubMed PMID: 30425653.

25. Kioussis B, Tuttle CSL, Heard DS, Kennedy BK, Lautenschlager NT, Maier AB. Targeting impaired nutrient sensing with repurposed therapeutics to prevent or treat age-related cognitive decline and dementia: A systematic review. Ageing Research Reviews. 2021;67:101302. doi: 10.1016/j.arr.2021.101302. PubMed PMID: 33609776.

26. Gruenbaum SE, Chen EC, Sandhu MRS, Deshpande K, Dhaher R, Hersey D, et al. Branched-Chain Amino Acids and Seizures: A Systematic Review of the Literature. CNS Drugs. 2019;33(8):755-70. doi: 10.1007/s40263-019-00650-2. PubMed PMID: 31313139.

27. Ramos-Fresnedo A, Perez-Vega C, Domingo RA, Lee SJ, Perkerson RB, Zubair AC, et al. Mesenchymal stem cell therapy for focal epilepsy: A systematic review of preclinical models and clinical studies. Epilepsia. 2022;22:22. doi: 10.1111/epi.17266. PubMed PMID: 35451066.

28. Regner GG, Pereira P, Leffa DT, de Oliveira C, Vercelino R, Fregni F, et al. Preclinical to Clinical Translation of Studies of Transcranial Direct-Current Stimulation in the Treatment of Epilepsy: A Systematic Review. Frontiers in Neuroscience. 2018;12:189. doi: 10.3389/fnins.2018.00189. PubMed PMID: 29623027.

29. Rajabinejad M, Ranjbar S, Afshar Hezarkhani L, Salari F, Gorgin Karaji A, Rezaiemanesh A. Regulatory T cells for amyotrophic lateral sclerosis/motor neuron disease: A clinical and preclinical systematic review. Journal of Cellular Physiology. 2020;235(6):5030-40. doi: 10.1002/jcp.29401. PubMed PMID: 31788795.

30. Brito RG, Araujo AA, Quintans JS, Sluka KA, Quintans-Junior LJ. Enhanced analgesic activity by cyclodextrins - a systematic review and meta-analysis. Expert Opinion on Drug Delivery. 2015;12(10):1677-88. doi: 10.1517/17425247.2015.1046835. PubMed PMID: 26159048.

31. Linde LD, Ogryzlo CM, Choles CM, Cairns BE, Kramer JLK. Efficacy of topical cannabinoids in the management of pain: a systematic review and meta-analysis of animal studies. Regional Anesthesia & Pain Medicine. 2022;47(3):183-91. doi: 10.1136/rapm-2021-102719. PubMed PMID: 35012994.

32. Nielsen S, Sabioni P, Trigo JM, Ware MA, Betz-Stablein BD, Murnion B, et al. Opioid-Sparing Effect of Cannabinoids: A Systematic Review and Meta-Analysis. Neuropsychopharmacology. 2017;42(9):1752-65. doi: 10.1038/npp.2017.51. PubMed PMID: 28327548.

33. McGeown JP, Hume PA, Theadom A, Quarrie KL, Borotkanics R. Nutritional interventions to improve neurophysiological impairments following traumatic brain injury: A systematic review. Journal of Neuroscience Research. 2021;99(2):573-603. doi: 10.1002/jnr.24746. PubMed PMID: 33107071.

34. Bhatti J, Nascimento B, Akhtar U, Rhind SG, Tien H, Nathens A, et al. Systematic Review of Human and Animal Studies Examining the Efficacy and Safety of N-Acetylcysteine (NAC) and N-Acetylcysteine Amide (NACA) in Traumatic Brain Injury: Impact on Neurofunctional Outcome and Biomarkers of Oxidative Stress and Inflammation. Frontiers in neurology [electronic resource]. 2017;8:744. doi: 10.3389/fneur.2017.00744. PubMed PMID: 29387038.

35. Stevens AR, Hadis M, Milward MR, Ahmed Z, Belli A, Palin WM, et al. Photobiomodulation in acute traumatic brain injury: a systematic review and meta-analysis. Journal of Neurotrauma. 2022;13:13. doi: 10.1089/neu.2022.0140. PubMed PMID: 35698294.

36. Cadotte DW, Fehlings MG. Spinal cord injury: a systematic review of current treatment options. Clinical Orthopaedics & Related Research. 2011;469(3):732-41. doi: 10.1007/s11999-010-1674-0. PubMed PMID: 21080129.

37. Heinzel JC, Quyen Nguyen M, Kefalianakis L, Prahm C, Daigeler A, Hercher D, et al. A systematic review and meta-analysis of studies comparing muscle-in-vein conduits with autologous nerve grafts for nerve reconstruction. Scientific Reports. 2021;11(1):11691. doi: 10.1038/s41598-021-90956-3. PubMed PMID: 34083605.

38. Ransom SC, Shahrestani S, Lien BV, Tafreshi AR, Brown NJ, Hanst B, et al. Translational Approaches to Electrical Stimulation for Peripheral Nerve Regeneration. Neurorehabilitation and Neural Repair. 2020;34(11):979-85. doi: 10.1177/1545968320962508.

39. Kangisser L, Tan E, Bellomo R, Deane AM, Plummer MP. Neuroprotective Properties of Vitamin C: A Scoping Review of Pre-Clinical and Clinical Studies. Journal of Neurotrauma. 2021;38(16):2194-205. doi: 10.1089/neu.2020.7443. PubMed PMID: 33544035.

40. Kim T, Park C, Chhatbar PY, Feld J, Mac Grory B, Nam CS, et al. Effect of Low Intensity Transcranial Ultrasound Stimulation on Neuromodulation in Animals and Humans: An Updated Systematic Review. Frontiers in Neuroscience. 2021;15:620863. doi: 10.3389/fnins.2021.620863. PubMed PMID: 33935626.

41. Perez WD, Perez-Torres CJ. Neurocognitive and radiological changes after cranial radiation therapy in humans and rodents: a systematic review. International Journal of Radiation Biology. 2022:1-19. doi: 10.1080/09553002.2022.2074167. PubMed PMID: 35511499.
