# Supplementary material for: Analysis of animal-to-human translation shows that only 5% of animal-tested therapeutic interventions obtain regulatory approval for human applications
Source: PLoS Biol. 2024 Jun 13;22(6):e3002667. doi: 10.1371/journal.pbio.3002667 (PMC11175415; doi:10.1371/journal.pbio.3002667)
Supplement: S3 Table — (DOCX) [file pbio.3002667.s013.docx]

**Supplementary Table 3**: Translational assessment of interventions for diseases of the musculoskeletal system and connective tissue.

| **Disease/condition** | **Intervention** | **Study** | **Animal studies** | **Human studies** | **Summary** |
| --- | --- | --- | --- | --- | --- |
| Bone fracture/defects | Platelet-rich plasma | Roffi, 2017 [1] | 45 | 19 | Platelet rich plasma for bone repair had modest effects in animals and very small effects in humans. |
| Bone fracture/defects | 3D scaffolds | Roffi, 2017 [2] | 51 | 4 | Scaffolds for long bone defects with positive effects in animals but questionable effects in humans. |
| Bone fracture/defects | Cell therapy | Shanbhag, 2019 [3] | 57 | 47 | Cell therapy for bone regeneration with larger effect sizes in meta-analysis of animal studies compared to human trials. |
| Bone fracture/defects | Mesenchymal stem cells | Gundestrup, 2020 [4] | 4 | 2 | Mesenchymal stem cells for bone repair with beneficial effects in both animals and humans. |
| Osteoarthritis | Bone marrow concentrate | Cavallo, 2021 [5] | 4 | 18 | Different cell therapies (including modes of application) between animal and human studies for cartilage repair in osteoarthritis. |
| Osteoarthritis | Cox-2 inhibitors | Timur, 2020 [6] | 21 | 4 | Cox-2 inhibitors with chondroprotective effects in animals upon intra-articular application. Human studies did not investigate intraarticular application. In humans, systematic application without chondroprotective effects. |
| Osteoarthritis | Biosynthetic scaffolds | Veronesi, 2021 [7] | 46 | 30 | A variety of scaffolds have been tested in animals for osteoarthritis treatment but only two scaffolds have been tested in humans, with promising results. |
| Meniscal injury | Biological augmentation | Moran, 2015 [8] | 18 | 3 | Biological augmentation with beneficial effects in animal studies and few human studies. Study notes substantial differences in joint use between animals such as rodents or horsed and humans. |
| Meniscal injury | Stem cells | Rinonapoli, 2021 [9] | 13 | 5 | A variety of stem cell therapies with beneficial effects on meniscal defects in animal and human studies. |
| Spinal fusion | Electric stimulation | Cottrill, 2019 [10] | 17 | 16 | Electrical stimulation enhances spinal fusion in animals and humans, but there were higher effect sizes in animal studies. |
| Spinal fusion | Bioactive glasses | Cottrill, 2020 [11] | 12 | 12 | Bioactive glasses enhance spinal fusion in animals and humans. Surprisingly, there were higher effect sizes in humans in the meta-analysis. |
| Anterior cruciate ligament injury | Biological augmentation | Hexter, 2018 [12] | 92 | 20 | Anterior cruciate ligament reconstruction tested mostly in small animal models and short follow-up in animal studies. No meaningful clinical outcomes. |
| Anterior cruciate ligament injury | biological enhancement | Li, 2018 [13] | 11 | 7 | Discordant findings between preclinical and clinical studies. |
| Cartilage defects | Mesenchymal stem cells | Goldberg, 2017 [14] | 111 | 31 | A large heterogeneity in applied stem cell approaches for animal and human studies. |
| Soft-tissue injury | Low-intensity pulsed ultrasound | Lai, 2021 [15] | 28 | 2 | Ultrasound for tendon healing with a beneficial effect in animals but not in humans. |
| Disc degeneration | Cell therapy | Oehme, 2015 [16] | 42 | 4 | Cell therapy for lumbar disc degeneration with beneficial effects in both animals and humans. |
| Musculoskeletal Injuries | Vitamin C | DePhillipo, 2018 [17] | 7 | 3 | Vitamin C improves fracture healing in animal models but not in humans. |

The data underlying this table can be found on <https://osf.io/frjm4> (Sheet: *Curated*).

**References**

1. Roffi A, Di Matteo B, Krishnakumar GS, Kon E, Filardo G. Platelet-rich plasma for the treatment of bone defects: from pre-clinical rational to evidence in the clinical practice. A systematic review. International Orthopaedics. 2017;41(2):221-37. doi: 10.1007/s00264-016-3342-9. PubMed PMID: 27888295.

2. Roffi A, Krishnakumar GS, Gostynska N, Kon E, Candrian C, Filardo G. The Role of Three-Dimensional Scaffolds in Treating Long Bone Defects: Evidence from Preclinical and Clinical Literature-A Systematic Review. BioMed Research International. 2017;2017:8074178. doi: 10.1155/2017/8074178. PubMed PMID: 28852649.

3. Shanbhag S, Suliman S, Pandis N, Stavropoulos A, Sanz M, Mustafa K. Cell therapy for orofacial bone regeneration: A systematic review and meta-analysis. Journal of Clinical Periodontology. 2019;46 Suppl 21:162-82. doi: 10.1111/jcpe.13049. PubMed PMID: 30623455.

4. Gundestrup AK, Lynggaard CD, Forner L, Heino TJ, Jakobsen KK, Fischer-Nielsen A, et al. Mesenchymal Stem Cell Therapy for Osteoradionecrosis of the Mandible: a Systematic Review of Preclinical and Human Studies. Stem Cell Reviews & Reports. 2020;16(6):1208-21. doi: 10.1007/s12015-020-10034-5. PubMed PMID: 32869179.

5. Cavallo C, Boffa A, Andriolo L, Silva S, Grigolo B, Zaffagnini S, et al. Bone marrow concentrate injections for the treatment of osteoarthritis: evidence from preclinical findings to the clinical application. International Orthopaedics. 2021;45(2):525-38. doi: 10.1007/s00264-020-04703-w. PubMed PMID: 32661635.

6. Timur UT, Caron MMJ, Jeuken RM, Bastiaansen-Jenniskens YM, Welting TJM, van Rhijn LW, et al. Chondroprotective Actions of Selective COX-2 Inhibitors In Vivo: A Systematic Review. International Journal of Molecular Sciences. 2020;21(18):22. doi: 10.3390/ijms21186962. PubMed PMID: 32971951.

7. Veronesi F, Di Matteo B, Vitale ND, Filardo G, Visani A, Kon E, et al. Biosynthetic scaffolds for partial meniscal loss: A systematic review from animal models to clinical practice. Bioactive Materials. 2021;6(11):3782-800. doi: 10.1016/j.bioactmat.2021.03.033. PubMed PMID: 33898878.

8. Moran CJ, Busilacchi A, Lee CA, Athanasiou KA, Verdonk PC. Biological augmentation and tissue engineering approaches in meniscus surgery. Arthroscopy. 2015;31(5):944-55. doi: 10.1016/j.arthro.2014.11.044. PubMed PMID: 25687715.

9. Rinonapoli G, Gregori P, Di Matteo B, Impieri L, Ceccarini P, Manfreda F, et al. Stem cells application in meniscal tears: a systematic review of pre-clinical and clinical evidence. European Review for Medical & Pharmacological Sciences. 2021;25(24):7754-64. doi: 10.26355/eurrev_202112_27622. PubMed PMID: 34982437.

10. Cottrill E, Pennington Z, Ahmed AK, Lubelski D, Goodwin ML, Perdomo-Pantoja A, et al. The effect of electrical stimulation therapies on spinal fusion: a cross-disciplinary systematic review and meta-analysis of the preclinical and clinical data. Journal of Neurosurgery Spine. 2019:1-21. doi: 10.3171/2019.5.SPINE19465. PubMed PMID: 31593923.

11. Cottrill E, Pennington Z, Lankipalle N, Ehresman J, Valencia C, Schilling A, et al. The effect of bioactive glasses on spinal fusion: A cross-disciplinary systematic review and meta-analysis of the preclinical and clinical data. Journal of Clinical Neuroscience. 2020;78:34-46. doi: 10.1016/j.jocn.2020.04.035. PubMed PMID: 32331941.

12. Hexter AT, Thangarajah T, Blunn G, Haddad FS. Biological augmentation of graft healing in anterior cruciate ligament reconstruction: a systematic review. Bone & Joint Journal. 2018;100-B(3):271-84. doi: 10.1302/0301-620X.100B3.BJJ-2017-0733.R2. PubMed PMID: 29589505.

13. Li Y, Fu SC, Cheuk YC, Song G, Feng H, Yung SH. The non-reconstructive treatment of complete ACL tear with biological enhancement in clinical and preclinical studies: A systematic review. Asia-Pacific Journal of Sports Medicine, Arthroscopy, Rehabilitation and Technology. 2018;14:10-6. doi: 10.1016/j.asmart.2018.04.002.

14. Goldberg A, Mitchell K, Soans J, Kim L, Zaidi R. The use of mesenchymal stem cells for cartilage repair and regeneration: a systematic review. Journal of Orthopaedic Surgery. 2017;12(1):39. doi: 10.1186/s13018-017-0534-y. PubMed PMID: 28279182.

15. Lai WC, Iglesias BC, Mark BJ, Wang D. Low-Intensity Pulsed Ultrasound Augments Tendon, Ligament, and Bone-Soft Tissue Healing in Preclinical Animal Models: A Systematic Review. Arthroscopy. 2021;37(7):2318-33.e3. doi: 10.1016/j.arthro.2021.02.019. PubMed PMID: 33621647.

16. Oehme D, Goldschlager T, Ghosh P, Rosenfeld JV, Jenkin G. Cell-Based Therapies Used to Treat Lumbar Degenerative Disc Disease: A Systematic Review of Animal Studies and Human Clinical Trials. Stem Cells International. 2015;2015:946031. doi: 10.1155/2015/946031. PubMed PMID: 26074979.

17. DePhillipo NN, Aman ZS, Kennedy MI, Begley JP, Moatshe G, LaPrade RF. Efficacy of Vitamin C Supplementation on Collagen Synthesis and Oxidative Stress After Musculoskeletal Injuries: A Systematic Review. Orthopaedic Journal of Sports Medicine. 2018;6(10):2325967118804544. doi: 10.1177/2325967118804544. PubMed PMID: 30386805.
