# Supplementary material for: Analysis of animal-to-human translation shows that only 5% of animal-tested therapeutic interventions obtain regulatory approval for human applications
Source: PLoS Biol. 2024 Jun 13;22(6):e3002667. doi: 10.1371/journal.pbio.3002667 (PMC11175415; doi:10.1371/journal.pbio.3002667)
Supplement: S4 Table — (DOCX) [file pbio.3002667.s014.docx]

**Supplementary Table 4**: Translational assessment of interventions for psychiatric disorders.

| **Disease/condition** | **Intervention** | **Study** | **Animal studies** | **Human studies** | **Summary** |
| --- | --- | --- | --- | --- | --- |
| Addiction | Ovarian sex hormones | Arunogiri, 2021 [1] | 47 | 3 | Progesterone with consistent beneficial effect in animals, but less consistent in human trials. |
| Addiction | Cannabinoids | Daldegan, 2021 [2] | 15 | 10 | Cannabinoids with mostly protective effects in animal addiction models. Human studies more heterogenous with discrepant outcome measures compared to animal studies. |
| Addiction | Cannabinoids | Daldegan, 2021 [3] | 19 | 27 | Cannabidiol protected from cocaine abuse in animals but not in humans. |
| Psychiatric disorders | Cannabidiol | Calapai, 2019 [4] | 63 | 6 | Cannabidiol with beneficial effects in a variety of animal models of psychiatric diseases. Human evidence limited to schizophrenia and anxiety. |
| Psychiatric disorders | Fecal microbiota | Chinna, 2020 [5] | 20 | 8 | Both mouse and human fecal transplant from ill donors causes psychiatric symptoms in mice. |
| Psychiatric disorders | Alpha-7 nicotinic agonists | Lewis, 2017 [6] | 29 | 18 | Alpha-7 nicotinic agonists enhanced cognition with positive effects in animal studies but not in human trials (schizophrenia, Alzheimer’s disease) |
| Anxiety | Probiotics | Reis, 2018 [7] | 22 | 14 | Probiotics with mostly beneficial effects on anxiety in animal studies (12/22), but only few human trials with positive effect (3/14, > 1500 patients). Different outcome measures in animals and humans as well as time interval of drug application. |
| Anxiety | Probiotics | Wang, 2016 [8] | 25 | 15 | Probiotics with positive effects on anxiety in animals. Human evidence inconclusive. |
| Obsessive compulsive disorder | 5-HT3 antagonists | Serata, 2015 [9] | NA | NA | 5-HT3 blockers like ondansetron or granisetron with beneficial effects on OCD in humans. No animal OCD model tested directly implicated receptors. |

The data underlying this table can be found on <https://osf.io/frjm4> (Sheet: *Curated*).

**References**

1. Arunogiri S, Crossin R, Rizzo D, Walker L, Ridley K, Gurvich C. A systematic review of the effect of ovarian sex hormones on stimulant use in females. Addiction Biology. 2021;26(6):e13079. doi: 10.1111/adb.13079. PubMed PMID: 34374475.

2. Daldegan-Bueno D, Maia LO, Glass M, Jutras-Aswad D, Fischer B. Co-exposure of cannabinoids with amphetamines and biological, behavioural and health outcomes: a scoping review of animal and human studies. Psychopharmacology. 2022;239(5):1211-30. doi: 10.1007/s00213-021-05960-2. PubMed PMID: 34613429.

3. Daldegan-Bueno D, Maia LO, Glass M, Jutras-Aswad D, Fischer B. Co-exposure of cocaine and cannabinoids and its association with select biological, behavioural and health outcomes: A systematic scoping review of multi-disciplinary studies. European Neuropsychopharmacology. 2021;51:106-31. doi: 10.1016/j.euroneuro.2021.06.002. PubMed PMID: 34273801.

4. Calapai G, Mannucci C, Chinou I, Cardia L, Calapai F, Sorbara EE, et al. Preclinical and Clinical Evidence Supporting Use of Cannabidiol in Psychiatry. Evidence-based Complementary and Alternative Medicine. 2019;2019. doi: 10.1155/2019/2509129.

5. Chinna Meyyappan A, Forth E, Wallace CJK, Milev R. Effect of fecal microbiota transplant on symptoms of psychiatric disorders: A systematic review. BMC Psychiatry. 2020;20(1). doi: 10.1186/s12888-020-02654-5.

6. Lewis AS, van Schalkwyk GI, Bloch MH. Alpha-7 nicotinic agonists for cognitive deficits in neuropsychiatric disorders: A translational meta-analysis of rodent and human studies. Progress in Neuro-Psychopharmacology & Biological Psychiatry. 2017;75:45-53. doi: 10.1016/j.pnpbp.2017.01.001. PubMed PMID: 28065843.

7. Reis DJ, Ilardi SS, Punt SEW. The anxiolytic effect of probiotics: A systematic review and meta-analysis of the clinical and preclinical literature. PLoS ONE [Electronic Resource]. 2018;13(6):e0199041. doi: 10.1371/journal.pone.0199041. PubMed PMID: 29924822.

8. Wang H, Lee IS, Braun C, Enck P. Effect of Probiotics on Central Nervous System Functions in Animals and Humans: A Systematic Review. Journal of neurogastroenterology and motility. 2016;22(4):589-605. doi: 10.5056/jnm16018. PubMed PMID: 27413138.

9. Serata D, Kotzalidis GD, Rapinesi C, Janiri D, Di Pietro S, Callovini G, et al. Are 5-HT3 antagonists effective in obsessive-compulsive disorder? A systematic review of literature. Human Psychopharmacology. 2015;30(2):70-84. doi: 10.1002/hup.2461. PubMed PMID: 25676060.
