# Supplementary material for: Analysis of animal-to-human translation shows that only 5% of animal-tested therapeutic interventions obtain regulatory approval for human applications
Source: PLoS Biol. 2024 Jun 13;22(6):e3002667. doi: 10.1371/journal.pbio.3002667 (PMC11175415; doi:10.1371/journal.pbio.3002667)
Supplement: S5 Table — (DOCX) [file pbio.3002667.s015.docx]

**Supplementary Table 5**: Translational assessment of interventions for diseases of the circulatory system.

| **Disease/condition** | **Intervention** | **Study** | **Animal studies** | **Human studies** | **Summary** |
| --- | --- | --- | --- | --- | --- |
| Cardiac arrest | A plethora of drugs | Lind, 2021 [1] | 415 | 43 | 415 animal studies testing 190 pharmacological interventions. 43 clinical trials testing 26 different interventions Drugs for cardiac arrest: 1/3 of clinical trials positive. Some drugs tested in humans did not have (prior) positive animal studies. A large number of animal studies was performed after publication of a clinical trial. Study notes that the experimental animal scenario is highly different from the clinical scenario, i.e., a concern that animal studies lack the severity and characteristics of human studies. |
| Cardiac arrest | A plethora of drugs | Reynolds, 2007 [2] | 119 | NA | Drug administration for cardiac arrest with substantially earlier application in animals compared to humans (9.5 versus 19.4 minutes for animals versus humans). |
| Hypertension | Nigerian medicinal plants | Abdulazeez, 2021 [3] | 23 | 5 | Nigerian medicinal plants with positive effects on blood pressure in both animals and humans, but high heterogeneity in outcome measures for animal studies. |
| Atrial Fibrillation | Stereotactic Radiotherapy | Franzetti, 2022 [4] | 9 | 12 | Radiotherapy with beneficial effect on cardiac arrythmia in animals but only modest/unclear effects in human trials. Surprisingly, same minimal dose of radiation for animals and humans seem to be effective. |
| Cardiomyopathy | Cell therapy | Gho, 2013 [5] | 29 | 15 | Cell therapy with mostly positive effects on cardomytopathy in animals and humans. A lack of large animal model for cardiomytopathy is noted. |
| Acute myocaridal infarction | Shexiang Baoxin | Guo, 2021 [6] | 5 | 19 | Shexiang Baoxin Pill with positive effects on myocardial infarction in animals and humans. |
| Heart disease | Cell therapy | Martinez, 2020 [7] | 18 | 13 | Cell-based therapies for cardiac disease with efficacy in animal and human studies. |
| Cardiotoxicity | Physical exercise | Naaktgeboren, 2021 [8] | 37 | 3 | Exercise-mediated cardioprotection against chemotherapy with beneficial effects in animals but less clear evidence in humans. |
| Endovascular interventions | MRI-Guided Endovascular Arterial Interventions | Nijsink, 2022 [9] | 33 | 4 | MRI-guided endovascular intervention with complication rates slightly higher in humans compared to animals |
| Vascular grafts | Tissue-Engineered Vascular Grafts: | Skovrind, 2019 [10] | 68 | 3 | Non-clinically relevant outcomes and therapy regimens used in animal studies. |

The data underlying this table can be found on <https://osf.io/frjm4> (Sheet: *Curated*).

**References**

1. Lind PC, Johannsen CM, Vammen L, Magnussen A, Andersen LW, Granfeldt A. Translation from animal studies of novel pharmacological therapies to clinical trials in cardiac arrest: A systematic review. Resuscitation. 2021;158:258-69. doi: 10.1016/j.resuscitation.2020.10.028. PubMed PMID: 33147523.

2. Reynolds JC, Rittenberger JC, Menegazzi JJ. Drug administration in animal studies of cardiac arrest does not reflect human clinical experience. Resuscitation. 2007;74(1):13-26. doi: 10.1016/j.resuscitation.2006.10.032.

3. Abdulazeez MA, Muhammad SA, Saidu Y, Sallau AB, Arzai AA, Tabari MA, et al. A systematic review with meta-analysis on the antihypertensive efficacy of Nigerian medicinal plants. Journal of Ethnopharmacology. 2021;279:114342. doi: 10.1016/j.jep.2021.114342. PubMed PMID: 34157327.

4. Franzetti J, Volpe S, Catto V, Conte E, Piccolo C, Pepa M, et al. Stereotactic Radiotherapy Ablation and Atrial Fibrillation: Technical Issues and Clinical Expectations Derived From a Systematic Review. Frontiers in Cardiovascular Medicine. 2022;9:849201. doi: 10.3389/fcvm.2022.849201. PubMed PMID: 35592393.

5. Gho JM, Kummeling GJ, Koudstaal S, Jansen Of Lorkeers SJ, Doevendans PA, Asselbergs FW, et al. Cell therapy, a novel remedy for dilated cardiomyopathy? A systematic review. Journal of Cardiac Failure. 2013;19(7):494-502. doi: 10.1016/j.cardfail.2013.05.006. PubMed PMID: 23834925.

6. Guo J, Qin Z, He Q, Fong TL, Lau NC, Cho WCS, et al. Shexiang Baoxin Pill for Acute Myocardial Infarction: Clinical Evidence and Molecular Mechanism of Antioxidative Stress. Oxidative Medicine and Cellular Longevity. 2021;2021. doi: 10.1155/2021/7644648.

7. Martinez J, Zoretic S, Moreira A. Cell based therapies in congenital heart disease: A systematic review and meta-analysis. World Journal for Pediatric and Congenital Heart Surgery. 2020;11(2):NP40-NP1. doi: 10.1177/2150135120904324.

8. Naaktgeboren WR, Binyam D, Stuiver MM, Aaronson NK, Teske AJ, van Harten WH, et al. Efficacy of Physical Exercise to Offset Anthracycline-Induced Cardiotoxicity: A Systematic Review and Meta-Analysis of Clinical and Preclinical Studies. Journal of the American Heart Association. 2021;10(17):e021580. doi: 10.1161/JAHA.121.021580. PubMed PMID: 34472371.

9. Nijsink H, Overduin CG, Willems LH, Warle MC, Futterer JJ. Current State of MRI-Guided Endovascular Arterial Interventions: A Systematic Review of Preclinical and Clinical Studies. Journal of Magnetic Resonance Imaging. 2022;14:14. doi: 10.1002/jmri.28205. PubMed PMID: 35420239.

10. Skovrind I, Harvald EB, Juul Belling H, Jorgensen CD, Lindholt JS, Andersen DC. Concise Review: Patency of Small-Diameter Tissue-Engineered Vascular Grafts: A Meta-Analysis of Preclinical Trials. Stem Cells Translational Medicine. 2019;8(7):671-80. doi: 10.1002/sctm.18-0287. PubMed PMID: 30920771.
