# Supplementary material for: Analysis of animal-to-human translation shows that only 5% of animal-tested therapeutic interventions obtain regulatory approval for human applications
Source: PLoS Biol. 2024 Jun 13;22(6):e3002667. doi: 10.1371/journal.pbio.3002667 (PMC11175415; doi:10.1371/journal.pbio.3002667)
Supplement: S6 Table — (DOCX) [file pbio.3002667.s016.docx]

**Supplementary Table 6**: Translational assessment of interventions for diseases of the digestive system.

| **Disease/condition** | **Intervention** | **Study** | **Animal studies** | **Human studies** | **Summary** |
| --- | --- | --- | --- | --- | --- |
| Nutrition | Polyphenols | Alves, 2020 [1] | 22 | 2 | Prebiotics with beneficial effects in animals but human evidence unclear. |
| Dyslipidemia | Citrus extract | Carvalho, 2022 [2] | 23 | 4 | Citrus products with similar efficacy at improving blood fat levels and similar magnitudes for animal and human studies |
| Dental Implant Osseointegration | Magnetic stimulation | Cecoro, 2022 [3] | 18 | 6 | Magnetic stimulation of tooth implant osseointegration with high heterogeneity of methodological approaches between animal and human studies. |
| Liver failure | Bioartificial liver support systems | He, 2019 [4] | 12 | 18 | Bioartificial liver with beneficial effects in animals but not humans. Therapy only tested in older human studies. |
| Inflammatory bowel disease | Dipeptidyl peptidase 4 | Melo, 2021 [5] | 36 | 12 | Dipeptidyl Peptidase 4 for inflammatory bowel disease with promising animal data but only modest human efficacy. |
| Pancreatitis | Activated protein C | Miranda, 2012 [6] | 4 | 1 | Activated protein C for pancreatitis with positive effects in 3/4 of animal studies, one human RCT with negative results. |
| Inflammatory bowel disease | Resistant starches | Montroy, 2020 [7] | 21 | 7 | Resistant starches improved inflammatory bowel disease in animals and humans, although the evidence in humans is limited. |
| Fatty liver disease | Inositol | Pani, 2020 [8] | 10 | 1 | inositol improved fatty liver in animals, and also in 1 small human RCT. |
| Laparoscopic liver surgery | Image guided navigation in laparoscopic liver surgery | Schneider, 2021 [9] | 17 | 33 | Many *ex vivo* models limited surgical relevance for the clinical scenario. |

The data underlying this table can be found on <https://osf.io/frjm4> (Sheet: *Curated*).

**References**

1. Alves-Santos AM, Sugizaki CSA, Lima GC, Naves MMV. Prebiotic effect of dietary polyphenols: A systematic review. J Funct Food. 2020;74. doi: 10.1016/j.jff.2020.104169. PubMed PMID: WOS:000576946500004.

2. Carvalho BMR, Nascimento LC, Nascimento JC, Goncalves V, Ziegelmann PK, Tavares DS, et al. Citrus Extract as a Perspective for the Control of Dyslipidemia: A Systematic Review With Meta-Analysis From Animal Models to Human Studies. Frontiers in Pharmacology. 2022;13:822678. doi: 10.3389/fphar.2022.822678. PubMed PMID: 35237168.

3. Cecoro G, Bencivenga D, Annunziata M, Cennamo N, Della Ragione F, Formisano A, et al. Effects of Magnetic Stimulation on Dental Implant Osseointegration: A Scoping Review. Appl Sci-Basel. 2022;12(9). doi: 10.3390/app12094496. PubMed PMID: WOS:000794540500001.

4. He YT, Qi YN, Zhang BQ, Li JB, Bao J. Bioartificial liver support systems for acute liver failure: A systematic review and meta-analysis of the clinical and preclinical literature. World Journal of Gastroenterology. 2019;25(27):3634-48. doi: 10.3748/wjg.v25.i27.3634. PubMed PMID: 31367162.

5. Melo FJ, Pinto-Lopes P, Estevinho MM, Magro F. The Role of Dipeptidyl Peptidase 4 as a Therapeutic Target and Serum Biomarker in Inflammatory Bowel Disease: A Systematic Review. Inflammatory Bowel Diseases. 2021;27(7):1153-65. doi: 10.1093/ibd/izaa324. PubMed PMID: 33295607.

6. Miranda CJ, Babu BI, Siriwardena AK. Recombinant human activated protein C as a disease modifier in severe acute pancreatitis: systematic review of current evidence. Pancreatology. 2012;12(2):119-23. doi: 10.1016/j.pan.2012.01.002. PubMed PMID: 22487521.

7. Montroy J, Berjawi R, Lalu MM, Podolsky E, Peixoto C, Sahin L, et al. The effects of resistant starches on inflammatory bowel disease in preclinical and clinical settings: a systematic review and meta-analysis. BMC Gastroenterology. 2020;20(1):372. doi: 10.1186/s12876-020-01516-4. PubMed PMID: 33167889.

8. Pani A, Giossi R, Menichelli D, Fittipaldo VA, Agnelli F, Inglese E, et al. Inositol and Non-Alcoholic Fatty Liver Disease: A Systematic Review on Deficiencies and Supplementation. Nutrients. 2020;12(11):03. doi: 10.3390/nu12113379. PubMed PMID: 33153126.

9. Schneider C, Allam M, Stoyanov D, Hawkes DJ, Gurusamy K, Davidson BR. Performance of image guided navigation in laparoscopic liver surgery – A systematic review. Surgical Oncology. 2021;38. doi: 10.1016/j.suronc.2021.101637.
