# Supplementary material for: Analysis of animal-to-human translation shows that only 5% of animal-tested therapeutic interventions obtain regulatory approval for human applications
Source: PLoS Biol. 2024 Jun 13;22(6):e3002667. doi: 10.1371/journal.pbio.3002667 (PMC11175415; doi:10.1371/journal.pbio.3002667)
Supplement: S7 Table — (DOCX) [file pbio.3002667.s017.docx]

**Supplementary Table 7**: Translational assessment of interventions for cancer.

| **Disease/condition** | **Intervention** | **Study** | **Animal studies** | **Human studies** | **Summary** |
| --- | --- | --- | --- | --- | --- |
| Cancer | Therapeutic targeting of STAT3 pathway | Peisl, 2021 [1] | 25 | 9 | 20 therapies successfully tested in animals for pancreatic cancer, only 5 investigated in humans. Discrepancy might stem from human studies not investigating a certain molecular pathway |
| Cancer | Oncolytic virus talimogene laherparepvec | Lalu, 2019 [2] | 5 | 7 | Mapping development trajectory for oncolytic virus for cancer therapy. Effect sizes decreased from preclinical to clinical development (80-100% regression rates of tumours in animal experiments versus 0-24% in human studies, the more rigorous the trial, the smaller the effect size). Preclinical studies and less so clinical studies at high risk of bias. Authors discuss that even successful biotherapeutics may not demonstrate a clear translational road map, but we should be able to observe consistent and coherent patterns. Attention should be given to validities. Disease states and outcomes measured should have high construct validity. |
| Cancer | Nanoparticles use for delivering ursolic acid | Miatmoko, 2021 [3] | 15 | 3 | Nanoparticles use for delivering ursolic acid safe in animals and humans. No efficacy data available for humans (only phase 1 trials). |
| Cancer | liposomal versus conventional non-liposomal doxorubicin | Petersen, 2016 | 11 | 14 | In animal models, liposome formulation lead to increased survival compared to standard regimen chemotherapy. This contrasts with human studies in which no increased survival or lower progression rates were observed. High risk of bias for clinical and even more so for preclinical studies. Authors discuss potential reasons for failure: low construct validity (animal models with implanted tumours that grow rapidly within weeks compared to slowly growing tumours in humans), different dosing regimens and outcomes. |
| Glioblastoma | Glioblastoma drug therapies | Lyne, 2021 [4] | 115 | 35 | Drugs repurposed for glioblastoma show beneficial effects in animals but only modestly in humans. |
| Glioblastoma | Statins | Rendon, 2022 [5] | 12 | 14 | Statin use with beneficial effect in animal studies but not in clinical studies. |
| Leukemia | Plerixafor in combination with chemotherapy and/or hematopoietic cell transplantation | Maganti, 2020 [6] | 10 | 9 | Plerixafor as therapy for leukemia with promising data in animals and humans. |
| Gynecological cancer | Ibrutinib | Metzler, 2020 [7] | 9 | 3 | Ibrutinib in gynecological malignancies with different effects in animals and humans. Different tumor setting: Humans with end-stage cancer, animals with early-stage cancer. |
| Glioma | ketogenic or caloric restricted diets | Noorlag, 2019 [8] | 24 | 8 | Ketogenic or caloric restricted diets for glioma with a clear effect in animals but less clear for humans. |
| Meningioma | Mifepristone | Cossu, 2015 [9] | 7 | 6 | Mifepristone for meningioma with mostly positive effects in animal studies and mixed effects in clinical studies. |
| Brain tumours | Photon radiotherapy | Hart, 2022 [10] | 49 | 20 | Radiosurgery was tested under different conditions in animals and humans. The effective dose was different for animals and humans. |
| Bladder Cancer | Androgen signaling | Creta, 2021 [11] | 6 | 8 | Androgen signalling improves bladder cancer in animals and humans. |
| Lung cancer | Vitamin A and retinoid derivatives | Fritz, 2011 [12] | 67 | 63 | There was overall insufficient evidence to support the use of vitamin A or related retinoids for the treatment or prevention of lung cancers in animals and humans. |

The data underlying this table can be found on <https://osf.io/frjm4> (Sheet: *Curated*).

**References**

1. Peisl S, Mellenthin C, Vignot L, Gonelle-Gispert C, Buhler L, Egger B. Therapeutic targeting of STAT3 pathways in pancreatic adenocarcinoma: A systematic review of clinical and preclinical literature. PLoS ONE [Electronic Resource]. 2021;16(6):e0252397. doi: 10.1371/journal.pone.0252397. PubMed PMID: 34138876.

2. Lalu M, Leung GJ, Dong YY, Montroy J, Butler C, Auer RC, et al. Mapping the preclinical to clinical evidence and development trajectory of the oncolytic virus talimogene laherparepvec (T-VEC): a systematic review. BMJ Open. 2019;9(12):e029475. doi: 10.1136/bmjopen-2019-029475. PubMed PMID: 31796474.

3. Miatmoko A, Mianing EA, Sari R, Hendradi E. Nanoparticles use for Delivering Ursolic Acid in Cancer Therapy: A Scoping Review. Frontiers in Pharmacology. 2021;12. doi: 10.3389/fphar.2021.787226.

4. Lyne SB, Yamini B. An Alternative Pipeline for Glioblastoma Therapeutics: A Systematic Review of Drug Repurposing in Glioblastoma. Cancers. 2021;13(8). doi: 10.3390/cancers13081953. PubMed PMID: WOS:000643955700001.

5. Rendon LF, Tewarie IA, Cote DJ, Gabriel A, Smith TR, Broekman MLD, et al. Statins and Gliomas: A Systematic Review of the Preclinical Studies and Meta-Analysis of the Clinical Literature. Drugs. 2022;82(3):293-310. doi: 10.1007/s40265-021-01668-x.

6. Maganti H, Visram A, Shorr R, Fulcher J, Sabloff M, Allan DS. Plerixafor in combination with chemotherapy and/or hematopoietic cell transplantation to treat acute leukemia: A systematic review and metanalysis of preclinical and clinical studies. Leukemia Research. 2020;97:106442. doi: 10.1016/j.leukres.2020.106442. PubMed PMID: 32877869.

7. Metzler JM, Burla L, Fink D, Imesch P. Ibrutinib in Gynecological Malignancies and Breast Cancer: A Systematic Review. International Journal of Molecular Sciences. 2020;21(11):10. doi: 10.3390/ijms21114154. PubMed PMID: 32532074.

8. Noorlag L, De Vos FY, Kok A, Broekman MLD, Seute T, Robe PA, et al. Treatment of malignant gliomas with ketogenic or caloric restricted diets: A systematic review of preclinical and early clinical studies. Clin Nutr. 2019;38(5):1986-94. doi: 10.1016/j.clnu.2018.10.024. PubMed PMID: WOS:000492797600002.

9. Cossu G, Levivier M, Daniel RT, Messerer M. The Role of Mifepristone in Meningiomas Management: A Systematic Review of the Literature. BioMed Research International. 2015;2015:267831. doi: 10.1155/2015/267831. PubMed PMID: 26146614.

10. Hart E, Ode Z, Derieppe MPP, Groenink L, Heymans MW, Otten R, et al. Blood-brain barrier permeability following conventional photon radiotherapy - A systematic review and meta-analysis of clinical and preclinical studies. Clinical and Translational Radiation Oncology. 2022;35:44-55. doi: 10.1016/j.ctro.2022.04.013. PubMed PMID: WOS:000801228100003.

11. Creta M, Celentano G, Napolitano L, La Rocca R, Capece M, Califano G, et al. Inhibition of androgen signalling improves the outcomes of therapies for bladder cancer: Results from a systematic review of preclinical and clinical evidence and meta-analysis of clinical studies. Diagnostics. 2021;11(2). doi: 10.3390/diagnostics11020351.

12. Fritz H, Kennedy D, Fergusson D, Fernandes R, Doucette S, Cooley K, et al. Vitamin A and retinoid derivatives for lung cancer: a systematic review and meta analysis. PLoS ONE [Electronic Resource]. 2011;6(6):e21107. doi: 10.1371/journal.pone.0021107. PubMed PMID: 21738614.
