# Supplementary material for: Analysis of animal-to-human translation shows that only 5% of animal-tested therapeutic interventions obtain regulatory approval for human applications
Source: PLoS Biol. 2024 Jun 13;22(6):e3002667. doi: 10.1371/journal.pbio.3002667 (PMC11175415; doi:10.1371/journal.pbio.3002667)
Supplement: S8 Table — (DOCX) [file pbio.3002667.s018.docx]

**Supplementary Table 8**: Translational assessment of interventions for other disorders/conditions.

| **Disease/condition** | **Intervention** | **Study** | **Animal studies** | **Human studies** | **Summary** |
| --- | --- | --- | --- | --- | --- |
| Diabetes mellitus | Indian ginseng | Durg, 2020 [1] | 13 | 5 | Indian ginseng corrects blood fat levels in animal and humans. |
| Diabetes mellitus | Germinated brown rice | Imam, 2012 [2] | 12 | 6 | Germinated brown rice with positive effects in diabetes and models thereof but long-term human studies are missing. |
| Diabetes mellitus | Exercise | Verboven, 2019 [3] | 8 | 13 | Exercise with mostly beneficial effect on blood pressure in diabetic animals. In diabetes patients, only improvement of diastolic but not systolic blood pressure. Different experimental approaches between animal and human studies. |
| Obesity | Green tea | Macedo, 2022 [4] | 23 | 6 | Green tea controlled obese lipids in animals but only marginally in humans. |
| Obesity | Aerobic exercise | Porflitt, 2022 [5] | 10 | 9 | Exercise mediates blood biomarker levels in animals and humans. |
| Glucose Metabolism | Microalgae Arthrospira | Ghanbari, 2021 [6] | 27 | 7 | Arthrospira with beneficial effect on blood glucose and fat in both animals and humans. |
| Chemical burns | Diphoterine | Alexander, 2018 [7] | 7 | 5 | Diphoterine was beneficial for chemical burns in animals and humans, but poor reporting and methodological quality was noted for animal and human studies. |
| Wound therapy | Negative-pressure wound therapy | Glass, 2017 [8] | 10 | 10 | Negative pressure in wounds with heterogenous efficacy data in animals and humans. |
| Burn wounds | Stem cells | Henriksen, 2020 [9] | 21 | 3 | Mesenchymal stem cells improved wound healing in animals and humans. However, animal studies were not cited in clinical studies. |
| Wound infection | Topical agents | Schwarzer, 2020 [10]sch | 5 | 3 | Topical agents for wound therapy with scarcity of animal and human studies. |
| Psoriasis | Sphingosine-1-Phosphate | Liu, 2021 [11] | 9 | 1 | S1P inhibitors beneficial for psoriasis in animals, 1 human study also beneficial. |
| Bladder/uretrha reconstruction | Bladder/Urethra reconstruction | Sloff, 2014 [12] | 28 | 2 | Tissue engineering for bladder reconstrution promising in animals but less in humans. |
| Bladder/uretrha reconstruction | Bladder/Urethra reconstruction | Versteegden, 2017 [13] | 80 | 23 | Reconstructive surgery beneficial in both animals and humans. Higher rates of side effects in human studies compared to animal studies. |
| Preterm infants | Postnatal nutrition | Hortensius, 2019 [14] | 24 | 22 | Nutritional therapy improved brain development in animal studies, but human RCTs without convincing effects. |
| Endometriosis | Toxic animal-based medicinal materials | Hwang, 2021 [15] | 8 | 12 | Such materials with mostly positive effects in animals and humans. |
| Respiratory outcomes in neonates | Sustained inflation | Lambert, 2020 [16] | 17 | 17 | Sustained inflation beneficial in animals but not in humans. |
| Inhalation trauma | Inhaled anticoagulation | Miller, 2014 [17] | 18 | 5 | Inhaled anticoagulation for smoke trauma with positive effects in animals and preliminary human studies. |
| Acute respiratory distress syndrome | Ventilation with lower tidal volumes | Serpa Neto, 2014 [18] | 25 | 6 | Clear association between tidal volume size and lung injury in both animal and human studies |
| Surgical animal models | Surgical procedures | Ruan, 2020 [19] | 411 | NA | Translation assessed by analyzing the frequency of citation in human studies over ten years following publication Low translation of surgical animal studies to humans (15%). Only 21% of animal study citations were in clinical trials. Median number of citations in human studies was 1 (IQR 0-5) (8 for other animal studies). |
| Conditioning, hormonal response | Conditioned hormonal responses | Skvortsova, 2019 [20] | 26 | 8 | Conditioned hormonal responses in general similar effects in animals and humans but more null studies in humans. |
| Cell transplantation | Mesenchymal Stromal Cells | Sanchez, 2021 [21] | 104 | 5 | Similar biodistribution of stem cells in animals and humans. |
| Conglomeration of human diseases | 11beta-hydroxysteroid dehydrogenase type 1 inhibitor | Gregory, 2020 [22] | NA | 28 | Animal data heterogenous. One phase 2 trial reduced HbA1c in diabetes, but trials in metabolic syndrome or Alzheimer’s disease have not met primary endpoints. |

The data underlying this table can be found on <https://osf.io/frjm4> (Sheet: *Curated*).

**References**

1. Durg S, Bavage S, Shivaram SB. Withania somnifera (Indian ginseng) in diabetes mellitus: A systematic review and meta-analysis of scientific evidence from experimental research to clinical application. Phytotherapy Research. 2020;34(5):1041-59. doi: 10.1002/ptr.6589. PubMed PMID: 31975514.

2. Imam MU, Azmi NH, Bhanger MI, Ismail N, Ismail M. Antidiabetic properties of germinated brown rice: a systematic review. Evidence-Based Complementary & Alternative Medicine: eCAM. 2012;2012:816501. doi: 10.1155/2012/816501. PubMed PMID: 23304216.

3. Verboven M, Van Ryckeghem L, Belkhouribchia J, Dendale P, Eijnde BO, Hansen D, et al. Effect of Exercise Intervention on Cardiac Function in Type 2 Diabetes Mellitus: A Systematic Review. Sports Medicine. 2019;49(2):255-68. doi: 10.1007/s40279-018-1003-4. PubMed PMID: 30357657.

4. Macedo APA, Goncalves MDS, Barreto Medeiros JM, David JM, Villarreal CF, Macambira SG, et al. Potential therapeutic effects of green tea on obese lipid profile - a systematic review. Nutrition & Health. 2022:2601060211073236. doi: 10.1177/02601060211073236. PubMed PMID: 35014893.

5. Porflitt-Rodriguez M, Guzman-Arriagada V, Sandoval-Valderrama R, Tam CS, Pavicic F, Ehrenfeld P, et al. Effects of aerobic exercise on fibroblast growth factor 21 in overweight and obesity. A systematic review. Metabolism: Clinical & Experimental. 2022;129:155137. doi: 10.1016/j.metabol.2022.155137. PubMed PMID: 35038422.

6. Ghanbari F, Amerizadeh A, Behshood P, Moradi S, Asgary S. Effect of Microalgae Arthrospira on Biomarkers of Glycemic Control and Glucose Metabolism: A Systematic Review and Meta-analysis. Current Problems in Cardiology. 2021:100942. doi: 10.1016/j.cpcardiol.2021.100942. PubMed PMID: 34538515.

7. Alexander KS, Wasiak J, Cleland H. Chemical burns: Diphoterine untangled. Burns. 2018;44(4):752-66. doi: 10.1016/j.burns.2017.09.017. PubMed PMID: 29029860.

8. Glass GE, Murphy GRF, Nanchahal J. Does negative-pressure wound therapy influence subjacent bacterial growth? A systematic review. Journal of Plastic, Reconstructive & Aesthetic Surgery: JPRAS. 2017;70(8):1028-37. doi: 10.1016/j.bjps.2017.05.027. PubMed PMID: 28602266.

9. Henriksen JL, Sorensen NB, Fink T, Zachar V, Porsborg SR. Systematic Review of Stem-Cell-Based Therapy of Burn Wounds: Lessons Learned from Animal and Clinical Studies. Cells. 2020;9(12):26. doi: 10.3390/cells9122545. PubMed PMID: 33256038.

10. Schwarzer S, James GA, Goeres D, Bjarnsholt T, Vickery K, Percival SL, et al. The efficacy of topical agents used in wounds for managing chronic biofilm infections: A systematic review. Journal of Infection. 2020;80(3):261-70. doi: 10.1016/j.jinf.2019.12.017. PubMed PMID: 31899281.

11. Liu L, Wang J, Li HJ, Zhang S, Jin MZ, Chen ST, et al. Sphingosine-1-Phosphate and Its Signal Modulators Alleviate Psoriasis-Like Dermatitis: Preclinical and Clinical Evidence and Possible Mechanisms. Frontiers in Immunology. 2021;12:759276. doi: 10.3389/fimmu.2021.759276. PubMed PMID: 34992595.

12. Sloff M, Simaioforidis V, de Vries R, Oosterwijk E, Feitz W. Tissue engineering of the bladder--reality or myth? A systematic review. Journal of Urology. 2014;192(4):1035-42. doi: 10.1016/j.juro.2014.03.116. PubMed PMID: 24769032.

13. Versteegden LRM, de Jonge P, IntHout J, van Kuppevelt TH, Oosterwijk E, Feitz WFJ, et al. Tissue Engineering of the Urethra: A Systematic Review and Meta-analysis of Preclinical and Clinical Studies. European Urology. 2017;72(4):594-606. doi: 10.1016/j.eururo.2017.03.026. PubMed PMID: 28385451.

14. Hortensius LM, van Elburg RM, Nijboer CH, Benders M, de Theije CGM. Postnatal Nutrition to Improve Brain Development in the Preterm Infant: A Systematic Review From Bench to Bedside. Frontiers in Physiology. 2019;10:961. doi: 10.3389/fphys.2019.00961. PubMed PMID: 31404162.

15. Hwang SI, Yoon YJ, Sung SH, Ha KT, Park JK. Toxic Animal-Based Medicinal Materials Can Be Effective in Treating Endometriosis: A Scoping Review. Toxins. 2021;13(2):14. doi: 10.3390/toxins13020145. PubMed PMID: 33673020.

16. Lambert CJ, Hooper SB, Te Pas AB, McGillick EV. Improving Newborn Respiratory Outcomes With a Sustained Inflation: A Systematic Narrative Review of Factors Regulating Outcome in Animal and Clinical Studies. Frontiers in Pediatrics. 2020;8:516698. doi: 10.3389/fped.2020.516698. PubMed PMID: 33194881.

17. Miller AC, Elamin EM, Suffredini AF. Inhaled anticoagulation regimens for the treatment of smoke inhalation-associated acute lung injury: a systematic review. Critical Care Medicine. 2014;42(2):413-9. doi: 10.1097/CCM.0b013e3182a645e5. PubMed PMID: 24158173.

18. Serpa Neto A, Nagtzaam L, Schultz MJ. Ventilation with lower tidal volumes for critically ill patients without the acute respiratory distress syndrome: a systematic translational review and meta-analysis. Current Opinion in Critical Care. 2014;20(1):25-32. doi: 10.1097/MCC.0000000000000044. PubMed PMID: 24275571.

19. Ruan Y, Robinson NB, Khan FM, Hameed I, Rahouma M, Naik A, et al. The translation of surgical animal models to human clinical research: A cross-sectional study. International Journal of Surgery. 2020;77:25-9. doi: 10.1016/j.ijsu.2020.03.023.

20. Skvortsova A, Veldhuijzen DS, Kloosterman IEM, Meijer OC, van Middendorp H, Pacheco-Lopez G, et al. Conditioned hormonal responses: A systematic review in animals and humans. Frontiers in Neuroendocrinology. 2019;52:206-18. doi: 10.1016/j.yfrne.2018.12.005. PubMed PMID: 30590067.

21. Sanchez-Diaz M, Quinones-Vico MI, Sanabria de la Torre R, Montero-Vilchez T, Sierra-Sanchez A, Molina-Leyva A, et al. Biodistribution of Mesenchymal Stromal Cells after Administration in Animal Models and Humans: A Systematic Review. Journal of Clinical Medicine. 2021;10(13):29. doi: 10.3390/jcm10132925. PubMed PMID: 34210026.

22. Gregory S, Hill D, Grey B, Ketelbey W, Miller T, Muniz-Terrera G, et al. 11beta-hydroxysteroid dehydrogenase type 1 inhibitor use in human disease-a systematic review and narrative synthesis. Metabolism: Clinical & Experimental. 2020;108:154246. doi: 10.1016/j.metabol.2020.154246. PubMed PMID: 32333937.
