# Supplementary material for: Analysis of animal-to-human translation shows that only 5% of animal-tested therapeutic interventions obtain regulatory approval for human applications
Source: PLoS Biol. 2024 Jun 13;22(6):e3002667. doi: 10.1371/journal.pbio.3002667 (PMC11175415; doi:10.1371/journal.pbio.3002667)
Supplement: S9 Table — (DOCX) [file pbio.3002667.s019.docx]

**Supplementary Table 9:** Number of therapeutic interventions tested in animals, entering any clinical trial, any RCT, and eventually obtaining regulatory approval per disease/condition (n=54).

| **Disease** | **Number of reviews** | **Number of therapeutic interventions:** | | | | | | |
| --- | --- | --- | --- | --- | --- | --- | --- | --- |
|  |  | **Tested** | **In any human trial** | **%** | **In any RCT** | **%** | **With (FDA) approval** | **%** |
| Cardiac arrest | 2 | 151 | 13 | 9 | 10 | 7 | 0 | 0 |
| Multiple sclerosis | 5 | 34 | 31 | 91 | 28 | 82 | 7 | 21 |
| Stroke | 8 | 31 | 30 | 97 | 29 | 94 | 0 | 0 |
| Hypertension | 1 | 20 | 2 | 10 | 0 | 0 | 0 | 0 |
| Cancer | 1 | 12 | 5 | 42 | 5 | 42 | 3 | 25 |
| Dementia | 1 | 11 | 4 | 36 | 1 | 9 | 0 | 0 |
| Subarachnoid hemorrhage | 1 | 6 | 6 | 100 | 6 | 100 | 1 | 17 |
| Glioma/glioblastoma | 3 | 5 | 4 | 80 | 1 | 20 | 0 | 0 |
| Bladder cancer | 1 | 5 | 0 | 0 | 0 | 0 | 0 | 0 |
| Alzheimer’s disease | 3 | 4 | 4 | 100 | 2 | 50 | 0 | 0 |
| Psoriasis | 1 | 4 | 0 | 0 | 0 | 0 | 0 | 0 |
| Addiction | 3 | 3 | 3 | 100 | 2 | 67 | 0 | 0 |
| Osteoarthritis | 3 | 3 | 3 | 100 | 1 | 33 | 0 | 0 |
| Traumatic brain injury | 3 | 3 | 2 | 67 | 2 | 67 | 0 | 0 |
| Spinal cord injury | 1 | 3 | 3 | 100 | 3 | 100 | 0 | 0 |
| Diabetes | 3 | 2 | 2 | 100 | 2 | 100 | 0 | 0 |
| Pain | 2 | 2 | 2 | 100 | 2 | 100 | 1 | 50 |
| Parkinson’s disease | 2 | 2 | 2 | 100 | 2 | 100 | 0 | 0 |
| Spinal fusion surgery | 2 | 2 | 2 | 100 | 2 | 100 | 2 | 100 |
| Chemical burns | 2 | 2 | 2 | 100 | 1 | 50 | 0 | 0 |
| Epilepsy | 3 | 2 | 2 | 100 | 1 | 50 | 0 | 0 |
| ACL injury | 2 | 2 | 2 | 100 | 1 | 50 | 0 | 0 |
| Obesity | 2 | 2 | 2 | 100 | 2 | 100 | 0 | 0 |
| Degenerative disc disease | 1 | 2 | 2 | 100 | 0 | 0 | 0 | 0 |
| Bone regeneration | 2 | 2 | 2 | 100 | 2 | 100 | 0 | 0 |
| Anxiety | 2 | 1 | 1 | 100 | 1 | 100 | 0 | 0 |
| Inflammatory bowel disease | 2 | 1 | 1 | 100 | 1 | 100 | 0 | 0 |
| Intracerebral hemorrhage | 1 | 1 | 1 | 100 | 0 | 0 | 0 | 0 |
| Dyslipidemia | 1 | 1 | 1 | 100 | 1 | 100 | 0 | 0 |
| Meningioma | 1 | 1 | 1 | 100 | 1 | 100 | 0 | 0 |
| Musculoskeletal Injuries | 1 | 1 | 1 | 100 | 1 | 100 | 0 | 0 |
| Atrial fibrillation | 1 | 1 | 1 | 100 | 0 | 0 | 0 | 0 |
| Cardiomyopathy | 1 | 1 | 1 | 100 | 1 | 100 | 0 | 0 |
| Cartilage defects | 1 | 1 | 1 | 100 | 1 | 100 | 0 | 0 |
| Head and neck cancer | 1 | 1 | 1 | 100 | 0 | 0 | 0 | 0 |
| Acute myocaridal infarction | 1 | 1 | 1 | 100 | 1 | 100 | 0 | 0 |
| Liver failure | 1 | 1 | 1 | 100 | 1 | 100 | 0 | 0 |
| Endometriosis | 1 | 1 | 1 | 100 | 1 | 100 | 0 | 0 |
| Soft-tissue injury | 1 | 1 | 1 | 100 | 1 | 100 | 0 | 0 |
| Gynecological cancer | 1 | 1 | 1 | 100 | 1 | 100 | 0 | 0 |
| Inhalation trauma | 1 | 1 | 1 | 100 | 0 | 0 | 0 | 0 |
| Pancreatitis | 1 | 1 | 1 | 100 | 1 | 100 | 0 | 0 |
| Cardiotoxicity | 1 | 1 | 1 | 100 | 1 | 100 | 0 | 0 |
| Fatty liver disease | 1 | 1 | 1 | 100 | 1 | 100 | 0 | 0 |
| Meniscal tears | 1 | 1 | 1 | 100 | 1 | 100 | 0 | 0 |
| ARDS | 1 | 1 | 1 | 100 | 1 | 100 | 0 | 0 |
| Laparoscopic liver surgery | 1 | 1 | 1 | 100 | 0 | 0 | 0 | 0 |
| Lung cancer | 1 | 1 | 0 | 0 | 0 | 0 | 0 | 0 |
| Leukemia | 1 | 1 | 0 | 0 | 0 | 0 | 0 | 0 |
| ALS | 1 | 1 | 0 | 0 | 0 | 0 | 0 | 0 |
| Wound infection | 1 | 1 | 0 | 0 | 0 | 0 | 0 | 0 |
| OCD | 1 | 1 | 0 | 0 | 0 | 0 | 0 | 0 |
| Effects of brain radiation | 1 | 1 | 0 | 0 | 0 | 0 | 0 | 0 |
| Cell transplantation | 1 | 1 | 0 | 0 | 0 | 0 | 0 | 0 |

The data underlying this table can be found on <https://osf.io/frjm4> (Sheet: *Diseases*).
