# Supplementary material for: Analysis of animal-to-human translation shows that only 5% of animal-tested therapeutic interventions obtain regulatory approval for human applications
Source: PLoS Biol. 2024 Jun 13;22(6):e3002667. doi: 10.1371/journal.pbio.3002667 (PMC11175415; doi:10.1371/journal.pbio.3002667)
Supplement: Attachment — Submitted filename: Response_to_reviewers_R1.docx [file pbio.3002667.s021.docx]

**Point-by-point response**

Copied from the decision letter, responses in blue.

**Comments made by the Editor**

a) Please comply with PLOS' Data Policy (https://journals.plos.org/plosbiology/s/data-availability); specifically, we need you to supply the numerical values underlying Figs 2. 4ABC, 5, S2ABC, S3, S4, S5, S6, S7, S8, either as a supplementary data file or as a permanent DOI’d deposition. I note that your study is already preregistered with OSF (https://osf.io/jh2d8), so perhaps you could deposit it there?

We thank you, Dr. Roberts, for editing and reviewing our manuscript. All data and code underlying this study have been made available on our OSF page. Accordingly, we have revised our data availability statement which now reads:

“All data and code that support the findings of this study are available at <https://osf.io/frjm4> (data including meta-data) and <https://osf.io/9fgru> (R code). For any questions regarding the data, meta-data, or analysis code, contact the corresponding author, BVI.”

b) Please cite the location of the data clearly in all relevant main and supplementary Figure legends, e.g. “The data underlying this Figure can be found in S1 Data” or “The data underlying this Figure can be found in https://doi.org/10.5281/zenodo.XXXXX”

We are now citing for every figure and table which underlying data and code has been used to produce the respective figures and tables.

c) Please make any custom code available, either as a supplementary file or as part of your data deposition.

See above.

d) Your Data Availability Statement currently says “All data and code that support the findings of this study are available from the corresponding author, BVI, upon reasonable request, at benjamin.ineichen@uzh.ch” - this is not compliant with PLOS Data Policy, so please comply and update (see above).

See above.

e) Your Abstract is currently structured; please change it to an unstructured format (https://journals.plos.org/plosbiology/s/submission-guidelines#loc-abstract).

We changed the abstract to an unstructured format.

f) Please remove the beginning and end of your Title, to make it simply "Evaluating the success rate of biomedical therapies' journey from animals to humans"

We revised the title according to your suggestion.

**Reviewer #1:**

[identifies himself as Jan-Bas Prins]

This manuscript describes a systematic review of published systematic reviews or scoping reviews with or without meta-analysis which investigate translation of interventions in animal models of human diseases. The authors name their approach an umbrella review. The study adds a dimension to the ongoing debate about the validity and translatability of pre-clinical testing in drug development for humans. Focussing on three phases in the trajectory of drug testing: the pre-clinical phase, the randomised clinical trial phase and approval by the competent authority, in this case the FDA. The authors used published search strategies and a purposely developed one to mine Medline, Embase, and Web of Science. They performed a risk of bias analysis in which they identified several studies with some concerns others with high risk of bias. They started with 3343 records for the first screening and ended with 122 studies for the qualitative synthesis and 62 for the quantitative

synthesis/ meta-analysis. The study shows that although animal studies appear to translate better to humans than previously thought, there is still a very low rate of final approval. The manuscript is well-written and demonstrates sound research design, execution, and reporting.

We thank you, Dr. Prins, for reviewing our manuscript and for your positive and constructive comments.

I have only a few remarks and comments to make in order of appearance in the manuscript.

1. Material and methods:

a. Lines 74-76 - here it reads that Medline was searched with Ovid Medline interface. In other parts of the manuscript, reference is made to Pubmed, which is an interface to search Medline as well as additional biomedical content.

Thank you for pointing this out. We did in fact search Medline via the Ovid interface. We corrected this instance on line 147 and in Figure 1 (study flow chart).

b. Line 79 - typo 'SYRLCE'

We corrected this typo.

c. Line 81-84 - a 'broad' and a 'narrow' search strategy were tested for sensitivity before selecting one to the two. A threshold was set at 5% additional studies retrieved with the broad strategy. The authors 'did not'. It would be nice to know what the actual percentage was.

We clarified this, it now reads:

“However, we did not identify any additional eligible studies using the broad search strategy (i.e., 0%) , and so we used the narrower search string for this systematic review (see protocol for details).” (lines 91-92)

d. Line 96-97 - non-systematic reviews were excluded but retained for additional references. Only one additional article was found and added to the search results. This low number reflects the quality of the SRs included and could be discussed briefly.

The study flow chart presented in Figure 1 refers to the selection of systematic reviews, not to the primary studies. We clarified this in the Figure caption:

“Flow chart for inclusion of systematic reviews or scoping reviews with or without meta-analysis which investigate translation of interventions in animal models of human diseases.” (Page 21)

e. Line 106-107 - add reference (or URL) for Universal Desktop Ruler.

We added a proper citation including the URL for the Universal Desktop Ruler (line 117).

f. Line 151 - add references (or URLs) to the packages survminer and survival respectively.

We have incorporated Research Resource Identifiers (RRIDs) for the two R packages: survminer (RRID: SCR_021094) and survival (RRID: SCR_021137). This inclusion enables a precise and unique identification of each R package (lines 168 and 169).

2. Results:

a. Lines 181-185 - It is unclear if studies that were labelled as at high risk of bias or had concerns raised were still included in the analysis and if so, what the potential impact is.

We included all systematic reviews into our final analysis. Given that most reviews were deemed at low risk of bias, we expected no impact on our overall conclusions from including reviews across the spectrum of bias risk. We now specify this in the methods section:

“Of note, we included all systematic reviews into our final analysis, regardless of their risk of bias.” (lines 131-132)

And in the results section:

“Given that most included systematic reviews were deemed at low risk of bias, we anticipated no relevant impact on our overall conclusions.” (lines 201-202)

b. Lines 209-210 - the authors note 'that many animal studies were conducted subsequent to the publication of a corresponding clinical study'. Without the studies at hand, this can be interpreted in different ways. It would be helpful if the authors could elaborate on this notion by adding information to what extent the research questions of the animal studies were overlapping with or novel with respect to the clinical study.

We agree with you that this is an important issue. This statement is informed by the findings presented in the study by Lind and colleagues (https://doi.org/10.1016/j.resuscitation.2020.10.028). To clarify, we have referenced this study once more following the statement to explicitly indicate its connection. Unfortunately, the study does not quantify the scope of post-approval animal research or delve into the rationales behind conducting such animal studies.

It might be of interest to you that in one of our currently ongoing studies (https://osf.io/sjnym/), we observe that 90% of animal experiments were conducted after first-in-human trials and 90% even after official regulatory approval in multiple sclerosis drug development. As you already point out, there may of course be valid reasons for conducting animal experiments post initial human trials or even post-approval, such as seeking deeper understanding of a drug's mechanisms of action. However, the large volume of post-approval animal studies indicates a disconnect between preclinical and clinical research which we already point out as one hurdle for successful bench-to-bedside translation in the discussion (lines 314-316).

c. Line 217 - (281 therapies, Figure 3) add A to figure 3.

We now specify the panel of Figure 3 on line 238.

d. Line 230 - reference to Supplementary figure 2 - in this figure the term 'survival time' is used. Could the alternative 'lag time' be considered?

We have revised the titles and captions of Figure 5 and Supplementary Figure 2 to use the term "lag time," which we believe more accurately reflects the data presented.

e. Line 235-236 - what is the justification for the cut-off of less than 5 published animal studies?

Thank you for pointing this out. We wanted to exclude therapies which were only tested in very few animal experiments to denoise the data set. Based on this, we made the arbitrary choice to focus on therapies documented in at least 5 published animal studies. However, we find similarly high concordance rates when thresholding at 3 published animal studies. We have now detailed our methodology for setting this threshold in the methods section:

“To reduce noise of the dataset, we restricted our analysis to therapies that were the subject of five or more published animal studies.” (lines 159-161)

f. Lines 236-239 - The numbers in Table 2 are different from those in the text. The legend of table 2 should include information on the cut-off number of published animal studies.

Thank you for pointing this out. We corrected the numbers in the text:

“In evaluating concordance, we restricted our analysis to therapies that were the subject of five or more published animal studies, encompassing 62 therapies. These therapies were examined across 1496 animal studies, 515 clinical studies, and 220 RCTs. Out of these, positive outcomes were identified in 1181 (79%) animal studies, 317 (61%) clinical studies, and 111 (50%) RCTs (**Table 2**).” (page 10, lines 259-262)

We also added the cut-off of five published animal studies to the caption of Table 2 (page 28).

3. Discussion:

a. Lines 243-246 - although it may be considered as beyond the scope of the manuscript, the paper would gain from discussion on the differences between the therapies (and/ or models used) for neurological diseases and circulatory system diseases versus those for diseases with lower concordance. Also, in relation to the section of the discussion (lines 273-280) where the example of field of clinical neurology is presented to demonstrate the limited official approval of therapies while the results of animal studies and early clinical trials were promising.

We did not perform a detailed analysis of the differences across animal models, therapy categories, their mechanisms of action, or other related parameters according to disease categories (for instance, comparing neurological diseases to those with lower consistency between animals and humans, such as mental health disorders). However, we have included a section in the discussion to encourage future research to address this important issue:

“We did not systematically assess differences in animal models or therapy parameters such as therapy type (drug versus non-drug) for different disease categories—for instance, comparing conditions where animal and human therapy outcomes show high consistently (such as neurological or circulatory system diseases) against those with lower consistency (such as mental health issues or cancer). Future research could explore these factors as potential moderators on the alignment of results between animal studies and human applications.“ (Lines 340-345).

b. Lines 291-292 - the fourth potential hurdle for successful translation is the quality of human studies i.e. underpowered and inappropriate outcome measures. These refer to serious flaws in experimental design and outcomes of assessment of protocols by competent authorities. Could the authors add examples or provide some level of quantification?

We extended the discussion on this topic and included additional references:

“… [50]. For instance, early phase clinical trials testing interventions for neurological diseases are commonly underpowered [51]. Similarly, clinical trials may use trial outcomes not genuinely reflecting real-world patient settings such as complex composite outcomes, commonly seen in trials of cardiovascular diseases [52] or assessing cognitive domains in dementia trials not relevant for patients [53].” (lines 318-322).

References.

50.	Heneghan C, Goldacre B, Mahtani KR. Why clinical trial outcomes fail to translate into benefits for patients. Trials. 2017;18:1-7.

51.	Khan A, Fahl Mar K, Brown WA. The Impact of Underpowered Studies on Clinical Trial Results. Am J Psychiatry. 2018;175(2):188. doi: 10.1176/appi.ajp.2017.17091016. PubMed PMID: 29385825.

52.	Ferreira-González I, Permanyer-Miralda G, Domingo-Salvany A, Busse JW, Heels-Ansdell D, Montori VM, et al. Problems with use of composite end points in cardiovascular trials: systematic review of randomised controlled trials. Bmj. 2007;334(7597):786.

53.	Molnar FJ, Man‐Son‐Hing M, Fergusson D. Systematic review of measures of clinical significance employed in randomized controlled trials of drugs for dementia. Journal of the American Geriatrics Society. 2009;57(3):536-46.

c. Lines 312-314 - I am not sure I understand this limitation since only one publication was added to those already identified by the SRs based on the excluded papers.

This limitation addresses the reliance on published systematic reviews for identifying therapies that successfully transition from animal models to human applications, potentially omitting therapies that have made such transitions but have not been included in systematic reviews and thus being not included in our analysis. We have rephrased this limitation for clarity, as follows:

“First and most importantly, the therapies herein included were identified through systematic reviews specifically focusing on translation which will likely bias the factual translation. Such reviews may be more commonly conducted in fields where at least some therapies have already achieved clinical translation.” (lines 351-354).

d. Lines 318-320 - Is this a limitation?

We removed this as limitation of our study.

**Reviewer #2:**

[identifies himself as Jens Minnerup]

This is an excellent review article by leading experts in the field. The article will be of interest to a wide readership.

Congratulations to the authors.

I have no further comments.

We thank you, Dr. Minnerup, for reviewing our manuscript and for your positive comments.

**Reviewer #3:**

Thank you for the opportunity to review this very well-written paper evaluating the success rate of biomedical therapies' journey from animals to humans conducted as an umbrella review. This is a very important and timely review. The results are interesting and provide new evidence to inform effective and efficient translational pathways across multiple diseases.

Comments:

Quality assessment: Can you please clarify the process for developing these questions and any piloting completed to ensure the clarity of interpretation. Were any inter-rater statistics completed? Are additional details for how to apply these questions able to provided in a supplemental to allow someone else to use in the future or reproduce your scores?

We thank you for reviewing our manuscript and for your positive and constructive comments.

This critical appraisal tool was developed based on checklist proposed by Sena and colleagues (https://doi.org/10.1038/jcbfm.2014.28) and extended with additional items to enhance granularity of the critical appraisal. We added this information to the methods section which now reads:

“We assessed the quality of each included study against predefined criteria by three independent reviewers in duplicate (BVI, WEZ, and EF), based on a checklist proposed by Sena and colleagues [17], and extended with additional items for a more granular critical appraisal.” (lines 120-122)

And: “This appraisal method was initially tested in a separate umbrella review. Detailed application guidelines are available in the **Supplementary Data**.” (lines 129-130)

We also included a more detailed description of the tool in the Supplementary Data.

We did in fact calculate an inter-rater agreement (inter-rater agreement Cohen’s Kappa = 0.76) which we failed to include in the results. You can now find it in the results section (line 197). We also added a brief statement how the inter-rater agreement was calculated:

“Inter-rater agreement was calculated using Cohen’s Kappa.” (line 130-131)

It reads as though you found reviews and then did some linking of other studies based on interventions identified. Are you able to clarify these processes in text.

We agree that this process should be described in more detail. Thus, we amended an additional paragraph to the methods section outlining this two-stage process:

“**Approach to identification of therapeutic interventions**

Our method to identify therapeutic interventions assessed for bench-to-bedside translation were identified via a two-stage process: in a first step, we identified systematic/scoping reviews assessing animal-to-human translation of specific therapeutic interventions (described in the methods section “Search strategy for systematic reviews”). In the second step, these therapeutic interventions were included in our analysis of translational proportions, consistency of animal and human findings, and development times (described in the methods section “Assessment of translational proportions and development times”).” (page 4)

Line 189: Qualitative summary. There is one subsection focused on translation in different biomedical fields. However, are there other subsections? If not, I would remove the subheading.

We removed this subheading.

Line 267: Consider the role of sample size and efficacy (ideal and controlled circumstances) vs effectiveness (real world scenarios). Many interventions have been found to show promise at early clinical trial phase, but the effect is diluted when conducted on a much larger (roe generalisable) sample.

Thank you for bringing this important point to our attention. We now added a discussion about the so-called efficacy-effectiveness gap in therapy development:

“This line of reasoning also includes the so called efficacy-effectiveness gap, i.e., the differences in outcomes between patients treated in ideal and controlled circumstances of clinical trials versus real world scenarios [45, 46].” (lines 295-297)

We now also include these references:

45.	Eichler H-G, Abadie E, Breckenridge A, Flamion B, Gustafsson LL, Leufkens H, et al. Bridging the efficacy–effectiveness gap: a regulator's perspective on addressing variability of drug response. Nature reviews Drug discovery. 2011;10(7):495-506.

46.	Djulbegovic B, Paul A. From efficacy to effectiveness in the face of uncertainty: indication creep and prevention creep. Jama. 2011;305(19):2005-6.

Can you comment on the leadership of trials ie investigator initiated or industry sponsored? Does this have any role to play in the quantitative outcomes examined?

We did not systematically extract data related to the entities responsible for conducting the trials. Nonetheless, we find this aspect noteworthy, especially considering that trials initiated by researchers appear to influence clinical guidelines more than those funded by the pharmaceutical industry. In our discussion, we now propose that future research should explore whether this distinction contributes to the observed differences in translational success:

“Additionally, future studies should examine how the source of trial sponsorship, comparing investigator-initiated versus industry-sponsored trials, influences translational success, given that industry-sponsored trials appear to exert a greater influence on clinical guidelines [63].” (lines 345-348)

63.	Blümle A, Wollmann K, Bischoff K, Kapp P, Lohner S, Nury E, et al. Investigator initiated trials versus industry sponsored trials-translation of randomized controlled trials into clinical practice (IMPACT). BMC medical research methodology. 2021;21(1):182.

Limitations section: acknowledge this review is of English only studies

We added this language bias as limitation:

“Fourth, this umbrella review is confined to studies published in English, which may lead to the omission of relevant data published in other languages.” (lines 361-362)

Table 1: As well as the summary numbers, can you please list number of studies per unique disease, and include the number in any study, RCT and FDA per unique disease as well? I know the there is a lot of information in the supplemental, but it would be great if there is a way to bring any summary level (consider using advanced data visualisation options) to the main paper.

We assume you refer to the number of therapeutic interventions rather than studies per unique disease? We appreciate this suggestion and have accordingly added a figure covering these data per disease/condition. We also added the numeric data underlying this figure as a Supplementary Table that details, for each of these diseases/conditions, the number of therapeutic interventions tested, those that have progressed to any clinical study, an RCT, or received regulatory approval. Both the new figures and table again highlight that while numerous interventions proceed to clinical trials, for many diseases, including stroke, Alzheimer’s disease, and cardiac arrest, not a single intervention has secured regulatory approval (see page 24).

We also amended the results:

“Translational proportions per disease are illustrated in **Figure 4** with cardiac arrest, multiple sclerosis, and stroke assessing the most therapeutic interventions in animals (**Supplementary Table 9**).” (lines 245-247).

Spinal fusion surgeryTable 2: Positive/Negative/Neutral - please include information about this in the methods including which outcome was used to make this determination eg primary or any outcome.

We added further details on the methodology which read:

“In addition, for all clinical studies/RCTs, we extracted the main study outcome grouped into four classes , i.e., whether the intervention had a positive, negative, mixed, or neutral effect on the corresponding disease outcome [19], any outcome was considered, e.g., primary or secondary outcome.” (lines 142-145)

Line 96: add (3) in front of at full text …

We corrected this.

Line 190: consider breaking up the paragraphs for stroke, cardiac arrest and cancer for ease of reading. This may mean expanding a little for some areas.

We now separated this entire section into 4 paragraphs to enhance readability and also expanded a little on cardiac arrest and cancer (lines 225-228 and 232-233).

Recommend updating the search prior to re-submission to ensure recency, however, also appreciate the large volume of work that has gone into pulling this review together, and note the findings may not change given the nature of this review. I am happy to be guided by editorial preference and authorship team perspectives here.

We considered updating our search but ultimately chose not to proceed. As you already pointed out, the substantial workload for three individuals would likely result in only a negligible impact on the overall conclusions drawn.

I really enjoyed reading this paper and look forward to citing it in the future! Congratulations on comprehensively pulling this large body of evidence together.

Thank you again for reviewing our manuscript.
