# Supplementary material for: Analysis of animal-to-human translation shows that only 5% of animal-tested therapeutic interventions obtain regulatory approval for human applications
Source: PLoS Biol. 2024 Jun 13;22(6):e3002667. doi: 10.1371/journal.pbio.3002667 (PMC11175415; doi:10.1371/journal.pbio.3002667)
Supplement: Attachment — Submitted filename: Response_to_reviewers_R2.docx [file pbio.3002667.s022.docx]

**Point-by-point response**

Copied from the decision letter, responses in blue.

**Comments made by the Editor**

a) Please address the concerns from the (new) Academic Editor. You can find their actual comments at the foot of this email. My understanding is that they want you to 1. make it clear in the manuscript exactly how the intervention effect was classified, and 2. if you used one of the more problematic methods (e.g. relying on author conclusion), then this should be identified clearly as a limitation, with some discussion of the potential issues (spin, etc.).

Thank you again for taking time to edit and review our manuscript and for the constructive comments. We did rely on author conclusions, and we agree that this might result in an overestimation of positive (i.e., beneficial) clinical trials. We clarified this in the methods section which now reads:

“In addition, for all clinical studies/RCTs, we extracted the main study outcome as defined by the authors of the respective study, grouped into four classes , i.e., whether the intervention had a positive, negative, mixed, or neutral effect on the corresponding disease outcome [19], any outcome was considered, e.g., primary or secondary outcome.” (Page 6, lines 142-146)

Furthermore, we now acknowledge this as a limitation of our study:

“Third, the outcomes of clinical studies were classified based on the authors' conclusions, which may lead to biased interpretations if the authors frame their findings to support a beneficial therapeutic outcome [65]. This phenomenon, known as "spin," could result in an overestimation of clinical trials with beneficial outcomes.”

We added a new reference (Number 65) discussing spin of clinical study publications (Boutron et al., 2010, *JAMA*, 10.1001/jama.2010.651).

b) Please could you change your title to something that encapsulates some of the findings? We suggest something like "Evaluating the success rate of biomedical therapies from animals to humans shows a 5% regulatory approval rate after 10 years"

We changed the title to “Animal-to-human translation: only 5% of animal-tested therapeutic interventions obtain regulatory approval for human applications”

**Comments made by the new academic editor**

I read the answers to reviewers and the last version of the manuscript. I agree that the authors adequately answers all comments and revised their paper accordingly.

I must nevertheless say that I do not feel very comfortable in their classification of each study as whether the intervention had a positive, negative, mixed, or neutral effect on the corresponding disease outcome.

It seems quite unclear to me how they could do this: which outcome they considered (they state the main study outcome but often its difficult to identify or there are many and we don't know which one was prespecified), did they focus on p-value, effect estimates, confidence interval, consistency with other outcome assessed or did they use the author conclusion, which raises the issue of spin. This is slightly highlighted by reviewer 3 considering a specific study.

Thank you for taking time to review our manuscript and for the positive and constructive comments. We did rely on author conclusions, and we agree that this might result in an overestimation of positive (i.e., beneficial) clinical trials. We clarified this in the methods section which now reads:

“In addition, for all clinical studies/RCTs, we extracted the main study outcome as defined by the authors of the respective study, grouped into four classes , i.e., whether the intervention had a positive, negative, mixed, or neutral effect on the corresponding disease outcome [19], any outcome was considered, e.g., primary or secondary outcome.” (Page 6, lines 142-146)

Furthermore, we now acknowledge this as a limitation of our study:

“Third, the outcomes of clinical studies were classified based on the authors' conclusions, which may lead to biased interpretations if the authors frame their findings to support a beneficial therapeutic outcome [65]. This phenomenon, known as "spin," could result in an overestimation of clinical trials with beneficial outcomes.”

We added a new reference (Number 65) discussing spin of clinical study publications (Boutron et al., 2010, *JAMA*, 10.1001/jama.2010.651).

This approach is also questionable as the review authors "observed a progressive reduction in efficacy from animal research to early clinical trials and then to phase 3 clinical trials

Please note that this finding is based on the study by Schmidt-Pogoda and colleagues (2020, *Annals of Neurology*, 10.1002/ana.25643) and is not an observation made in our data. To clarify this misunderstanding, we rephrased the sentence which now reads:

“This trend was substantiated by a systematic review and meta-analysis which examined the preclinical-to-clinical development trajectory of 37 treatments for acute ischemic stroke, encompassing 50 phase 3 clinical trials, 75 early clinical trials, and 209 animal studies [36]: It observed a progressive reduction in efficacy from animal research to early clinical trials and then to phase 3 clinical trials.”
